# Water oxidation catalysed by iron complex of *N*,*N*′-dimethyl-2,11-diaza[3,3](2,6)pyridinophane. Spectroscopy of iron–oxo intermediates and density functional theory calculations†
†Electronic supplementary information (ESI) available: Experimental section, Table S1, Fig. S1–S34, Scheme S1 and computational details. See DOI: 10.1039/c5sc01680k


**DOI:** 10.1039/c5sc01680k

**Published:** 2015-07-22

**Authors:** Wai-Pong To, Toby Wai-Shan Chow, Chun-Wai Tse, Xiangguo Guan, Jie-Sheng Huang, Chi-Ming Che

**Affiliations:** a Department of Chemistry and State Key Laboratory of Synthetic Chemistry , The University of Hong Kong , Pokfulam Road , Hong Kong , China . Email: xgguan@hku.hk ; Email: cmche@hku.hk; b HKU Shenzhen Institute of Research and Innovation , Shenzhen 518053 , China

## Abstract

The macrocyclic [Fe^III^(L1)Cl_2_]^+^ (**1**, L1 = *N*,*N*′-dimethyl-2,11-diaza[3,3](2,6)pyridinophane) complex is an active catalyst for the oxidation of water to oxygen using [NH_4_]_2_[Ce^IV^(NO_3_)_6_] (CAN), NaIO_4_, or Oxone as the oxidant. The mechanism of **1**-catalysed water oxidation was examined by spectroscopic methods and by ^18^O-labelling experiments, revealing that Fe^IV^=O and/or Fe^V^=O species are likely to be involved in the reaction. The redox behaviour of **1** and these high-valent Fe=O species of L1 has been examined by both cyclic voltammetry and density functional theory (DFT) calculations. In aqueous solutions, the cyclic voltammograms of **1** at different pH show a pH-dependent reversible couple (*E*_1/2_ = +0.46 V *vs.* SCE at pH 1) and an irreversible anodic wave (*E*_pa_ = +1.18 V *vs.* SCE at pH 1) assigned to the Fe^III^/Fe^II^ couple and the Fe^III^ to Fe^IV^ oxidation, respectively. DFT calculations showed that the *E* value of the half reaction involving [Fe^V^(L1)(O)(OH)]^2+^/[Fe^IV^(L1)(O)(OH_2_)]^2+^ is +1.42 V *vs.* SCE at pH 1. Using CAN as the oxidant at pH 1, the formation of an Fe^IV^=O reaction intermediate was suggested by ESI-MS and UV-vis absorption spectroscopic measurements, and the rate of oxygen evolution was linearly dependent on the concentrations of both **1** and CAN. Using NaIO_4_ or Oxone as the oxidant at pH 1, the rate of oxygen evolution was linearly dependent on the concentration of **1**, and a reactive Fe^V^=O species with formula [Fe^V^(L1)(O)_2_]^+^ generated by oxidation with NaIO_4_ or Oxone was suggested by ESI-MS measurements. DFT calculations revealed that [Fe^V^(L1)(O)_2_]^+^ is capable of oxidizing water to oxygen with a reaction barrier of 15.7 kcal mol^–1^.

## Abstract 2

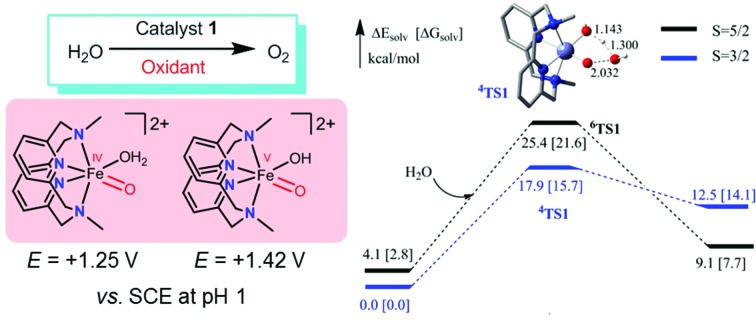
Fe^IV^=O and/or Fe^V^=O intermediates are suggested to be involved in water oxidation with [NH_4_]_2_[Ce^IV^(NO_3_)_6_], NaIO_4_, or Oxone catalyzed by [Fe^III^(L1)Cl_2_]^+^ (**1**) on the basis of spectroscopic measurements and DFT calculations.

## Introduction

Water oxidation is an energetically uphill reaction (*E* = +1.23 V *vs.* NHE at pH 0) involving the simultaneous removal of four electrons and four protons from two water molecules to give one oxygen molecule.^1^ Due to its importance, there have been tremendous efforts dedicated to the design of metal catalysts for water oxidation over the past decades.^2^ It is envisioned that mechanistic insights into the fundamental steps of this important reaction can be obtained by using structurally defined molecular catalysts.

In the literature, many examples of molecular catalysts, particularly polypyridyl ruthenium(ii) and organometallic iridium(iii) complexes, and also complexes of 1^st^-row transition metals such as manganese, iron and cobalt bearing chelating N and/or O ligands, have been reported for water oxidation.^2^ Mechanistic studies revealed that the availability of labile coordination site(s) for the formation of metal–oxo species is crucial for a metal complex to be used to catalyse water oxidation.^2^ To support the formation of reactive/oxidizing metal–oxo species, ligands that bind strongly to metal ions and are resistant to oxidation are highly desirable. In this regard, macrocyclic N-donor ligands are appealing, as highly oxidizing metal–oxo complexes of both ruthenium and iron have been isolated and structurally characterized by using macrocyclic tertiary amine ligands such as 14-TMC (1,4,8,11-tetramethyl-1,4,8,11-tetraazacyclotetradecane).^3^

Because of the high earth abundance of iron and the recent impressive advances made in the isolation and characterization of non-heme iron–oxo complexes, notably by Nam, Que, and co-workers,^4^,^5^ there has been increasing interest in the development of new iron-based oxidation chemistry, including the oxidation of water.^2^ Since the first report by Bernhard, Collins, and co-workers in 2010,6*a* several types of water oxidation reactions catalysed by mononuclear iron complexes have been reported, including chemical oxidation with CAN (cerium ammonium nitrate [NH_4_]_2_[Ce^IV^(NO_3_)_6_]) or NaIO_4_,^6^ photochemical oxidation,6c,e,^7^ electrocatalytic oxidation,^8^ and photoelectrochemical oxidation.^9^ Dinuclear iron complexes that can catalyse water oxidation with CAN or NaIO_4_,^10^ or can oxidize water to the hydroxyl radical,^11^ are also documented.

**Fig. 1:**
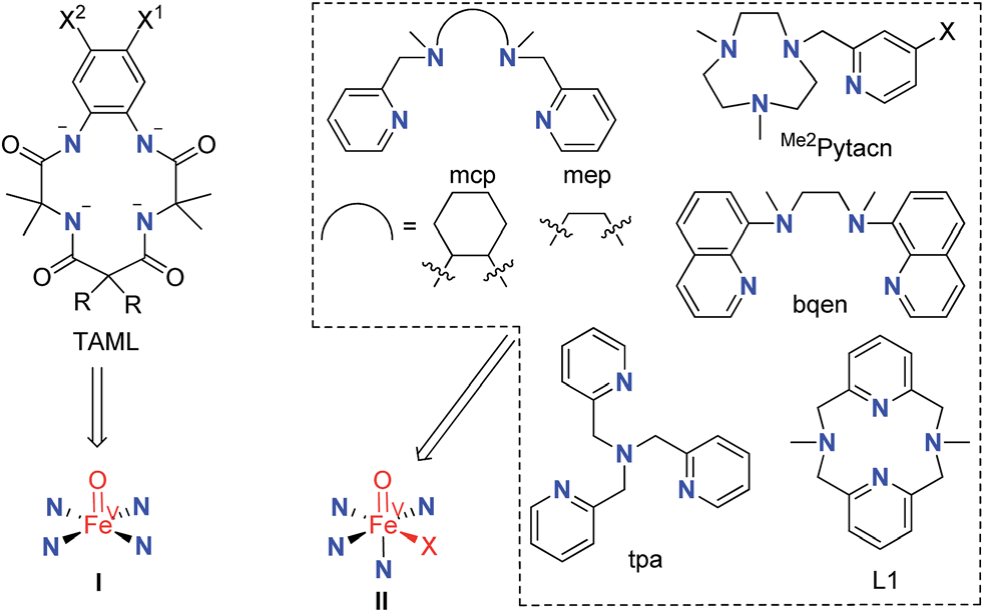
Examples of N ligands in iron complexes used as water oxidation catalysts. The corresponding proposed Fe^V^=O species are indicated. X in **II** stands for solvent, OH^–^ or another ligand.

Mononuclear iron catalysts for water oxidation are supported by tetraanionic tetraamide ligands (TAML)6a,^7^,8b or monoanionic pentadentate N_5_ ligands,8*a* or bear neutral chelating N ligands,6b–g,8c such as mcp, ^Me2^Pytacn, tpa, and bqen (Fig. 1), which allow the formation of mononuclear metal complexes with two *cis* labile sites (the abovementioned 14-TMC ligand forms *trans* complexes such as *trans*-[Fe(14-TMC)(OTf)_2_] which was found to be unreactive for catalytic water oxidation with CAN or NaIO_4_ (ref. 6*b*)). Notably, the ‘[Fe^II^(mcp)(OTf)_2_] + NaIO_4_’ system reported by Costas, Lloret-Fillol, and co-workers formed oxygen with a turnover number (TON) of up to >1000 at pH 2.6*b* Oxoiron(iv) (Fe^IV^=O) intermediates supported by ^Me2^Pytacn6b,f and bqen,6*e* generated from reaction of the corresponding iron catalysts with CAN in aqueous solution, were detected by UV-vis spectroscopy and electrospray-ionization mass spectrometry (ESI-MS); it was proposed that [Fe^IV^(bqen)(O)(OH_2_)]^2+^ may be involved in O–O bond formation,6*e* and density functional theory (DFT) calculations on the Fe^II^–^Me2^Pytacn system favoured Fe^IV^=O reactive intermediates.12*b* A recent report demonstrated the involvement of O=Fe^IV^–O–Ce^IV^ species in the water oxidation with CAN catalysed by [Fe^II^(mcp)(OTf)_2_].^13^ In most cases, oxoiron(v) (Fe^V^=O) species are proposed to be the active intermediates directly responsible for O–O bond formation in water oxidation reactions,6a,b,f,^7^,8a as revealed by DFT calculations on water oxidation with CAN catalysed by the Fe^III^–TAML,12a,d Fe^II^–^Me2^Pytacn,12e,f and Fe^II^–mep12*f* systems. Several non-heme Fe^V^=O species, which were generated by oxidation with peracids, H_2_O_2_ or ^*t*^BuOOH in organic solvents, have been reported in the literature.^14^ Notable examples include [Fe^V^(TAML)(O)]^–^,14a,j [Fe^V^(^Me2^Pytacn)(O)(OH)]^2+^,14*d* and [Fe^V^(L)(O)(S)]^3+^ (L = tpa, mep; S = H_2_O or MeCN).14*c* Recently, [Fe^V^(TAML)(O)]^–^ (**I**, Fig. 1) generated by the photochemical reaction of [Fe^III^(TAML)(H_2_O)]^–^ with ‘[Ru^II^(bipy)_3_]^2+^ + Na_2_S_2_O_8_’ in 50% MeCN–borate buffer mixture, was reported to be an active intermediate in the [Fe^III^(TAML)(H_2_O)]^–^-catalysed photochemical water oxidation.^7^ The proposed Fe^V^=O intermediates with neutral chelating N ligands (**II**, Fig. 1) remain elusive for water oxidation reactions.

*N*,*N*′-Dimethyl-2,11-diaza[3,3](2,6)pyridinophane (L1, Fig. 1),15*a* a neutral macrocyclic N_4_ ligand, is well known to form iron complexes in the *cis*-configuration, including [Fe^III^(L1)Cl_2_]^+^ (**1**).15*b* In 2010, we reported that [Fe^III^(L1)Cl_2_][FeCl_4_] (**1**·FeCl_4_) is an efficient catalyst for the *cis*-dihydroxylation of alkenes using Oxone (potassium peroxymonosulfate, 2KHSO_5_·KHSO_4_·K_2_SO_4_) as the oxidant, and the generation of an [Fe^V^(L1)(O)_2_]^+^ intermediate in the reaction was inferred from high resolution ESI-MS analysis and DFT calculations.16*a* This prompted us to examine the catalytic activity of **1** toward water oxidation. It is noted that ligand L1 has also been employed for developing the oxidation chemistry of other transition metal complexes, including [Os^III^(L1)(OH)(OH_2_)]^2+^, which can catalyse the *cis*-dihydroxylation of alkenes by H_2_O_2_*via* a reactive [Os^V^(L1)(O)(OH)]^2+^ intermediate,16*c* and [Pd^II^(L1)(Me)_2_], which reacts with oxygen to generate [Pd^III^(L1)(Me)_2_(OO˙)] followed by protonation to give [Pd^IV^(L1)(Me)_2_(OOH)]^+^.16*b* Hydrogen peroxide disproportionation catalysed by [Mn(L1)(H_2_O)_2_]^2+^ and electrochemical oxidation of water catalysed by [Mn(L1′)(H_2_O)_2_]^2+^ (L1′ = the *N*-^*t*^Bu counterpart of L1) have been reported as well.^17^

In the present work, we report the use of [Fe^III^(L1)Cl_2_]^+^ (**1**) for water oxidation with Oxone, as well as CAN and NaIO_4_, under mild conditions, together with mechanistic studies by means of high-resolution ESI-MS, UV-vis absorption spectroscopy, ^18^O-labelling experiments, kinetic studies, cyclic voltammetry, EPR analysis, and DFT calculations. During the course of this study, Sun and co-workers communicated their findings on water oxidation with CAN catalysed by [Fe^II^(L1)(MeCN)_2_]^2+^.6*g* Detailed mechanistic studies on water oxidation catalysed by Fe–L1 systems have not been reported in the literature. Also, Oxone has not been used as an oxidant in previously reported water oxidation reactions catalysed by iron complexes,^6^,^10^,^11^,^13^ despite literature reports on the manganese-catalysed oxidation of water with Oxone.2c,d The experimental studies and DFT calculations in the present work point to the generation of an Fe^V^=O species responsible for O–O bond formation in water oxidation catalysed by Fe–L1 systems.

## Results

### Water oxidation catalysed by complex **1**

At the outset, a series of iron complexes bearing N_3_, N_4_ and N_5_ ligands (*e.g.* 1,4,7-trimethyl-1,4,7-triazacyclononane, 2,2′:6′:2′′-terpyridine, L1, and 2,2′:6′,2′′:6′′,2′′′:6′′′,2′′′′-quinquepyridine) were screened for catalytic water oxidation with CAN as oxidant under different reaction conditions (Table S1 in the ESI†). We found that [Fe^III^(L1)Cl_2_][FeCl_4_] (**1**·FeCl_4_) exhibited the best performance, affording oxygen with TONs of up to 41 and 32 in 0.1 M HNO_3_ and in pure water, respectively, after 30 minutes with CAN (840 equiv.) as oxidant (Table 1, entries 1d, 1e). With NaIO_4_ as oxidant, the reaction in 0.1 M HNO_3_ afforded oxygen with TON of 12, whereas oxygen evolved with TON of only 3 from the reaction in pure water (Table 1, entries 2a and 2b). With Oxone as oxidant, under the conditions of 100 μM **1** and 84 mM Oxone in 0.1 M HNO_3_, oxygen was produced with TON of 89 in 30 minutes (Table 1, entry 3a); lowering the concentration of **1** to 12.5 μM increased the TON to 113 (Table 1, entry 3b), a value higher than those obtained using CAN or NaIO_4_ as oxidant (TONs of 93 and 44 respectively, entries 1f and 2c in Table 1). Control experiments using [Et_4_N][FeCl_4_] instead of **1**·FeCl_4_ as the catalyst did not produce detectable amounts of oxygen for the reactions with CAN and NaIO_4_, and afforded oxygen with TON of only 1.2 for the reaction with Oxone, revealing that **1** is the key species accounting for the catalytic activity of **1**·FeCl_4_. With **1**·ClO_4_ as catalyst, similar oxygen evolution to that catalysed by **1**·FeCl_4_ was observed (*e.g.* TON of O_2_: 40 *vs.* 41 with 84 mM CAN as oxidant, 11 *vs.* 12 with 84 mM NaIO_4_ as oxidant, and 91 *vs.* 89 with 84 mM Oxone as oxidant for reactions using 100 μM catalyst in 0.1 M HNO_3_).

**Table 1:** Catalytic water oxidation by **1** under different reaction conditions^*a*^

Entry	Oxidant	[Catalyst] (μM)	[Oxidant] (mM)	Medium	TON of O_2_^*b*^	OE^*c*^ (%)
1a	CAN	100	10	0.1 M HNO_3_	17	68
1b	CAN	100	20	0.1 M HNO_3_	19	38
1c	CAN	100	42	0.1 M HNO_3_	31	30
1d	CAN	100	84	0.1 M HNO_3_	41	20
1e	CAN	100	84	H_2_O	32	15
1f	CAN	12.5	125	0.1 M HNO_3_	93	4
1g	CAN	12.5	125	H_2_O	81	3
2a	NaIO_4_	100	84	0.1 M HNO_3_	12	3
2b	NaIO_4_	100	84	H_2_O	3	1
2c	NaIO_4_	12.5	125	0.1 M HNO_3_	44	1
2d	NaIO_4_	12.5	125	H_2_O	3	0.1
3a	Oxone	100	84	0.1 M HNO_3_	89	21
3b	Oxone	12.5	125	0.1 M HNO_3_	113	2

^*a*^All reactions were performed under argon atmosphere and in 0.1 M HNO_3_ or water at room temperature for 30 min.

^*b*^Determined by GC integration.

^*c*^Based on the stoichiometric ratio (1 : 4) of O_2_ formed per oxidant in 4e^–^ oxidation; oxidant efficiency (OE) = [no. of moles of O_2_ formed/(no. of moles of oxidant used/*n*)] × 100%, where *n* = 4 for CAN and *n* = 2 for NaIO_4_ and Oxone.

The origin of the oxygen gas produced from the reaction mixture of **1**-catalysed water oxidation was investigated by using a mixture of H_2_^16^O and H_2_^18^O (v/v = 1 : 1; the H_2_^18^O used had 97 atom% ^18^O) as the medium and by analysing the gaseous product(s) using GC-MS. With CAN as oxidant, the gaseous products obtained in the first 5 minutes were a mixture of ^16^O_2_/^16^O^18^O/^18^O_2_ with a ratio of 30.0 : 49.6 : 20.4, which is close to the theoretical value of 26.5 : 50.0 : 23.5 (ref. 6*b*) expected for oxygen gas derived from water. In the case of NaIO_4_ as oxidant, similar ^18^O-labelling studies using H_2_^18^O were not performed, because the oxygen atoms of IO_4_^–^ undergo rapid exchange with those of water.^18^ However, the exchange of oxygen atoms between Oxone and water is sufficiently slow for useful ^18^O-labelling studies.^19^ Water oxidation with Oxone (84 mM) in a 1 : 1 mixture of H_2_^18^O and 0.2 M HNO_3_ gave oxygen with a ^16^O_2_/^16^O^18^O/^18^O_2_ ratio of 68.9 : 29.9 : 1.2 in the first 5 minutes. This ratio falls within the range of (49–91.9) : (7.6–39) : (0.51–12) reported for water oxidation with Oxone catalysed by a Mn^III^(O)_2_Mn^IV^ complex.^19^

### Kinetic studies

**Fig. 2:**
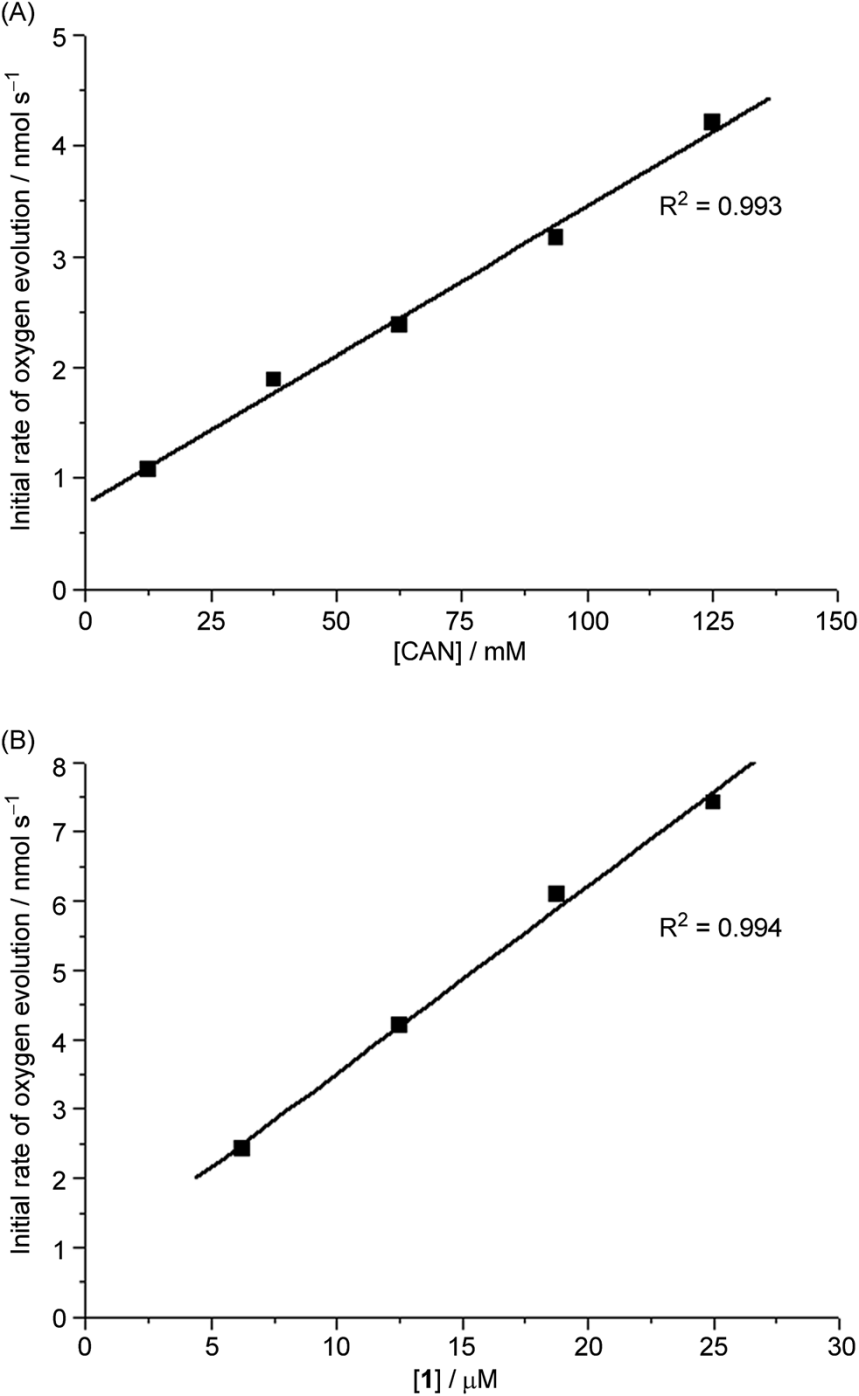
(A) Plot of initial rate of O_2_ evolution against different [CAN] (12.5–125 mM) at fixed [**1**] (12.5 μM) in 0.1 M HNO_3_. (B) Plot of initial rate of O_2_ evolution against different [**1**] (6.25–25.0 μM) at fixed [CAN] (125 mM) in 0.1 M HNO_3_.

The time courses of **1**-catalysed water oxidation with CAN in 0.1 M HNO_3_ using different concentrations of CAN (12.5–125 mM) and **1** (6.25–25 μM) were examined; the plots of oxygen evolution (measured by GC) over time are depicted in Fig. S1 and S2 in the ESI.† A linear dependence of the initial rate of oxygen evolution on both concentration of CAN and concentration of **1** was observed, as depicted in Fig. 2.

For **1**-catalysed water oxidation with NaIO_4_, the time course (0–60 min) plots of oxygen evolution at different concentrations of NaIO_4_ (12.5–125 mM) and **1** (6.25–25 μM) in 0.1 M HNO_3_ are shown in Fig. S3 and S4,† respectively. In this case, the initial rate of oxygen evolution showed a linear dependence on the concentration of **1** but not on the concentration of NaIO_4_ (see Fig. S5†).

We then examined the time courses of oxygen evolution during water oxidation with Oxone (37.5–125 mM) catalysed by **1** (6.25–25 μM) in 0.1 M HNO_3_ (Fig. S6 and S7†). Again, a linear correlation between the initial rate of oxygen evolution and the concentration of **1** was observed, whereas the initial rate was not significantly dependent on Oxone concentration (Fig. S8†), which is analogous to the findings obtained with NaIO_4_ as oxidant.

### High-resolution ESI-MS analysis

In our previous work, analysis of a reaction mixture of [Fe(L1)Br_2_]^+^ and Oxone (8 equiv.) in MeCN–H_2_O (5 : 1 v/v) by high-resolution ESI-MS revealed a cluster peak at *m*/*z* 356.0981, attributed to [Fe^V^(L1)(O)_2_]^+^.16*a* In this work, in order to detect the reaction intermediates in the oxidation reactions of **1** with CAN and NaIO_4_ in aqueous solution, we performed ESI-MS measurements on the corresponding reaction mixtures.

#### CAN as oxidant

**Fig. 3:**
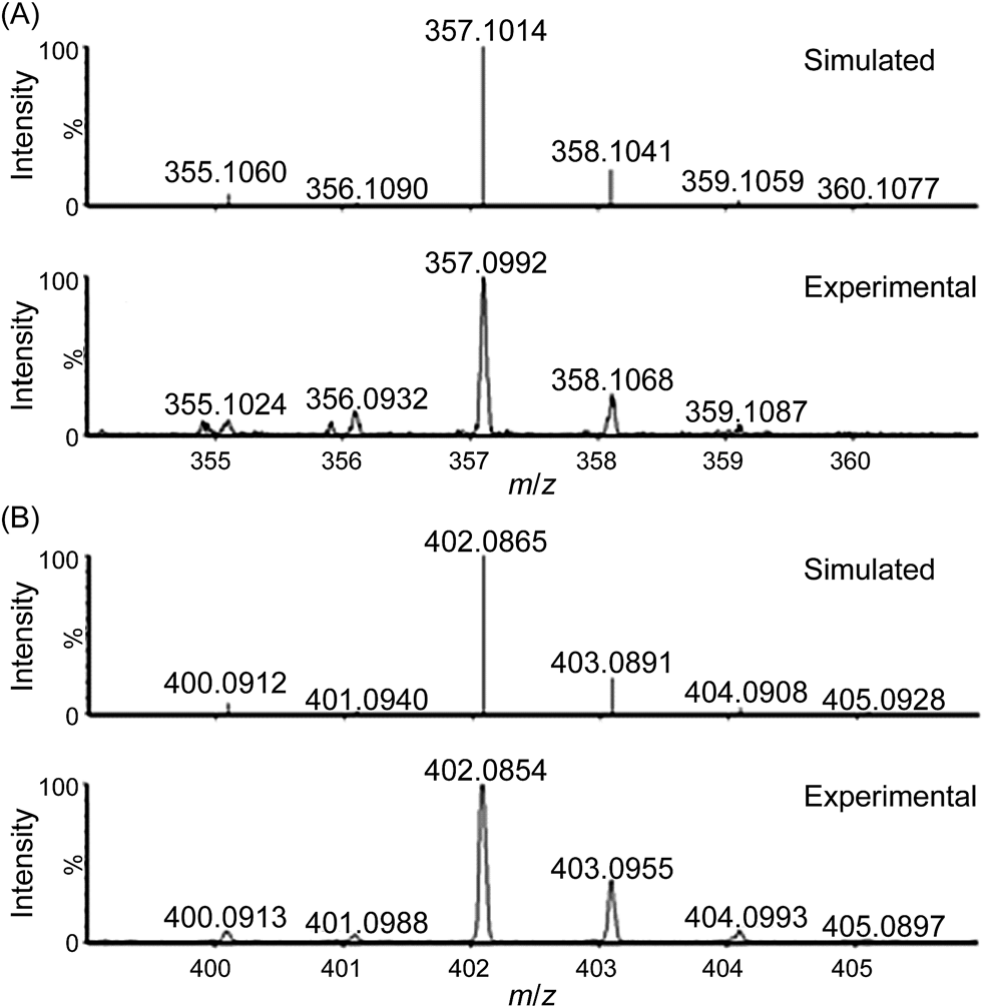
Cluster peaks at *m*/*z* 357.0992 and 402.0854 observed during high-resolution ESI-MS analysis of a reaction mixture of **1** and CAN (200 equiv.) in H_2_O. Simulated isotopic patterns for (A) [Fe^IV^(L1)(O)(OH)]^+^ and (B) [Fe^IV^(L1)(O)(NO_3_)]^+^ are shown.

Before examination of the reaction intermediates in **1**-catalysed water oxidation with CAN, high resolution ESI-MS was employed to analyse an aqueous solution of **1**, and revealed a major cluster peak at *m*/*z* 358.0906, attributed to [Fe^III^(L1)(OH)_2_]^+^ (calcd *m*/*z* 358.1092) based on the isotopic distribution, collision-induced dissociation, and ^18^O-labelling (Fig. S9–S12†). We then performed high-resolution ESI-MS analysis of a reaction mixture of **1** in H_2_O after addition of CAN (200 equiv.) for 30 seconds; the spectrum showed three new cluster peaks at *m*/*z* 357.0992, 402.0854 and 448.0815 (Fig. S13B†), assignable to [Fe^IV^(L1)(O)(OH)]^+^, [Fe^IV^(L1)(O)(NO_3_)]^+^, and [Fe^III^(L1)(NO_3_)_2_]^+^, respectively. The ESI-MS spectrum for the reaction in 0.1 M HNO_3_ (Fig. S13C†) resembles that obtained for the reaction in H_2_O. The isotopic distributions of the three new cluster peaks are depicted in Fig. 3 and in Fig. S14† (for their collision-induced dissociation, see Fig. S15 and S16†). The new cluster peaks at *m*/*z* 357.0992 and 402.0854 shifted to *m*/*z* 361.1107 and 404.0927, respectively, when the reaction of **1** with CAN (200 equiv.) was conducted in H_2_^18^O (instead of H_2_^16^O); the corresponding isotopic patterns and collision-induced dissociation spectrum obtained in the ^18^O-labelling study are shown in Fig. S17–S19.†

#### NaIO_4_ as oxidant

**Fig. 4:**
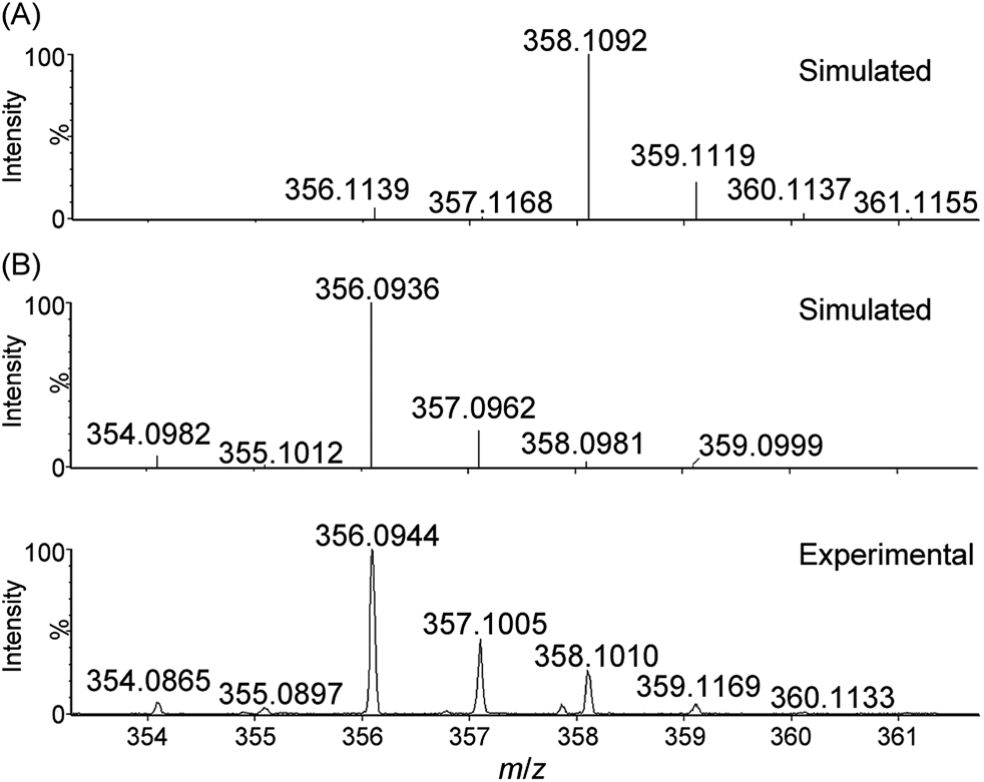
Cluster peak at *m*/*z* 356.0944 observed during high-resolution ESI-MS analysis of the reaction mixture of **1** with NaIO_4_ (800 equiv.) in 0.1 M HNO_3_. Simulated isotopic patterns for (A) [Fe^III^(L1)(OH)_2_]^+^ and (B) [Fe^V^(L1)(O)_2_]^+^ are shown.

ESI-MS analysis of a reaction mixture of **1** and NaIO_4_ (800 equiv.) in 0.1 M HNO_3_ during the first 30 seconds of the reaction revealed new species at *m*/*z* 356.0944 (Fig. 4) and 373.0940 (Fig. S20†), which can be respectively assigned to [Fe^V^(L1)(O)_2_]^+^ and [Fe^III^(L1)(OO˙)(OH)]^+^. Collision-induced dissociation spectra of the two species are depicted in Fig. S21 and S22.† When the reaction of **1** with NaIO_4_ (800 equiv.) was conducted in H_2_O under similar conditions, a new peak at *m*/*z* 356.0937 assignable to [Fe^V^(L1)(O)_2_]^+^ was formed within 10 seconds (Fig. S23†). By carrying out the same reaction in H_2_^18^O instead of H_2_^16^O, a shift of the *m*/*z* 356.0937 signal to *m*/*z* 360.1042 was observed (Fig. S24†).

### UV-vis absorption spectroscopy

**Fig. 5:**
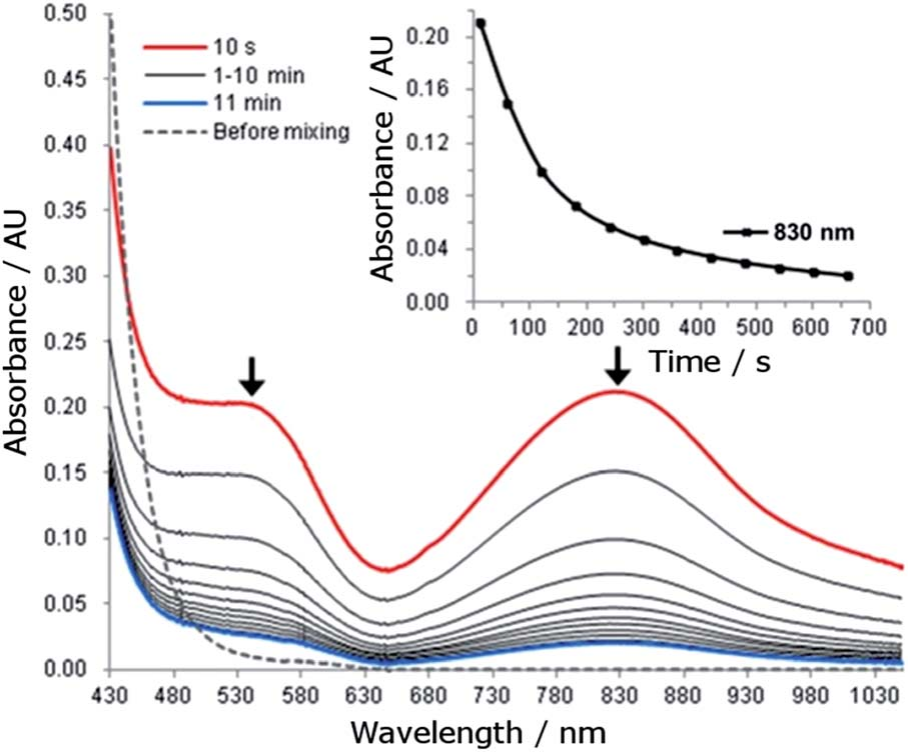
UV-vis absorption spectral changes for the reaction of **1** (1.5 mM) with CAN (7.5 mM) in 0.1 HNO_3_ at room temperature. Inset: time course of the decay of the absorption band at 830 nm.

The reaction of **1** (1.5 mM) with 5 equiv. of CAN in 0.1 M HNO_3_ at room temperature was monitored by UV-vis absorption spectroscopy. This reaction immediately (within 10 seconds) generated a new species with *λ*_max_ 830 nm and a shoulder near 540 nm (Fig. 5). The new species decayed rapidly with a half-life of 107 seconds (Fig. 5, inset).

For the reaction of **1** (1.5 mM) with 5 equiv. of NaIO_4_ or Oxone in 0.1 M HNO_3_, UV-vis measurements revealed different phenomena from that observed for the ‘**1** + CAN’ system. Upon treatment of **1** with NaIO_4_, a new species with *λ*_max_ 830 nm and a shoulder near 540 nm was gradually formed over 10 minutes (Fig. S25†) and decayed much more slowly with a half-life of ∼40 minutes (see the inset of Fig. S26†), in contrast to the CAN counterparts (Fig. 5). In the case of Oxone, the new band at ∼830 nm was barely discernible in the UV-vis spectrum of the reaction mixture, being much weaker than that observed for the reaction with NaIO_4_.

### EPR analysis

**Fig. 6:**
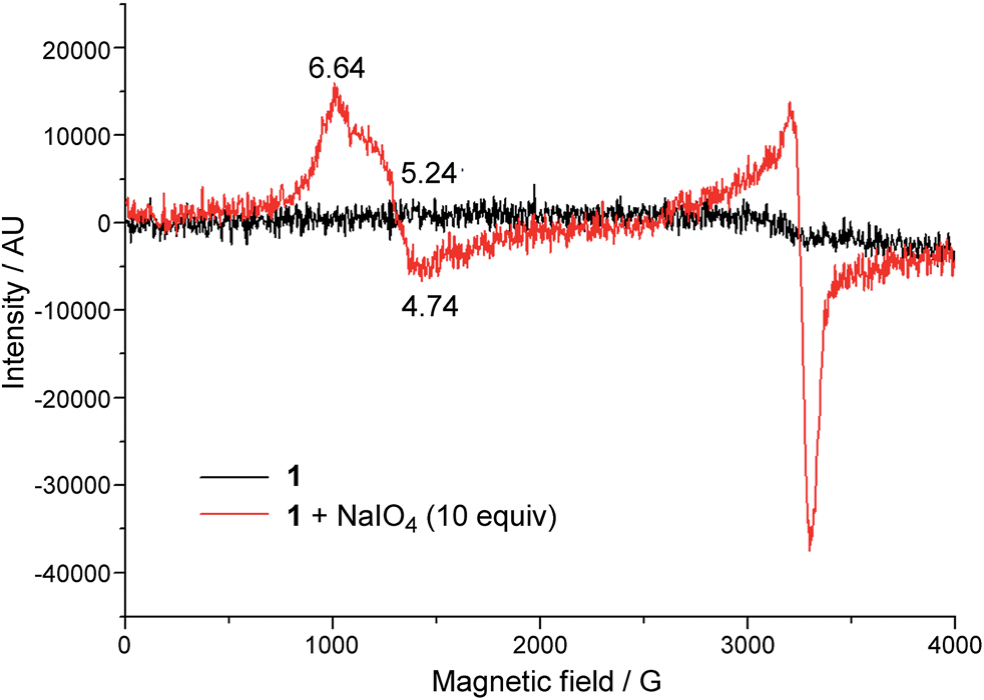
X-band EPR spectra (at 6 K) of **1**·ClO_4_ in 0.1 M HNO_3_ (black line), and a reaction mixture of **1**·ClO_4_ (1 mM) and NaIO_4_ (10 mM) in 0.1 M HNO_3_ (red line).

EPR spectroscopy was employed to examine the reaction mixture of **1**-catalysed water oxidation, using NaIO_4_ oxidant as an example. Complex **1** is a high-spin Fe^III^ complex with *S* = 5/2;15*b* however, the X-band EPR spectrum of **1**·ClO_4_ in 0.1 M HNO_3_ recorded at 6 K did not show an appreciable signal (Fig. 6, black line), presumably due to quick relaxation. The X-band EPR spectrum of the reaction mixture obtained by mixing **1**·ClO_4_ with NaIO_4_ (10 equiv.) in 0.1 M HNO_3_ at room temperature followed by immediate cooling to 6 K is depicted in Fig. 6 (red line). In this EPR spectrum, there are two prominent signals, one with *g*_avg_ = 5.54 and the other with *g* ≈ 2.

### Electrochemistry of complex **1**

**Fig. 7:**
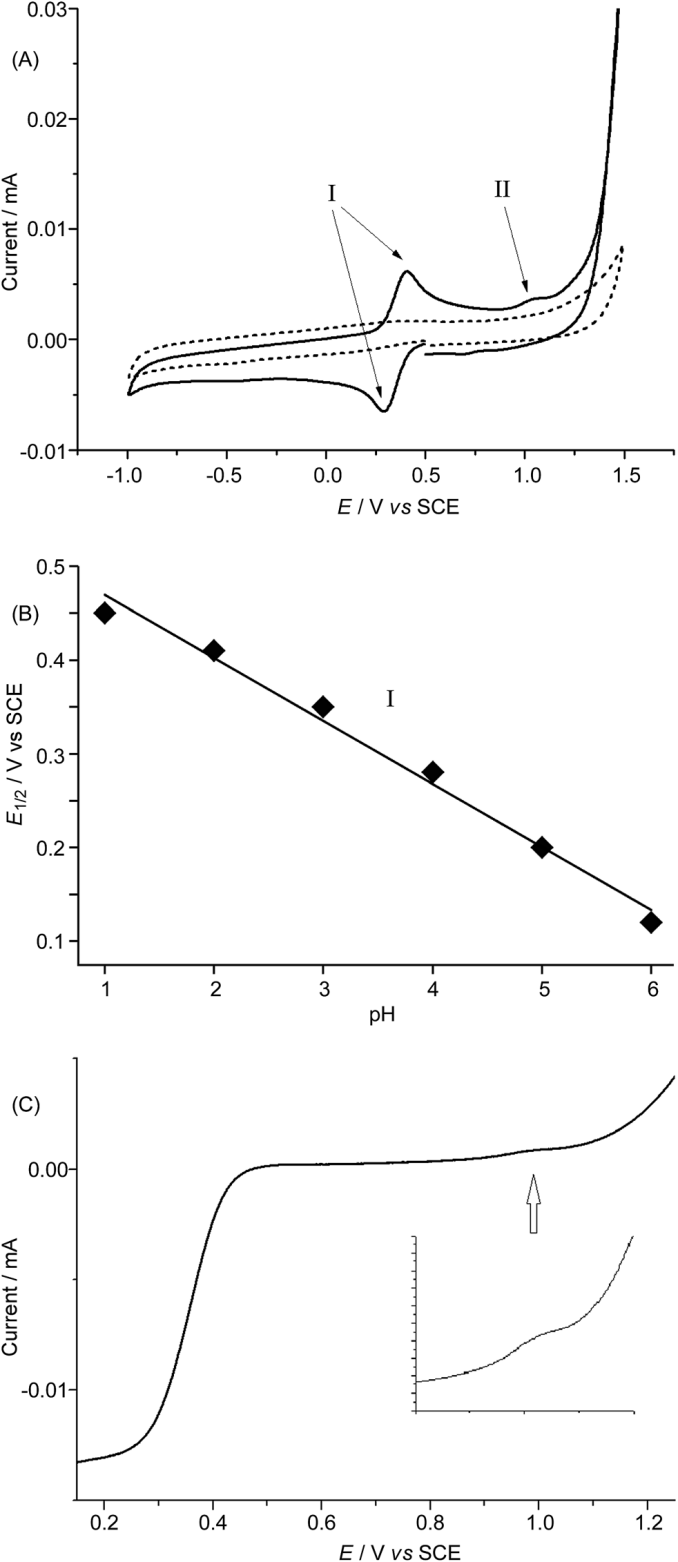
(A) Solid line: cyclic voltammogram of **1**·ClO_4_ in 0.1 M acetate buffer at pH 3; dotted line: buffer background; working electrode: glassy carbon; scan rate: 100 mV s^–1^. (B) Redox potentials (*E*_1/2_ of reversible couple I) of **1**·ClO_4_ in solutions of various pH. (C) Linear scan voltammogram of **1**·ClO_4_ in 0.1 M acetate buffer at pH 3. Working electrode: rotating glassy carbon disk; rotation rate: 100 rpm; scan rate: 5 mV s^–1^ (inset: magnified region at approximately 1.0 V).

In order to avoid interference arising from the electrochemical reactions of the FeCl_4_^–^ anion in **1**·FeCl_4_, we used the ClO_4_^–^ salt of **1** and examined its electrochemical properties in 0.1 M HNO_3_ at pH 1 and in buffered solutions at various pH by means of cyclic voltammetry and rotating disk voltammetry using glassy carbon as the working electrode. The cyclic voltammograms of **1**·ClO_4_ at pH 1–6 are depicted in Fig. 7A and S27,† and show a reversible couple I at *E*_1/2_ +0.46 V (pH 1) to +0.12 V (pH 6) *vs.* SCE, a small irreversible oxidation wave II at *E*_pa_ +1.18 V (pH 1) to +0.86 V (pH 6) *vs.* SCE, and the onset of a catalytic oxidation wave at +1.4 V (pH 1) to *ca.* +1.1 V (pH 6) *vs.* SCE. Both *E*_1/2_ of the reversible couple I and *E*_pa_ of the irreversible wave II shift cathodically by ∼67 mV per pH unit upon increasing the pH (Fig. 7B and S28†). The magnitude of the irreversible wave II was found to be sensitive to the electrode surface, but could be reproducibly observed during both the cyclic voltammetric scans and the rotating disk voltammetric experiments. The linear scan voltammograms of **1**·ClO_4_ at pH 1, 3, and 5 obtained from rotating disk voltammetric measurements are depicted in Fig. 7C and S29,† and that recorded at pH 1 at various rotation rates is shown in Fig. S30.† For example, at pH 3, a small but distinct current was recorded at *ca.* +1.05 V *vs.* SCE (Fig. 7C), which could be correlated to the small irreversible wave II in the corresponding cyclic voltammogram (Fig. 7A). Notably, the catalytic oxidation wave, presumably due to water oxidation, can be observed at pH 1 to 6 (Fig. 7A and S27†). The limiting current (*i*_L_) of the redox process corresponding to the reversible couple I increased linearly with the square root of the rotation rate (*ω*^1/2^). A plot of *i*_L_ against *ω*^1/2^ (Levich plot) shows a straight line with *R*^2^ = 0.999 (Fig. S31†). This is indicative of a totally mass-transfer-limited condition at the electrode surface. From the slope of the Levich plot, the diffusion coefficient of **1**·ClO_4_ under the experimental conditions was calculated to be 0.48 × 10^–5^ cm^2^ s^–1^.

### Density functional theory calculations

#### Electrochemical potentials of iron–oxo complexes of L1

The electrochemical potentials of iron–oxo complexes [Fe(L1)(O)(X)]^*n*+^ (X = H_2_O, HO^–^, O^2–^) in different oxidation states, together with that of the Fe^III^/Fe^II^ couple of **1**, were estimated using DFT calculations. To avoid direct calculation of the proton free energy and systematic errors, we computed the redox potentials through isodesmic reactions using the electrochemical proton-coupled electron transfer (PECT) reactions of the *cis*-(dioxo)ruthenium(vi) complex [Ru^VI^(L2)(O)_2_]^2+^ (L2 = *N*,*N*,*N*′,*N*′-tetramethyl-3,6-dimethyl-3,6-diazaoctane-1,8-diamine)^20^ as references. The isodesmic reactions are based on the assumption that both the proton free energy and the systematic errors for each pair of redox reactions (Fe^V^/Fe^IV^*vs.* Ru^V^/Ru^IV^, Fe^IV^/Fe^III^*vs.* Ru^IV^/Ru^III^, and Fe^III^/Fe^II^*vs.* Ru^III^/Ru^II^) are comparable and can be cancelled out in the calculations of ΔΔG1, ΔΔG2, and ΔΔG3 depicted in reactions (1a)–(3a) and (1b)–(3b).
1a

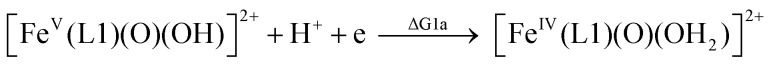



1b

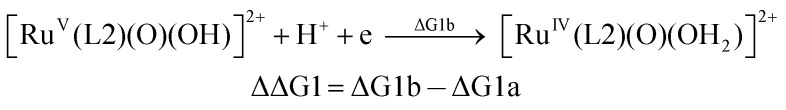



2a

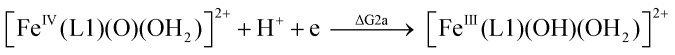



2b

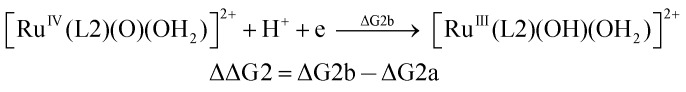



3a

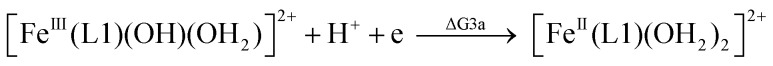



3b

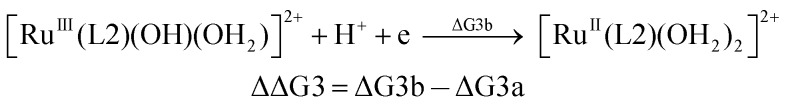




From the DFT computed ΔΔG1, ΔΔG2 and ΔΔG3 values, and the experimental redox potentials of the Ru^V^/Ru^IV^, Ru^IV^/Ru^III^ and Ru^III^/Ru^II^ couples,^20^ the redox potentials of the Fe^V^/Fe^IV^, Fe^IV^/Fe^III^ and Fe^III^/Fe^II^ couples can be calculated as:*E*(Fe^V^/Fe^IV^) = *E*(Ru^V^/Ru^IV^) + ΔΔG1*E*(Fe^IV^/Fe^III^) = *E*(Ru^IV^/Ru^III^) + ΔΔG2*E*(Fe^III^/Fe^II^) = *E*(Ru^III^/Ru^II^) + ΔΔG3where *E*(Ru^V^/Ru^IV^) = +0.72 V, *E*(Ru^IV^/Ru^III^) = +0.63 V, and *E*(Ru^III^/Ru^II^) = +0.26 V at pH 1 according to the experimental findings.^20^

**Fig. 8:**
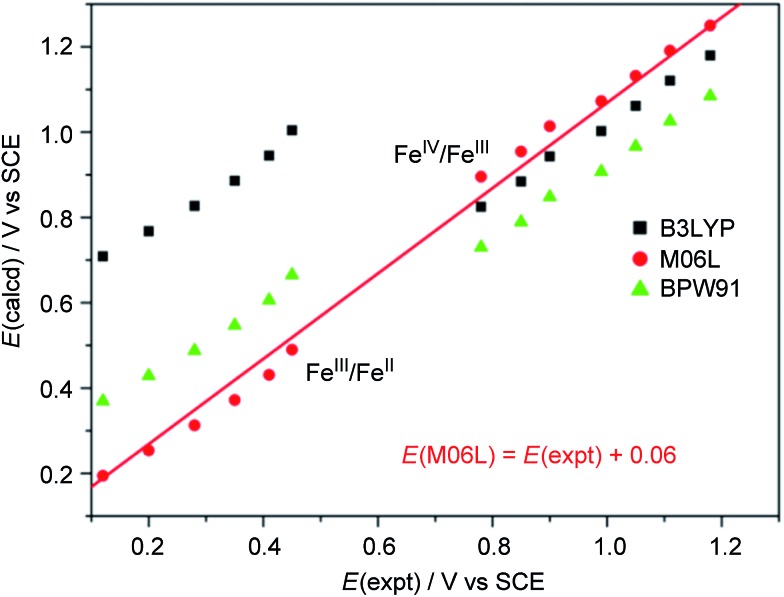
Correlation between experimental and calculated redox potentials. The *E*(expt) values for the Fe^III^/Fe^II^ and Fe^IV^/Fe^III^ couples were taken from Fig. 7 and S28† (see Discussion section). **Note of caution**: *E*(expt) values for Fe^IV^/Fe^III^ are the *E*_pa_ values of the irreversible wave II.

We calculated the Fe^III^/Fe^II^ and Fe^IV^/Fe^III^ redox potentials at different pH by using three commonly used density functionals (DFs): BPW91 (pure-GGA), B3LYP (hybrid-GGA), and M06L (*meta*-GGA). A correlation between the experimental redox potentials of Fe^III^/Fe^II^ and Fe^IV^/Fe^III^ and those calculated using these DFs is depicted in Fig. 8 (**note of caution**: calculated redox potentials are thermodynamic values). It is evident that BPW91 and B3LYP led to marked-to-severe (up to *ca.* +0.57 V) overestimation of the *E*_1/2_ of Fe^III^/Fe^II^, although B3LYP showed good performance in the prediction of the electrochemical potential of Fe^IV^/Fe^III^. Only M06L gave good estimations of the electrochemical potentials of both Fe^III^/Fe^II^ and Fe^IV^/Fe^III^ at different pH with a linear fit (*E*(M06L) = *E*(expt) + 0.06, *R* = 0.998, SD = 0.03) between the calculated redox potentials and the experimental data. Hence, M06L was chosen for the subsequent DFT calculations in this work. In the literature, the M06L functional has been reported to show good performance in modelling water oxidation by ruthenium^21^ and iron12a,c complexes.

For the calculation of the p*K*_a_ values, as an example, the p*K*_a_ of [Fe^V^(L1)(O)(OH)]^2+^ was calculated based on the following pair of isodesmic reactions (reactions (4a) and (4b)).
4a

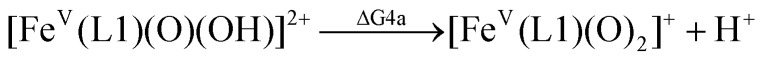



4b

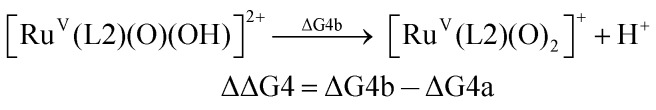

p*K*_a_([Fe^V^(L1)(O)(OH)]^2+^) = p*K*_a_([Ru^V^(L2)(O)(OH)]^2+^) – ΔΔG4/2.303*RT*where p*K*_a_([Ru^V^(L2)(O)(OH)]^2+^) = 1.8 according to the experimental study.^20^

**Scheme 1:**
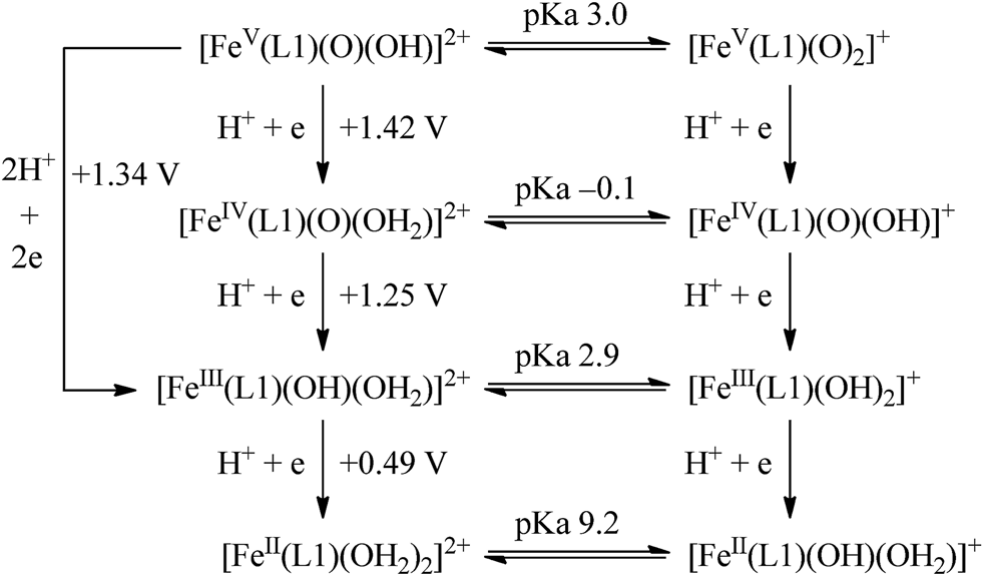
Calculated reduction potentials for Fe^V^/Fe^IV^, Fe^IV^/Fe^III^ and Fe^III^/Fe^II^, together with calculated p*K*_a_ values, based on the M06L functional.

The calculated redox potentials for various Fe^V^/Fe^IV^, Fe^IV^/Fe^III^ and Fe^III^/Fe^II^ couples, including those of [Fe(L1)(O)(X)]^*n*+^ (X = H_2_O, HO^–^, O^2–^), along with the calculated p*K*_a_ values, are depicted in Scheme 1, revealing the following features: (1) the p*K*_a_ of the [Fe^V^(L1)(O)(OH)]^2+^/[Fe^V^(L1)(O)_2_]^+^ equilibrium is 3.0; (2) the calculated *E*_1/2_ of [Fe^V^(L1)(O)(OH)]^2+^/[Fe^IV^(L1)(O)(OH_2_)]^2+^ (+1.42 V *vs.* SCE at pH 1) is comparable to the *E*_1/2_ of Ce^IV^/Ce^III^ (+1.38 V *vs.* SCE) and close to the calculated *E*_1/2_ values of [Fe^V^(L)(O)(OH)]^2+^/[Fe^IV^(L)(O)(OH_2_)]^2+^ with L = ^Me2^Pytacn or mep (+1.70 to +1.73 V *vs.* SHE,12*f* i.e. +1.46 to +1.49 V *vs.* SCE); (3) the calculated *E*_1/2_ of [Fe^IV^(L1)(O)(OH_2_)]^2+^/[Fe^III^(L1)(OH)(OH_2_)]^2+^ (+1.25 V *vs.* SCE at pH 1) matches the experimental *E*_pa_ value of Fe^IV^/Fe^III^ (*E*_pa_ = 1.18 V *vs.* SCE at pH 1, see Discussion section); (4) the calculated *E*_1/2_ of the Fe^III^/Fe^II^ couple is +0.49 V at pH 1, which matches the experimental *E*_1/2_ value for Fe^III^/Fe^II^ (+0.46 V *vs.* SCE at pH 1, see Discussion section) well; (5) the calculated *E*_1/2_ of the Fe^V^/Fe^III^ couple (*E*_1/2_ = +1.34 V *vs.* SCE at pH 1) coincides with the catalytic oxidation wave at +1.4 V observed in the cyclic voltammogram of **1** at pH 1. It is noted that there is just 170 mV difference in the calculated *E*_1/2_ values of [Fe^V^(L1)(O)(OH)]^2+^/[Fe^IV^(L1)(O)(OH_2_)]^2+^ and [Fe^IV^(L1)(O)(OH_2_)]^2+^/[Fe^III^(L1)(OH)(OH_2_)]^2+^ at pH 1.

#### Electronic properties of [Fe^V^(L1)(O)_2_]^+^

**Fig. 9:**
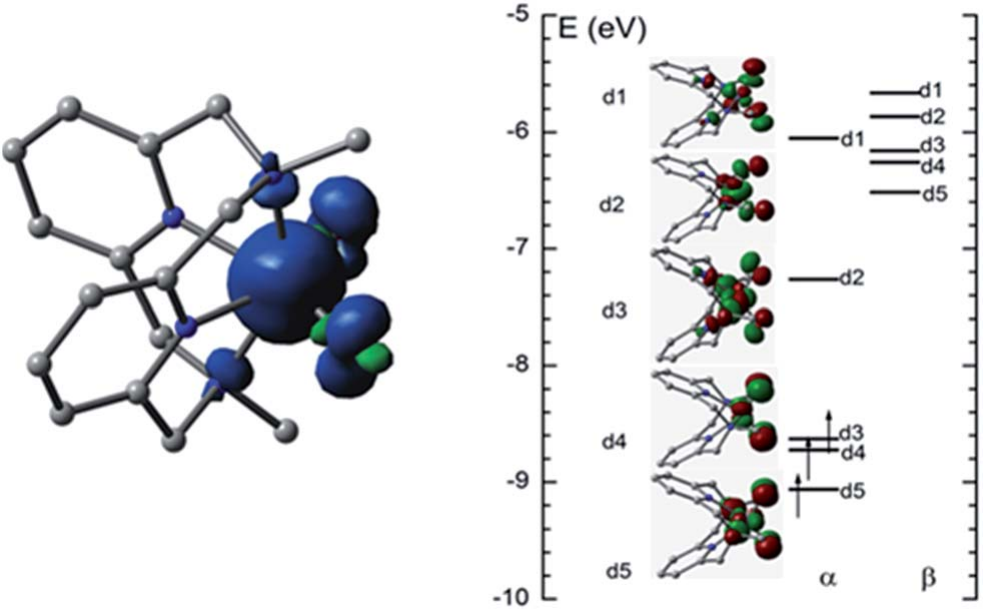
Spin density plot (left) and MO diagram (right) of [Fe^V^(L1)(O)_2_]^+^.

The ground state of [Fe^V^(L1)(O)_2_]^+^ is a quartet state with three unpaired electrons; the lowest-lying excited state is a sextet state which is 2.8 kcal mol^–1^ higher in energy than the ground state. A quartet ground state for an Fe^V^=O species has previously been predicted by DFT calculations for [Fe^V^(^Me2^Pytacn)(O)(OH)]^2+^.12f,14d The computed Fe=O distance in [Fe^V^(L1)(O)_2_]^+^ in the quartet state (**^4^Fe^V^**) is 1.614 Å, which is shorter than that in the sextet state (**^6^Fe^V^**, 1.695 Å) due to the antibonding character of the d1 and d2 orbitals (Fig. 9, right). Similar Fe=O distances were reported for [Fe^V^(L1)(O)_2_]^+^ (1.613–1.638 Å), computed at the B3LYP/6-31(G) (lanl2dz) level in our previous work, and for [Fe^V^(^Me2^Pytacn)(O)(OH)]^2+^ (1.63 Å)12*f* and low-spin [Fe^V^(TAML)(O)]^–^ (1.60 Å).14*a* The spin density plot for [Fe^V^(L1)(O)_2_]^+^ calculated in this work is shown in Fig. 9 (left). Apart from the spin density on Fe of 2.3, each oxo group has considerable spin density of 0.27. The MO diagram of [Fe^V^(L1)(O)_2_]^+^ (Fig. 9, right) reveals a d^3^ configuration in accordance with the assignment of the Fe(v) oxidation state.

#### Electronic properties of [Fe^IV^(L1)(O)(OH)]^+^

**Fig. 10:**
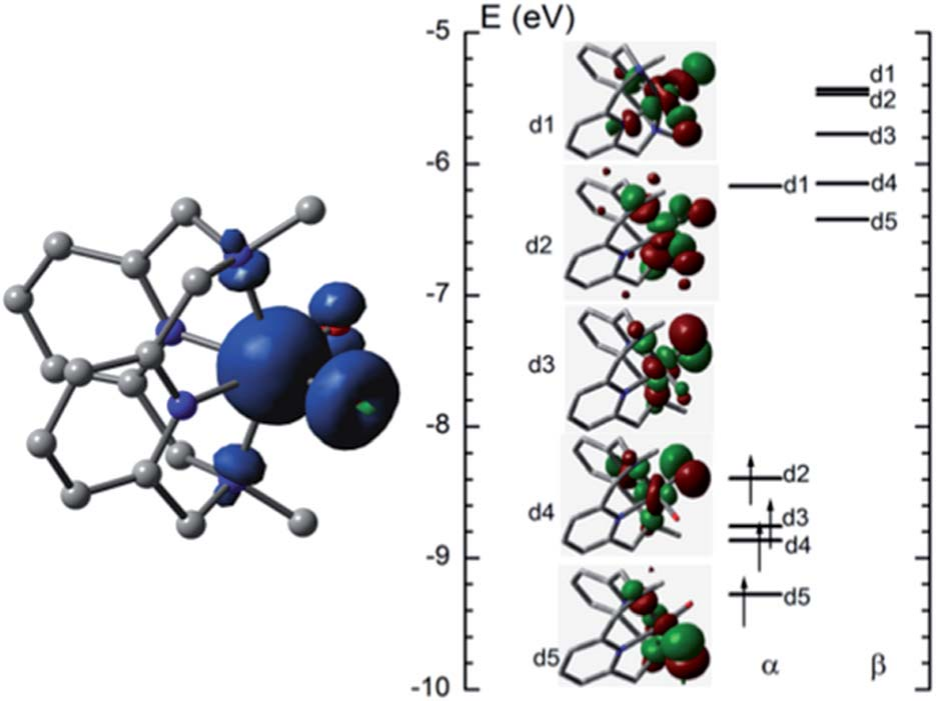
Spin density plot (left) and MO diagram (right) of [Fe^IV^(L1)(O)(OH)]^+^.

The ground state of [Fe^IV^(L1)(O)(OH)]^+^ is a quintet state with four unpaired electrons, and the triplet state is 16.0 kcal mol^–1^ higher in energy. The computed Fe=O distance of [Fe^IV^(L1)(O)(OH)]^+^ (**^5^Fe^IV^**) in the ground state is 1.632 Å, very close to the experimental Fe=O distance in [Fe^IV^(N4Py)(O)]^2+^ (1.639(5) Å).5*a*Fig. 10 (left) shows the spin density plot calculated in this work for [Fe^IV^(L1)(O)(OH)]^+^. Fe has the largest spin density of 3.1; the oxo and hydroxyl groups have spin densities of 0.59 and 0.18, respectively. The MO diagram of [Fe^IV^(L1)(O)(OH)]^+^ (Fig. 10, right) reveals a d^4^ configuration, which is consistent with the assignment of the Fe(iv) oxidation state.

#### Mechanism of water oxidation based on [Fe^V^(L1)(O)_2_]^+^

**Fig. 11:**
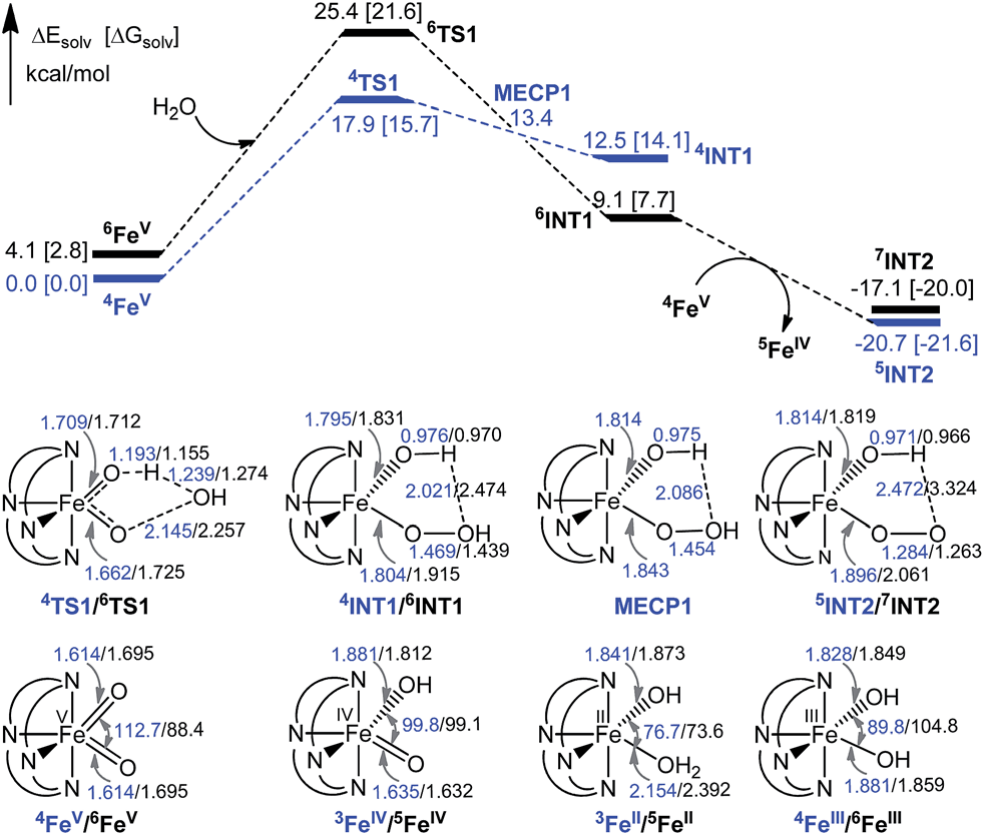
Potential energy surfaces (in kcal mol^–1^), key structural parameters (in Å and degrees) and relative stabilities (in kcal mol^–1^) of the stationary points. The energy of the MECP point (13.4 kcal mol^–1^) is the average energy of the quartet and sextet states in water.

Both the quartet (*S* = 3/2, **^4^Fe^V^**) and sextet (*S* = 5/2, **^6^Fe^V^**) states of [Fe^V^(L1)(O)_2_]^+^ were considered in the mechanism studies. The doublet state (*S* = 1/2) of [Fe^V^(L1)(O)_2_]^+^ was found to be the most energetically unfavourable (16.4 kcal mol^–1^ higher in energy than the quartet ground state) and was not considered in this work. Fig. 11 depicts the computed potential energy surfaces and key structure parameters of the stationary points. The oxidation of water starts from the reactant complex **^4^RC1/^6^RC1**, formed between **^4^Fe^V^/^6^Fe^V^** and the substrate (water molecule), which proceeds to the transition state **^4^TS1/^6^TS1** in which an O···O bond and an O···H bond are formed. The ground state is **^4^RC1**, which is 2.8 kcal mol^–1^ lower in energy than **^6^RC1**. The free energy barrier (Δ*G*^‡^) for the quartet state is 15.7 kcal mol^–1^, lower than that for the sextet state (18.9 kcal mol^–1^). Both activation barriers are comparable to those for the similar oxidation of water by [Fe^V^(TAML)(O)]^–^ (20.0 kcal mol^–1^, estimated from experimental data)12*a* and [Fe^V^(^Me2^Pytacn)(O)(OH)]^2+^ (18.8 kcal mol^–1^ obtained from DFT calculations).12*f* As the reactions proceed through the transition state, there is a reversal of the energy levels of the sextet and quartet states, with **^6^INT1** being 6.4 kcal mol^–1^ more stable than the quartet state **^4^INT1**. The location of the minimum energy crossing point (MECP) between the sextet and quartet states was found using the code developed by Harvey and co-workers.^22^ The energy of the MECP is only 0.9 kcal mol^–1^ higher than that of **^4^INT1**, suggesting that spin state reversion can easily occur, leading to the more stable complex **^6^INT1**.

The **^6^INT1** complex can subsequently be oxidized by [Fe^V^(L1)(O)_2_]^+^ (**^4^Fe^V^**) to give the superoxo complex [Fe^III^(L1)(OO˙)(OH)]^+^ (**^7^INT2/^5^INT2**). Meanwhile, **^4^Fe^V^** is reduced to [Fe^IV^(L1)(O)(OH)]^+^ (**^5^Fe^IV^**). The subsequent reaction steps include exchange of an oxygen molecule by a water molecule and the release of ^3^O_2_/^1^O_2_ (reaction (5)). The search for the transition state has not been successful. A barrier of 8.8 kcal mol^–1^ for the release of O_2_ from [TAML–Fe^IV^–OO˙]^–^ was reported by Crammer and co-workers.12*a* After release of O_2_, complex [Fe^II^(L1)(OH_2_)(OH)]^+^ (**^5^Fe^II^**) with a quintet state ground state is formed; this complex can further comproportionate with [Fe^IV^(L1)(O)(OH)]^+^ (**^5^Fe^IV^**) to regenerate two [Fe^III^(L1)(OH)_2_]^+^ (**^6^Fe^III^**) molecules.

5 [Fe^III^(L1)(OO˙)(OH)]^+^ + OH_2_ → [Fe^II^(L1)(OH_2_)(OH)]^+^ + O_2_

## Discussion

The main objective of this work is to gain insight into the mechanism of water oxidation by iron complexes of tetradentate macrocyclic ligands with a *cis* non-planar coordination geometry. The macrocyclic N_4_ ligand L1 (which is well known to form iron complexes in the *cis* configuration15*b*) was chosen, as our previous work had suggested the involvement of *in situ* generated high-valent Fe=O species of L1, including [Fe^V^(L1)(O)_2_]^+^, in the *cis*-dihydroxylation of alkenes with Oxone catalysed by [Fe^III^(L1)Cl_2_]^+^ (**1**),16*a* and Fe^V^=O species were proposed to be the reactive intermediates in iron-catalysed water oxidation reactions reported in the literature.6a,b Also, it is envisaged that the strong donor strength of L1 would stabilize iron–oxo species against demetallation in solution.

During optimization of the reaction conditions, it was noted that although the turnover number of oxygen increased with oxidant concentration, the oxidant efficiency (defined in note c of Table 1) decreased from 68% to 20% (Table 1, entries 1a–1d). Such a significant decrease in oxidant efficiency is attributed to partial decomposition/deactivation of the catalyst under strongly oxidizing reaction conditions.^23^ Similar findings were observed when NaIO_4_ or Oxone was used as oxidant.

Unlike the ‘[Fe(bqen)(OTf)_2_] + CAN’ system (which can oxidize water to O_2_ in non-buffered aqueous solution but not in 0.1 M HNO_3_ solution),6*e* the ‘**1** + CAN’ system produced oxygen with comparable TONs in water and in 0.1 M HNO_3_ (entries 1d and 1e in Table 1), consistent with the similar ESI mass spectra obtained under the two conditions (Fig. S13†). This reflects the remarkable stability of **1** toward ligand dissociation under acidic conditions, like other iron complexes such as [Fe^II^(mcp)(OTf)_2_]6*b* and [Fe^II^(L1)(MeCN)_2_]^2+^ (ref. 6*g*) used as water oxidation catalysts at pH 1. The **1**-catalysed water oxidation was sensitive to the oxidant, with the TON of oxygen evolved under the same conditions (in 0.1 M HNO_3_) following the order Oxone > CAN > NaIO_4_ (113 *vs.* 93 *vs.* 44, see Table 1, entries 1f, 2c and 3b). According to the ^16^O_2_/^16^O^18^O/^18^O_2_ ratio (68.9 : 29.9 : 1.2) obtained in the ^18^O-labelling studies, the oxygen evolved in the ‘**1** + Oxone’ system originated not only from Oxone but also from water (see the possible pathways of oxygen evolution, along with the corresponding calculated ^16^O_2_/^16^O^18^O/^18^O_2_ ratios, depicted in Scheme S1†), indicating the involvement of water oxidation in the reaction.

Considering the excellent catalytic ability of [Fe^II^(mcp)(OTf)_2_] for water oxidation, as reported by Costas, Lloret-Fillol and co-workers,6*b* we compared the activity of catalysts [Fe^II^(mcp)Cl_2_]6*b* and [Fe^III^(L1)Cl_2_]^+^ (**1**) for water oxidation. Under our reaction conditions with catalyst/oxidant = 1 : 840, the amounts of oxygen formed in [Fe^II^(mcp)Cl_2_]-catalysed water oxidation in 0.1 M HNO_3_ with CAN and NaIO_4_ as oxidants were approximately 1.4 times (TON = 56) and 2.9 times (TON = 35) the amounts formed in the **1**-catalysed reactions, respectively. Changing the catalyst/oxidant ratio from 1 : 840 to 1 : 10 000 led to an increase in the TON for oxygen formation from 41 to 93 for ‘**1** + CAN’, from 56 to 177 for ‘[Fe^II^(mcp)Cl_2_] + CAN’, from 12 to 44 for ‘**1** + NaIO_4_’, and from 35 to 114 for ‘[Fe^II^(mcp)Cl_2_] + NaIO_4_’. These results showed that [Fe^II^(mcp)Cl_2_] is more active than **1** in catalysing water oxidation.

Iron oxide nanoparticles, which can be generated from simple iron salts or iron complexes at high pH,6*c* have been shown to be efficient catalysts for water oxidation.6c,^24^ For the **1**-catalysed water oxidation with CAN, NaIO_4_, or Oxone in 0.1 M HNO_3_ (pH 1), the possibility that the observed catalytic activity of **1** is due to Fe_2_O_3_ nanoparticles generated *in situ* could be excluded, as in acidic solution either formation of Fe_2_O_3_ nanoparticles would be prevented or any Fe_2_O_3_ nanoparticles formed would be converted to Fe^3+^(aq) ions. It has been reported that Fe^3+^(aq) ions are not active in catalysing water oxidation with CAN at low pH.6*c*

Electrochemical studies of ruthenium-based molecular water oxidation catalysts often revealed strong catalytic currents due to water oxidation occurring at potentials corresponding to the generation of oxidizing Ru–oxo complexes.^25^ In general, electrochemical oxidation of Ru–OH_2_ to Ru=O species would be kinetically slow as a result of deprotonation of Ru–OH_2_ prior to oxidation. Hence the working electrode is well documented to play a crucial role in the electrochemical reversibility of the Ru=O/Ru–OH_2_ couple(s). Glassy carbon or pyrolytic carbon is commonly used as a working electrode for electrochemical studies of Ru=O complexes in aqueous solutions. While there have been numerous reports on the electrochemistry of Ru=O/Ru–OH_2_ couples in aqueous solution, to the best of our knowledge, related studies on the electrochemical oxidation of Fe–OH_2_ to Fe=O species in aqueous solution are sparse.^26^

The cyclic voltammetric and rotating disk voltammetric measurements of **1** at various pH provide useful information on the electrochemical potentials of the iron–oxo species of L1. As seen in Fig. 7B and S28,† the plots of the redox potentials against pH (from 1 to 6) for both couple I and wave II show negative slopes, and the redox potential decreases by ∼67 mV per pH unit, lending support to the two oxidation processes which are due to one electron and one proton transfer reactions. These findings, together with the experimental data obtained from rotating disk voltammetry (Fig. 7C and S29†) and the results of the DFT calculations (Fig. 8 and Scheme 1), support the assignment of couple I to the [Fe^III^(L1)(OH)(OH_2_)]^2+^/[Fe^II^(L1)(OH_2_)_2_]^2+^ redox couple and the assignment of wave II to the oxidation of [Fe^III^(L1)(OH)(OH_2_)]^2+^ to [Fe^IV^(L1)(O)(OH_2_)]^2+^. Despite the low scan rate of 5 mV s^–1^ for the rotating disk voltammetry, the magnitude of the current recorded for the [Fe^IV^(L1)(O)(OH_2_)]^2+^/[Fe^III^(L1)(OH)(OH_2_)]^2+^ oxidation wave is not the same as that recorded for the [Fe^III^(L1)(OH)(OH_2_)]^2+^/[Fe^II^(L1)(OH_2_)_2_]^2+^ redox process. As the oxidation of Fe^III^–OH to an Fe^IV^=O species involves the deprotonation of an Fe–OH unit, the electrochemical oxidation would be kinetically slow and highly sensitive to the electrode surface. Indeed, the electrochemical oxidation of Ru–OH_2_/Ru–OH to a Ru=O species is well documented to be significantly affected by the nature/surface of the working electrode.^27^ At pH 1 to 6, the difference between the observed redox potentials for wave II (Fe^IV^/Fe^III^) and couple I (Fe^III^/Fe^II^) is about 700 mV. For a ruthenium complex (also with *cis* vacant sites) supported by the mcp ligand,^28^ the difference between the redox potentials of the [Ru^IV^(mcp)(O)(OH_2_)]^2+^/[Ru^III^(mcp)(OH)(OH_2_)]^2+^ and [Ru^III^(mcp)(OH)(OH_2_)]^2+^/[Ru^II^(mcp)(OH_2_)_2_]^2+^ couples is 550 mV, which is 150 mV smaller than that between the iron analogues and can be attributed to the stronger π-bond formed in the Ru^IV^=O moiety. There is a previous report on the observation of a pH-dependent reversible couple assigned as a proton-coupled electron transfer Fe^IV^/Fe^III^ couple for [Fe^IV^(N4Py)(O)]^2+^ (N4Py = *N*,*N*-bis(2-pyridylmethyl)-bis(2-pyridyl)methylamine) in buffered aqueous solution (pH 1.5–4) with an *E*_1/2_ value of +0.41 V *vs.* SCE at pH 4 (the *E*_1/2_ value anodically shifted upon decreasing the pH with a shift of 55 mV per pH unit).^29^ However, the Fe^III^/Fe^II^ couple was not reported for [Fe^IV^(N4py)(O)]^2+^. The *E*_1/2_ value of +0.41 V for the [Fe^IV^(N4py)(O)]^2+^/[Fe^III^(N4py)(OH)]^2+^ couple in aqueous solution at pH 4 is unexpectedly low. Should this be the case, the corresponding Fe^III^/Fe^II^ couple would have to occur at a potential less than 0.0 V *vs.* SCE based on the DFT calculations in this work. Our DFT calculations using the M06L functional gave redox potentials of +1.21 V *vs.* SCE for the [Fe^IV^(N4py)(O)]^2+^/[Fe^III^(N4py)(OH)]^2+^ couple and +0.3 V *vs.* SCE for the [Fe^III^(N4py)(OH)]^2+^/[Fe^II^(N4py)(OH_2_)]^2+^ couple at pH 1. The observation of a catalytic wave for **1** (Fig. 7A and S27†) beyond the irreversible wave II (assigned to Fe^IV^/Fe^III^) at, for example, *E*_pa_ +1.18 V *vs.* SCE (pH 1), which is comparable to the DFT-calculated potential of +1.25 V *vs.* SCE (pH 1) for the [Fe^IV^(L1)(O)(OH_2_)]^2+^/[Fe^III^(L1)(OH)(OH_2_)]^2+^ couple, corroborates the finding that **1** can catalyse water oxidation through iron–oxo species at oxidation states beyond Fe^IV^.

ESI-MS analysis of a solution of **1** in water (Fig. S9–S12†), which revealed a major cluster peak assigned to [Fe^III^(L1)(OH)_2_]^+^, suggests rapid exchange of the Cl^–^ ligands of **1** with solvent. The new signals, generated upon addition of CAN, at *m*/*z* 357.0992, 402.0854, and 448.0815 can be attributed to [Fe^IV^(L1)(O)(OH)]^+^ (calcd *m*/*z* 357.1014, Fig. 3), [Fe^IV^(L1)(O)(NO_3_)]^+^ (calcd *m*/*z* 402.0865, Fig. 3) and [Fe^III^(L1)(NO_3_)_2_]^+^ (calcd *m*/*z* 448.0794, Fig. S14†), respectively, based on their *m*/*z* values and isotopic patterns, together with the ^18^O-labelling experiments which revealed new signals with *m*/*z* 361.1107 and 404.0927 attributable to [Fe^IV^(L1)(^18^O)(^18^OH)]^+^ (calcd *m*/*z* 361.1099, Fig. S17†) and [Fe^IV^(L1)(^18^O)(N^16^O_3_)]^+^ (calcd *m*/*z* 404.0907, Fig. S19†), respectively. Species [Fe^IV^(L1)(^18^O)(^16^OH)]^+^ and [Fe^IV^(L1)(^16^O)(^18^OH)]^+^ (calcd *m*/*z* 359.1057) were not detected in the experiment, indicating that the oxygen atoms of the hydroxide and oxo ligands in [Fe^IV^(L1)(O)(OH)]^+^ come from water and not from CAN.

In the UV-vis absorption spectra of the reaction mixture of **1** with CAN (Fig. 5), the new band at *λ*_max_ 830 nm is tentatively assigned to an Fe^IV^=O species of L1, with reference to the characteristic bands of Fe^IV^=O species (ranging from 700–850 nm) reported in the literature.^30^ This assignment is in line with the detection of species formulated as [Fe^IV^(L1)(O)(OH)]^+^ and [Fe^IV^(L1)(O)(NO_3_)]^+^ by ESI-MS. We further examined the intensity of the cluster peak at *m*/*z* 357.0992, attributed to [Fe^IV^(L1)(O)(OH)]^+^, at different reaction times. The ion count of this signal decreased with reaction time, as depicted in Fig. S32,† which correlates with the decay of the absorption band at *λ*_max_ 830 nm assigned to the Fe^IV^=O species in the UV-vis spectra shown in Fig. 5. Fe^IV^=O species of chelating N-donor ligands have been reported to be reasonably stable, with half-lives generally ranging from 2 to 60 hours.^30^ Unlike the [Fe^IV^(mcp)(O)(H_2_O)]^2+^ species (generated *in situ* from [Fe^II^(mcp)(OTf)_2_] and CAN in aqueous solution), which could persist under catalytic conditions with a half-life of 2.4 hours,6*b* the Fe^IV^=O species of L1 decayed with a half-life of 107 seconds under these conditions (Fig. 5). Such a fast decay may suggest that either it is taking part in a further reaction, or it is decomposing rapidly.

Given the instant generation of an Fe^IV^=O species upon oxidation of **1** with CAN in aqueous solution, as supported by ESI-MS and UV-vis analysis, together with the absence of an induction period for oxygen evolution, an Fe^IV^=O species is suggested to be involved in water oxidation by the ‘**1** + CAN’ system. Kinetic studies revealed a linear dependence of the initial rate of oxygen evolution on the concentrations of both CAN and **1** (Fig. 2; increasing [CAN] to 125 mM lowered the pH to ∼0.6, and such pH variation alone accounted for ∼18% of the rate increase depicted in Fig. 2A), which suggests that a key step of the reaction involves an iron species, presumably [Fe^IV^(L1)(O)(OH)]^+^, and one equivalent of CAN. One of the possibilities is the oxidation of [Fe^IV^(L1)(O)(OH)]^+^ by CAN to give Fe^V^=O species, such as [Fe^V^(L1)(O)(OH)]^2+^ or [Fe^V^(L1)(O)_2_]^+^, and another possibility is the reaction of [Fe^IV^(L1)(O)(OH)]^+^ with CAN to form an O=Fe^IV^–O–Ce^IV^ species similar to that recently reported for the ‘[Fe^II^(mcp)(OTf)_2_] + CAN’ system,^13^ although Fe^V^=O and O=Fe^IV^–O–Ce^IV^ species have not been clearly detected in ESI-MS analysis of the reaction mixture of **1** with CAN in H_2_O.

For water oxidation by the ‘**1** + NaIO_4_’ system, the new signals at *m*/*z* 356.0944 (major) and 373.0940 (minor), revealed by ESI-MS analysis of a mixture of **1** and NaIO_4_ in 0.1 M HNO_3_ (Fig. 4 and S20–S22†), could be assigned to [Fe^V^(L1)(O)_2_]^+^ (calcd *m*/*z* 356.0936) and [Fe^III^(L1)(OO˙)(OH)]^+^ (calcd *m*/*z* 373.0963), respectively. The assignment of [Fe^V^(L1)(O)_2_]^+^ is supported by ^18^O-labelling studies, revealing a shift of the signal at *m*/*z* 356.0944 to *m*/*z* 360.1042 attributable to [Fe^V^(L1)(^18^O)_2_]^+^ (calcd *m*/*z* 360.1021) upon changing the reaction medium to H_2_^18^O (Fig. S24†). Since IO_4_^–^ can undergo rapid oxygen exchange with water,^18^ the oxo ligands of [Fe^V^(L1)(^18^O)_2_]^+^ could come from H_2_^18^O directly and/or from ^18^O-incorporated IO_4_^–^. Changing the reaction medium from 0.1 M HNO_3_ to pure water reduced the intensity of the signal assigned to [Fe^V^(L1)(O)_2_]^+^; this observation is in line with the smaller TON of oxygen produced in pure water than in 0.1 M HNO_3_ (entries 2b *vs.* 2a, and 2d *vs.* 2c, Table 1).

The DFT calculations on [Fe^V^(L1)(O)_2_]^+^ in this work and on [Fe^V^(^Me2^Pytacn)(O)(OH)]^2+^ in the literature12f,14d revealed that the Fe^V^=O species supported by these neutral chelating N ligands all adopt a quartet ground state (*S* = 3/2), different from the doublet ground state (*S* = 1/2) of [Fe^V^(TAML)(O)]^–^ complexes, which bear tetraanionic tetraamide ligands and have been detected by EPR spectroscopy.^7^,14a,j To further test the validity of the DFT method used in this work, we performed DFT calculations on [Fe^V^(TAML)(O)]^–^ using the M06L functional, which revealed a doublet ground state for this species, consistent with previous DFT calculations reported in the literature.12a,14a To the best of our knowledge, no Fe^V^=O species with *S* = 3/2 ground state has been characterized by EPR spectroscopy. The X-band EPR spectrum of the reaction mixture of **1** with NaIO_4_ in 0.1 M HNO_3_ (Fig. 6) is dominated by two signals corresponding to *S* = 5/2 and *S* = 1/2 states; the former could be attributed to a high-spin Fe(iii) species whereas the latter probably arose from decomposed NaIO_4_, as a similar *S* = 1/2 signal was observed in the X-band EPR spectrum of a solution of NaIO_4_ in 0.1 M HNO_3_ recorded at 7 K (Fig. S33†). EPR signals of *S* = 3/2 Fe complexes are rather close to, or considerably overlap with, those of *S* = 5/2 ones.^31^ The prominent broad *S* = 5/2 signal in Fig. 6 would therefore render it difficult to clearly confirm the presence of the [Fe^V^(L1)(O)_2_]^+^ species by EPR since the signal of this *S* = 3/2 Fe^V^=O species, which is likely to have a low concentration, could easily be masked by the *S* = 5/2 signal.

Generation of [Fe^V^(L1)(O)_2_]^+^ from the oxidation of **1** with NaIO_4_ resembles the generation of [Fe^V^(L1)(O)_2_]^+^ from Oxone;16*a* both NaIO_4_ and Oxone are typically two-electron oxidants and can directly oxidize Fe(iii) to Fe(v). UV-vis spectroscopy revealed that the reaction of **1** with NaIO_4_ or Oxone is different from the reaction of **1** with CAN, as exemplified by the immediate formation of a new band at *λ*_max_ 830 nm attributable to an Fe^IV^=O species in the ‘**1** + CAN’ system (Fig. 5) but gradual formation of a much weaker band at *λ*_max_ 830 nm over 10 minutes in the ‘**1** + NaIO_4_’ system (Fig. S25†). Also, for both the ‘**1** + NaIO_4_’ and ‘**1** + Oxone’ systems, kinetic studies revealed that the initial rate of oxygen evolution showed a linear dependence on the concentration of **1** but was relatively insensitive to the concentration of NaIO_4_ (Fig. S5†) or Oxone (Fig. S8†). This supports exclusion of the possibility of an “oxo–oxo coupling” reaction between two Fe=O species.

**Scheme 2:**
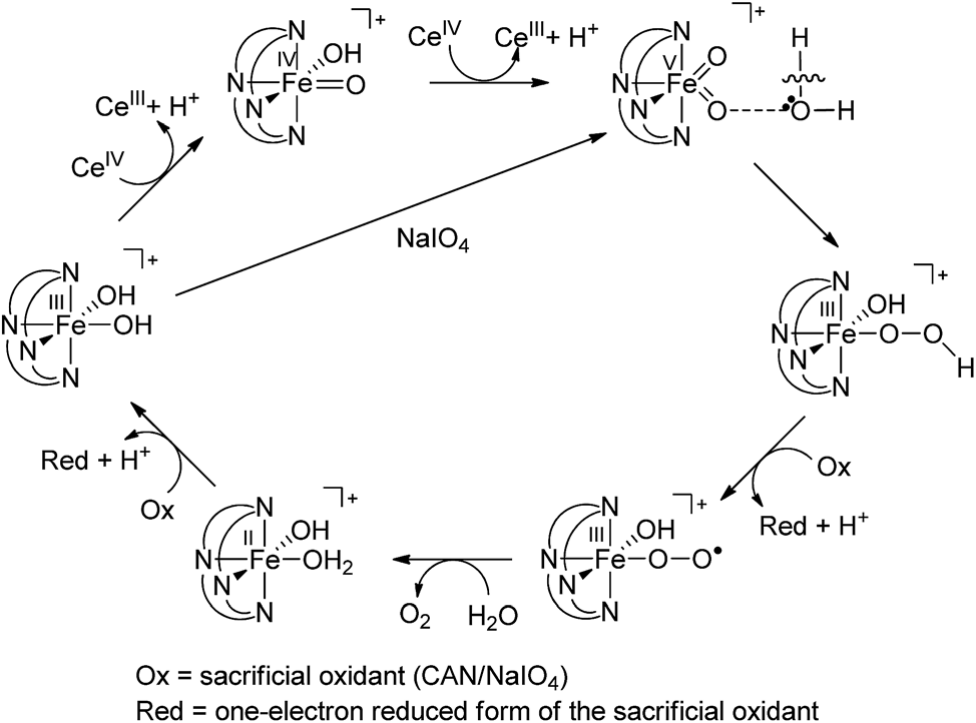
Proposed pathways for **1**-catalysed water oxidation with CAN or NaIO_4_.

On the basis of the above findings, a mechanism involving Fe^IV^=O and/or Fe^V^=O intermediate(s) for the **1**-catalysed water oxidation with CAN or NaIO_4_ is proposed as depicted in Scheme 2. For the corresponding reaction with Oxone, a mechanism similar to that of the reaction with NaIO_4_ could be proposed. The iron–oxo species depicted in Scheme 2 are [Fe^IV^(L1)(O)(OH)]^+^ and [Fe^V^(L1)(O)_2_]^+^ based on ESI-MS analysis; their protonated forms [Fe^IV^(L1)(O)(OH_2_)]^2+^ and [Fe^V^(L1)(O)(OH)]^2+^ could be involved as well (note also the computed p*K*_a_ values shown in Scheme 1), but are not included in Scheme 2. Similar to [Fe^V^(L1)(O)_2_]^+^, [Fe^V^(L1)(O)(OH)]^2+^ also adopts a quartet ground state, with the doublet state being ∼15 kcal mol^–1^ higher in energy, according to DFT calculations. The possible involvement of the Fe^V^=O reactive species in the reaction is supported by the reasonably low reaction barrier for the oxidation of water by [Fe^V^(L1)(O)_2_]^+^ of 15.7 kcal mol^–1^ obtained by the DFT calculations (Fig. 11). For [Fe^V^(L1)(O)(OH)]^2+^, the reaction barrier for O–O bond formation with water was calculated to be 21.0 kcal mol^–1^ (Fig. S34†), which is 5.3 kcal mol^–1^ higher than that calculated for [Fe^V^(L1)(O)_2_]^+^. The Fe^V^=O species is suggested to be attacked by water molecules, assisted by hydrogen-bond interactions, leading to the formation of an O–O bond, analogous to the O–O bond formation in water oxidation by Mn^V^=O,^32^ but the possibility of the involvement of Fe^IV^=O6*e* or O=Fe^IV^–O–Ce^IV^ species^13^ in the O–O bond formation could not be excluded, considering, for example, the small difference between the DFT-calculated redox potentials of +1.25 V for [Fe^IV^(L1)(O)(OH_2_)]^2+^/[Fe^III^(L1)(OH)(OH_2_)]^2+^ and +1.42 V for [Fe^V^(L1)(O)(OH)]^2+^/[Fe^IV^(L1)(O)(OH_2_)]^2+^ at pH 1. The resulting iron(iii)–peroxo intermediate in Scheme 2 is subsequently oxidized by the sacrificial oxidant (CAN, NaIO_4_, or Oxone) to give [Fe^III^(L1)(OO˙)(OH)]^+^ (DFT-calculated potential for [Fe^III^(L1)(OO˙)(OH)]^+^/[Fe^III^(L1)(OOH)(OH)]^+^: +0.64 V *vs.* SCE), the presence of which is supported by ESI-MS analysis (Fig. S20†). Extrusion of oxygen is achieved upon the substitution of [Fe^III^(L1)(OO˙)(OH)]^+^ with water; the resulting [Fe^II^(L1)(OH)(OH_2_)]^+^ is oxidized by the sacrificial oxidant (CAN, NaIO_4_, or Oxone) to regenerate the catalyst [Fe^III^(L1)(OH)_2_]^+^ (DFT-calculated potential for [Fe^III^(L1)(OH)_2_]^+^/[Fe^II^(L1)(OH)(OH_2_)]^+^: +0.26 V *vs.* SCE).

## Conclusions

A mononuclear iron(iii) complex bearing a macrocyclic N_4_ diazapyridinophane ligand catalyses the oxidation of water to oxygen with NaIO_4_ or Oxone, as well as CAN, as the oxidant in acidic and/or neutral aqueous media. Studies using kinetic measurements, high-resolution ESI-MS, UV-vis absorption spectroscopy, ^18^O-labelling experiments, cyclic voltammetry, and DFT calculations lend support to the possible involvement of high-valent iron(iv)–oxo species such as [Fe^IV^(L1)(O)(OH)]^+^ and/or iron(v)–oxo species such as [Fe^V^(L1)(O)_2_]^+^, or their protonated forms [Fe^IV^(L1)(O)(OH_2_)]^2+^ and/or [Fe^V^(L1)(O)(OH)]^2+^, in the **1**-catalysed water oxidation reactions.

## Supplementary Material

Supplementary information
