# Supplementary material for: Water oxidation catalysed by iron complex of N,N′-dimethyl-2,11-diaza[3,3](2,6)pyridinophane. Spectroscopy of iron–oxo intermediates and density functional theory calculations
Source: Chem Sci. 2015 Jul 22;6(10):5891–903. doi: 10.1039/c5sc01680k (PMC5950833; doi:10.1039/c5sc01680k)
Supplement: Supplementary file 1 [file SC-006-C5SC01680K-s001.pdf]

Electronic Supplementary Material (ESI) for Chemical Science.
This journal is © The Royal Society of Chemistry 2015

Electronic Supplementary Information

Table of Contents

Experimental Section

Preparation of iron complexes S3

Instrumentation S3

General procedures for water oxidation S4

Data treatment and analysis S4

Computational details S5

References S6

Table S1 Water oxidation catalysed by iron complexes with CAN in 0.1 M HNO_3 S7

Fig. S1 Plot of O_2 evolution in **1**-catalysed water oxidation with CAN in 0.1 M HNO_3 with time for different concentrations of CAN S8

Fig. S2 Plot of O_2 evolution in **1**-catalysed water oxidation with CAN in 0.1 M HNO_3 with time for different concentrations of **1** S8

Fig. S3 Plot of O_2 evolution in **1**-catalysed water oxidation with $NaIO_4$ in 0.1 M HNO_3 with time for different concentrations of $NaIO_4$ S9

Fig. S4 Plot of O_2 evolution in **1**-catalysed water oxidation with $NaIO_4$ in 0.1 M HNO_3 with time for different concentrations of **1** S9

Fig. S5 Plots of initial rate of O_2 evolution in 0.1 M HNO_3 against different concentrations of [$NaIO_4$] and [**1**] S10

Fig. S6 Plot of O_2 evolution in **1**-catalysed water oxidation with Oxone in 0.1 M HNO_3 with time for different concentrations of **1** S11

Fig. S7 Plot of O_2 evolution in **1**-catalysed water oxidation with Oxone in 0.1 M HNO_3 with time for different concentrations of Oxone S11

Fig. S8 Plots of initial rate of O_2 evolution in 0.1 M HNO_3 against different concentrations of [Oxone] and [**1**] S12

Fig. S9 ESI mass spectrum of **1**·$FeCl_4$ in H_2O S13

Fig. S10 Collision-induced dissociation of $[Fe^{III}(L1)(OH)_2]^+$ generated in the solution of **1** in H_2O S14

Fig. S11 Collision-induced dissociation of $[Fe^{III}(L1)(OH)Cl]^+$ generated in the solution of **1** in H_2O S15

Fig. S12 Electrospray ionization mass spectrum of **1** in $H_2{}^{18}O$ S16

Fig. S13 ESI-MS analysis of the reaction mixture of **1** with CAN in H_2O and in 0.1 M HNO_3 S17

Fig. S14 ESI-MS measured isotopic pattern for the cluster peak at *m/z* 448.0815 (for reaction of **1** with CAN in 0.1 M HNO_3) as compared with simulated isotopic pattern for $[Fe^{III}(L1)(NO_3)_2]^+$ S18

Fig. S15 Collision-induced dissociation of the ion with *m/z* 357.0992 (for the reaction of **1** with CAN in 0.1 M HNO_3) S18

Fig. S16 Collision-induced dissociation of the ion at *m/z* 402.0854 (for the reaction of **1** with CAN in 0.1 M HNO_3) S19

**Fig. S17**	ESI-MS measured isotopic pattern for the cluster peak at *m/z* 361.1107 (for reaction of **1** with CAN in $H_2^{18}O$) as compared with simulated isotopic pattern for $[Fe^{IV}(L1)(^{18}O)(^{18}OH)]^+$	S19
**Fig. S18**	Collision-induced dissociation of the ion with *m/z* 361.1107 (for the reaction of **1** with CAN in $H_2^{18}O$)	S20
**Fig. S19**	ESI-MS measured isotopic pattern for the cluster peak at *m/z* 404.0927 (for the reaction of **1** with CAN in $H_2^{18}O$) as compared with simulated isotopic patterns for $[Fe^{III}(L1)(^{18}OH)(N^{16}O_3)]^+$ and $[Fe^{IV}(L1)(^{18}O)(N^{16}O_3)]^+$	S21
**Fig. S20**	ESI-MS measured isotopic pattern for the cluster peak at *m/z* 373.0940 (for the reaction of **1** with $NaIO_4$ in 0.1M HNO_3) as compared with simulated isotopic patterns for $[Fe^{III}(L1)(OO^{\bullet})(OH)]^+$ and $[Fe^{IV}(L1)(OO^{\bullet})(O)]^+$	S22
**Fig. S21**	Collision-induced dissociation of the ion with *m/z* 356.0944 (for the reaction of **1** with $NaIO_4$ in 0.1 M HNO_3)	S23
**Fig. S22**	Collision-induced dissociation of the ion with *m/z* 373.0940 (for the reaction of **1** with $NaIO_4$ in 0.1 M HNO_3)	S24
**Fig. S23**	ESI-MS measurements of the reaction mixture of **1** with $NaIO_4$ (800 equiv) in H_2O at different reaction time (0–15 sec)	S25
**Fig. S24**	ESI-MS measured isotopic pattern for the cluster peak at *m/z* 360.1042 (for the reaction of **1** with $NaIO_4$ in $H_2^{18}O$) as compared with simulated isotopic patterns for $[Fe^{III}(L1)(^{18}OH)_2]^+$, $[Fe^{IV}(L1)(^{18}O)(^{18}OH)]^+$, and $[Fe^{V}(L1)(^{18}O)_2]^+$	S26
**Fig. S25**	UV-vis absorption spectral changes for the reaction of **1** with $NaIO_4$ in 0.1 M HNO_3 at room temperature at reaction times of 1−10 min	S27
**Fig. S26**	UV-vis absorption spectral changes for the reaction of **1** with $NaIO_4$ in 0.1 M HNO_3 at room temperature at reaction times of 10–90 min and time course of the decay monitored at 830 nm	S28
**Fig. S27**	Cyclic voltammograms of **1**·ClO_4 in aqueous solutions at various pH	S29
**Fig. S28**	Redox potentials (the irreversible wave II) of **1**·ClO_4 in aqueous solutions at various pH	S30
**Fig. S29**	Linear scan voltammograms of **1**·ClO_4 in aqueous solutions at pH 1 and at pH 5	S31
**Fig. S30**	Linear scan voltammogram of **1**·ClO_4 in 0.1 M HNO_3 (pH 1) at various rotation rates	S32
**Fig. S31**	Levich plot for the limiting current of the redox process corresponding to the reversible couple I	S33
**Fig. S32**	Ion counts of the ESI-MS signal at *m/z* 357.1 assigned to $[Fe^{IV}(L1)(O)(OH)]^+$ at different reaction times	S34
**Fig. S33**	X-band EPR spectrum (at 7 K) of $NaIO_4$ in 0.1 M HNO_3	S35
**Fig. S34**	Comparison of potential energy surfaces of the first step of water oxidation based on $[Fe^{V}(L1)(O)_2]^+$ and $[Fe^{V}(L1)(O)(OH)]^{2+}$	S36
**Scheme S1**	Possible pathways for O_2 evolution in the reaction mixture of **1** with Oxone in $H_2^{16}O/H_2^{18}O$.	S37
**Cartesian coordinates for optimized structures of possible intermediates/species involved in mechanistic studies**		S38

Experimental Section

Preparation of iron complexes

All iron complexes examined in this work for water oxidation were prepared according to literature procedures.

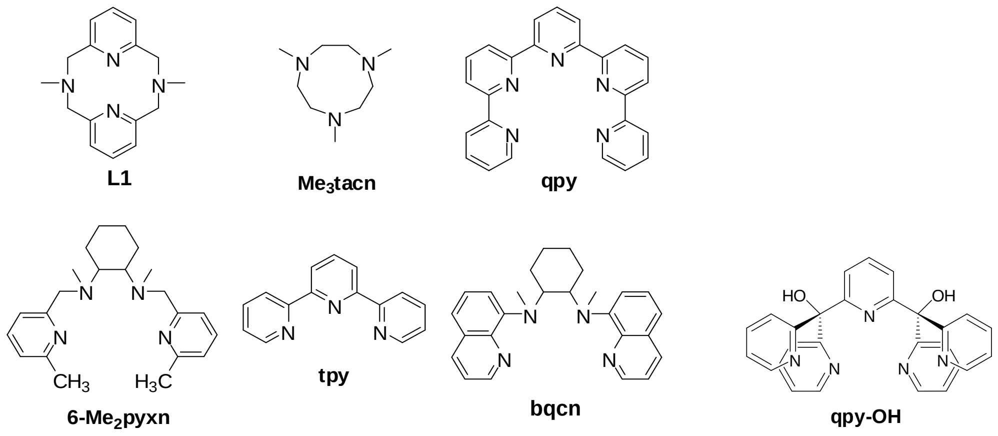

Complex	Reference
$[Fe^{III}(L1)Cl_2][FeCl_4]$	T. W.-S. Chow, E. L.-M. Wong, Z. Guo, Y. Liu, J.-S. Huang and C.-M. Che, *J. Am. Chem. Soc.*, 2010, **132**, 13229.
$[Fe^{III}(Me_3tacn)Cl_3]$	P. Chaudhuri, M. Winter, K. Wieghardt, S. Gehring, W. Haase, B. Nuber and J. Weiss, *Inorg. Chem.*, 1988, **27**, 1564.
$[Fe^{II}(qpy)(MeCN)_2](ClO_4)_2$	E. L.-M. Wong, G.-S. Fang, C.-M. Che and N. Zhu, *Chem. Commun.*, 2005, 4578.
$[Fe^{II}(6\text{-}Me_2pyxn)Cl_2]$	M. Costas and L. Que Jr., *Angew. Chem. Int. Ed.*, 2002, **41**, 2179.
$[Fe^{II}(tpy)_2](ClO_4)_2$	P. Liu, E. L.-M. Wong, A. W.-H. Yuen and C.-M. Che, *Org. Lett.*, 2008, **10**, 3275.
$[Fe^{III}(bqcn)Cl_2][FeCl_4]$	W.-S. Chow, PhD thesis, The University of Hong Kong, 2010.
$[Fe^{II}(qpy\text{-}OH)(MeCN)](ClO_4)_2$	E. L.-M. Wong, G.-S. Fang, C.-M. Che and N. Zhu, *Chem. Commun.*, 2005, 4578.

Instrumentation

UV-visible absorption spectra were obtained by using HP 8453 diode array spectrophotometer. Analysis of gas composition was done by using Agilent 7890A

GC equipped with a 5 Å molecular sieve column and a thermal conductivity detector. Argon was used as both carrier and reference gas. Cyclic voltammetry was conducted on a Princeton Applied Research Model 273A Potentiostat. Rotating disk voltammetry was performed using a Princeton Applied Research PMC-1000 Potentiostat; with a modulated speed rotator from PINE Research Instrumentation. The working electrode was glassy carbon; the reference electrode was SCE; the counter electrode was a platinum wire/coil. All potentials are reported versus SCE (+0.241 V *vs.* NHE). ESI-MS measurements were performed on a Waters Micromass Q-Tof Premier quadrupole time-of-flight tandem mass spectrometer. X-band EPR spectra were obtained using a Bruker EMX EPR spectrometer equipped with a variable-temperature helium flow cryostat system (Oxford Instruments).

General procedures for water oxidation

All reactions were carried out in a 10-mL round-bottom flask equipped with a magnetic stirrer bar and a rubber septum. The flask was first added with 3 mL of aqueous solution (in either distilled H_2O or 0.1 M HNO_3) containing the water-oxidation catalyst. The solution and the flask were then degassed by bubbling argon gas for 2 min. An aqueous solution (1 mL, in either distilled H_2O or 0.1 M HNO_3) containing the oxidant was injected into the flask through the rubber septum and the reaction mixture was stirred at room temperature throughout the experiment. Oxygen generation was monitored by taking 50 μL of headspace of the reaction flask by a Pressure-Lok® glass syringe and injecting it into the GC. The amount of oxygen generated was determined by using the external standard method.

Data treatment and analysis

The signal for oxygen was corrected by subtracting the oxygen signal due to the presence of air in the dead volume of syringe or leakage, which could be calculated from the signal for nitrogen:

$$\text{Corrected } O_2 \text{ signal} = \text{observed } O_2 \text{ signal} - \text{observed } N_2 \text{ signal} / (R_{N2/O2})$$

where $R_{N2/O2}$ is the ratio of signals of N_2 to O_2 of air, which is equal to 2.93. This value was obtained by analysing 50 μL of air samples and is an average of 5 trials.

A calibration curve was obtained by injecting a known volume of oxygen gas into a 10-mL round-bottom flask with 4 mL of water, a magnetic stirrer, and a rubber

septum under argon. After injecting oxygen gas into the flask, the solution was stirred for 10 min to ensure that the oxygen gas is evenly distributed in the flask. 50 μL of headspace of the reaction flask were taken out by syringe and analysed by GC.

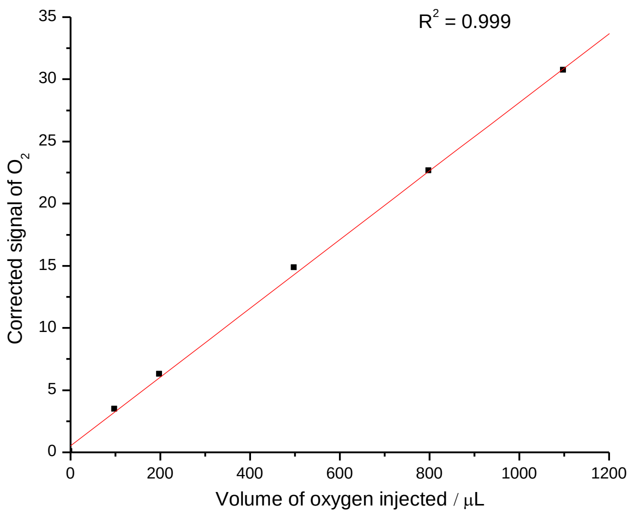

The amount of oxygen produced was calculated by using the above calibration curve. The initial rate of oxygen evolution was estimated by a least-square linear fit of the data points at time = 1, 5, and 10 min, and the y-intercept was set to 0.

Computational details

All the DFT calculations were performed using Gaussian 09 program suite.[1] The meta-GGA M06L functional was used to probe the reaction pathway of water oxidation. Double-zetta basis set (6-31+G*(SDD))[2] was employed for geometry optimization and triplet-zetta with diffused function (6-311+G(2df,p)(SDD)) was used for singlet point energy correction. DFs [BPW91 (pure-GGA), B3LYP (hybrid-GGA), and M06L (meta-GGA))] were used for evaluation through comparing the calculated and the experimental redox potentials. The Solvent effects were studied using self-consistent reaction field (SCRF) method based on the PCM (IEFPCM) models.[3] The choice of solvents (water, dielectric constant e = 78.35) was based on the solvent media for electrochemistry experiments. The defaulted atomic radii (UFF) were used in the PCM calculations, which included the individual spheres for hydrogens to build up the solvation cavity. Vibrational analysis has been performed for all the stationary points to characterize the transition state (one imaginary frequency) and the corresponding reactant complex (no imaginary frequency) and product complex (no imaginary frequency). IRC calculations near the transition state were performed to verify the transition state.[4]

References

1 M. J. Frisch, G. W. Trucks, H. B. Schlegel, G. E. Scuseria, M. A. Robb, J. R. Cheeseman, G. Scalmani, V. Barone, B. Mennucci, G. A. Petersson, H. Nakatsuji, M. Caricato, X. Li, H. P. Hratchian, A. F. Izmaylov, J. Bloino, G. Zheng, J. L. Sonnenberg, M. Hada, M. Ehara, K. Toyota, R. Fukuda, J. Hasegawa, M. Ishida, T. Nakajima, Y. Honda, O. Kitao, H. Nakai, T. Vreven, J. A. Montgomery, Jr., J. E. Peralta, F. Ogliaro, M. Bearpark, J. J. Heyd, E. Brothers, K. N. Kudin, V. N. Staroverov, R. Kobayashi, J. Normand, K. Raghavachari, A. Rendell, J. C. Burant, S. S. Iyengar, J. Tomasi, M. Cossi, N. Rega, J. M. Millam, M. Klene, J. E. Knox, J. B. Cross, V. Bakken, C. Adamo, J. Jaramillo, R. Gomperts, R. E. Stratmann, O. Yazyev, A. J. Austin, R. Cammi, C. Pomelli, J. W. Ochterski, R. L. Martin, K. Morokuma, V. G. Zakrzewski, G. A. Voth, P. Salvador, J. J. Dannenberg, S. Dapprich, A. D. Daniels, Ö. Farkas, J. B. Foresman, J. V. Ortiz, J. Cioslowski and D. J. Fox, *Gaussian 09, Revision D.01*, Gaussian, Inc., Wallingford CT, 2013.

2 M. Dolg, H. Stoll, H. Preuss and R. M. Pitzer, *J. Phys. Chem.*, 1993, **97**, 5852.

3 (*a*) O. Tapia, *J. Math. Chem.*, 1992, **10**, 139; (*b*) J. Tomasi and M. Persico, *Chem. Rev.*, 1994, **94**, 2027.

4 (*a*) C. Gonzalez and H. B. Schlegel, *J. Chem. Phys.*, 1989, **90**, 2154; (*b*) C. Gonzalez and H. B. Schlegel, *J. Phys. Chem.*, 1990, **94**, 5523.

Table S1 Water oxidation catalysed by iron complexes with CAN in 0.1 M HNO_3.

Entry	Iron complex[a]	[Complex]/[CAN]	TON of O_2
1	$[Fe^{III}(L1)Cl_2][FeCl_4]$ (**1**·$FeCl_4$)	1:840	41
2	$[Fe^{III}(Me_3tacn)Cl_3]$	1:840	0.6
3	$[Fe^{II}(qpy)Cl_2]$	1:840	0.1
4	$[Fe^{II}(6\text{-}Me_2pyxn)Cl_2]$	1:840	0.4
5	$[Fe^{II}(tpy)_2][ClO_4]_2$	1:840	1.2
6	$[Fe^{III}(bqcn)Cl_2][FeCl_4]$	1:840	6
7	$[Fe^{II}(qpy\text{-}OH)(MeCN)Cl_2]$	1:840	1.6

[a] For the structural formulas of the corresponding ligands, see Page S3.

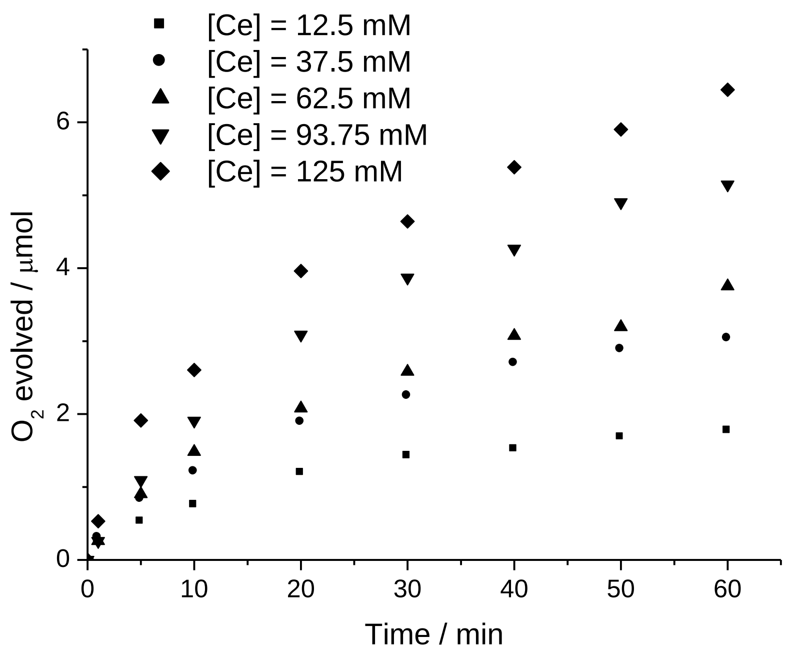

Fig. S1 Plot of O_2 evolution in **1**-catalysed water oxidation with CAN in 0.1 M HNO_3 with time for different concentrations of CAN. Concentration of **1**: 12.5 μM.

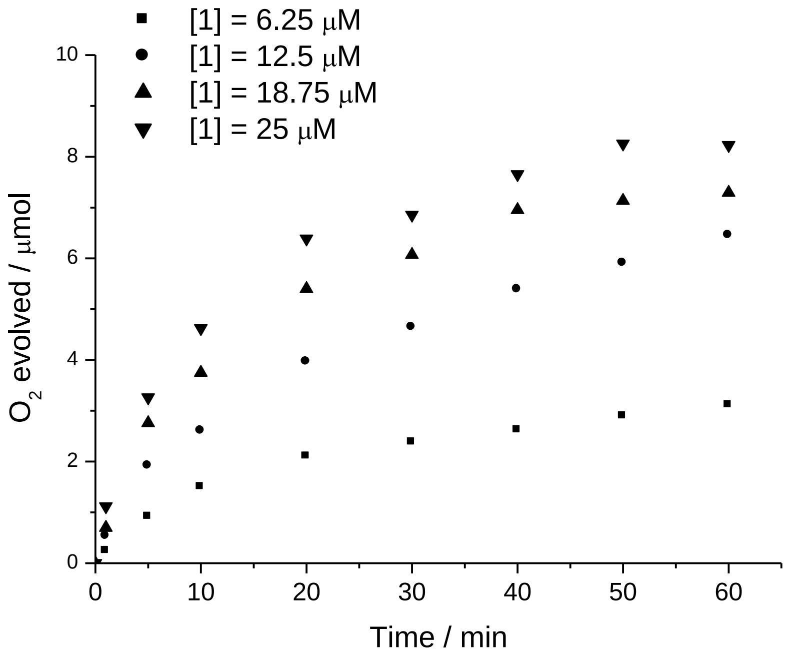

Fig. S2 Plot of O_2 evolution in **1**-catalysed water oxidation with CAN in 0.1 M HNO_3 with time for different concentrations of **1**. Concentration of CAN: 125 mM.

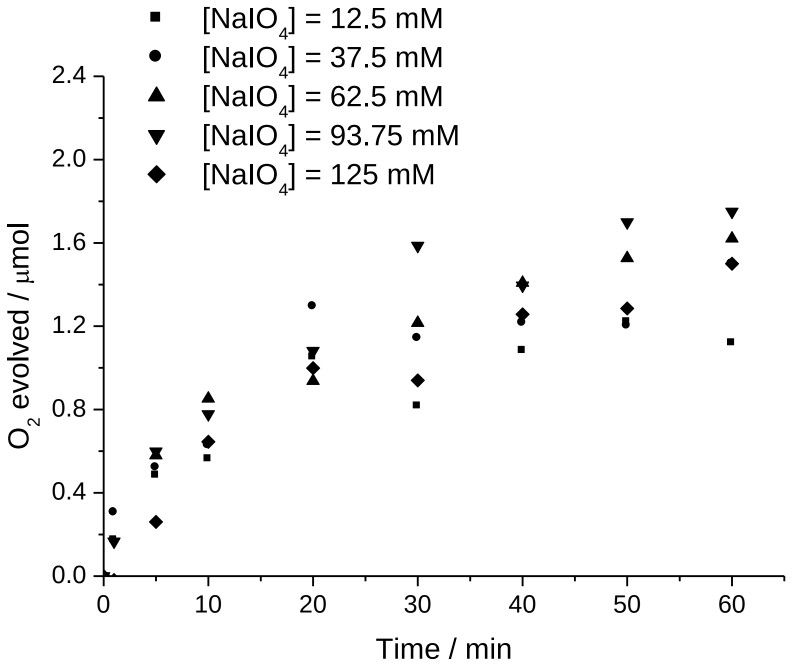

Fig. S3 Plot of O_2 evolution in **1**-catalysed water oxidation with $NaIO_4$ in 0.1 M HNO_3 with time for different concentrations of $NaIO_4$. Concentration of **1**: 12.5 μM.

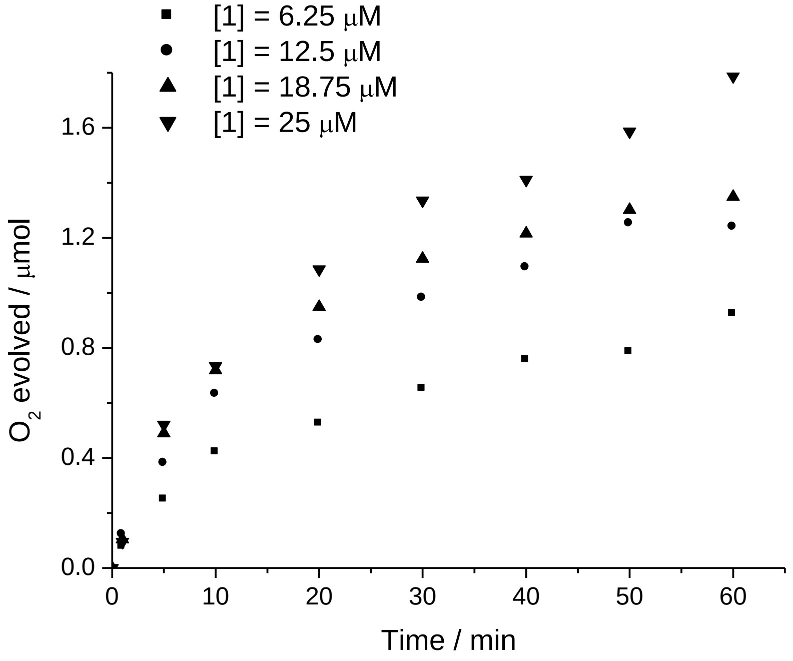

Fig. S4 Plot of O_2 evolution in **1**-catalysed water oxidation with $NaIO_4$ in 0.1 M HNO_3 with time for different concentrations of **1**. Concentration of $NaIO_4$: 125 mM.

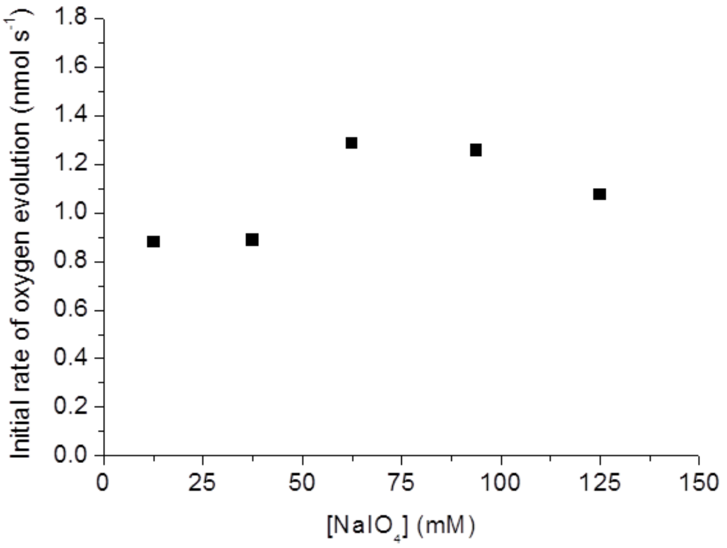

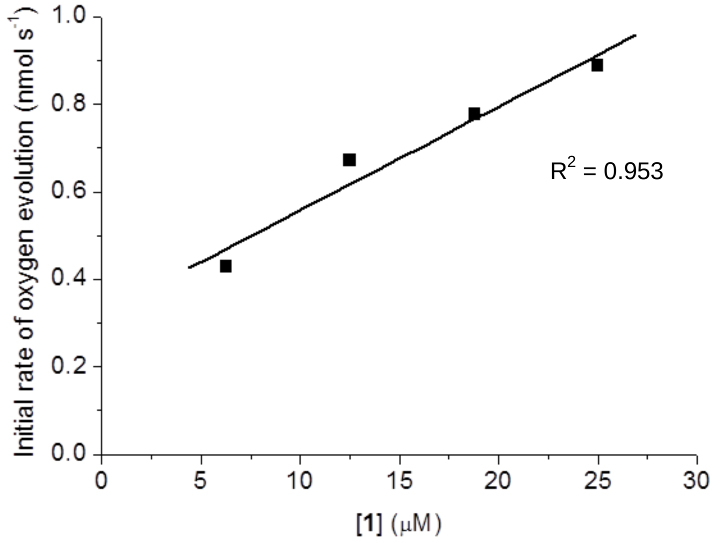

Fig. S5 Upper: Plot of initial rate of O_2 evolution against different [$NaIO_4$] (12.5–125 mM) at fixed [**1**] (12.5 μM) in 0.1 M HNO_3. Lower: Plot of initial rate of O_2 evolution against different [**1**] (6.25–25.0 μM) at fixed [$NaIO_4$] (125 mM) in 0.1 M HNO_3.

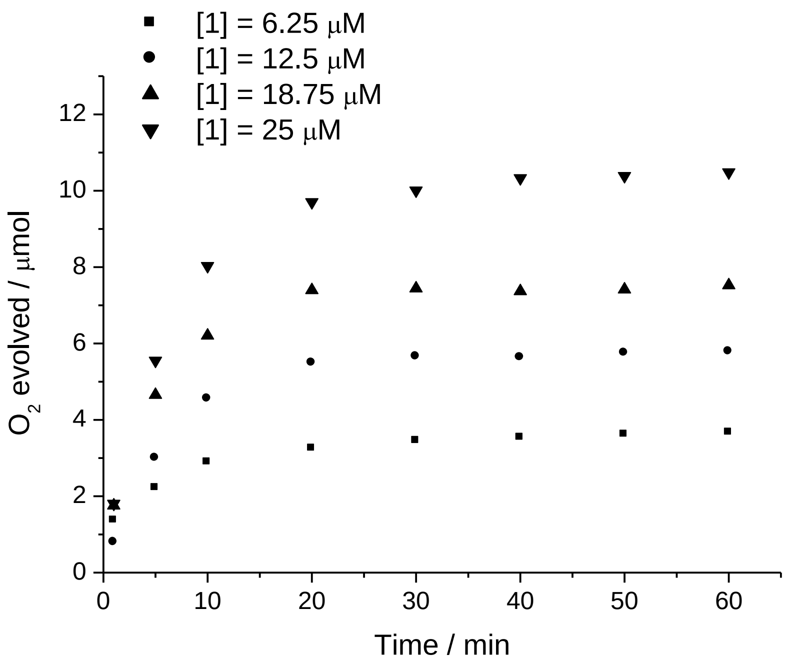

Fig. S6 Plot of O_2 evolution in **1**-catalysed water oxidation with Oxone in 0.1 M HNO_3 with time for different concentrations of **1**. Concentration of Oxone: 125 mM.

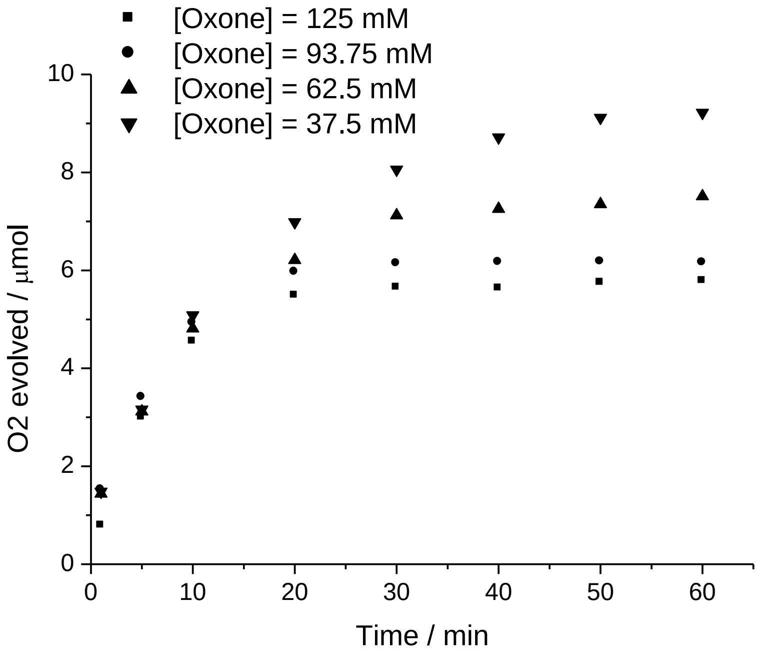

Fig. S7 Plot of O_2 evolution in **1**-catalysed water oxidation with Oxone in 0.1 M HNO_3 with time for different concentrations of Oxone. Concentration of **1**: 12.5 μM.

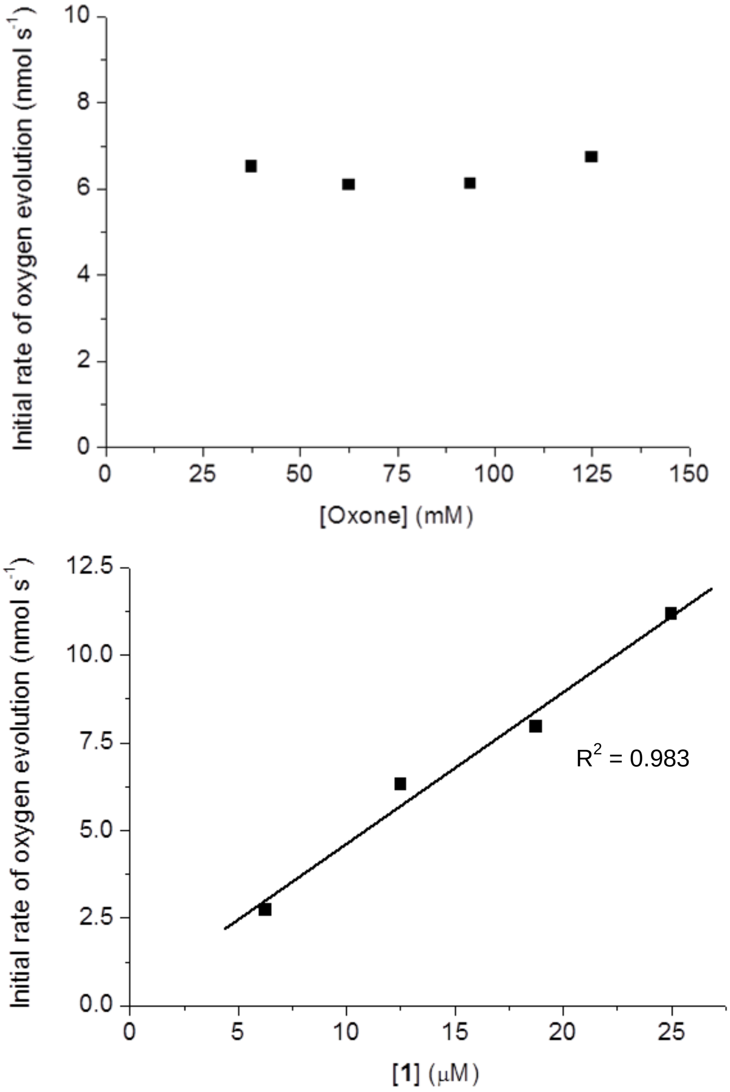

Fig. S8 Upper: Plot of initial rate of O_2 evolution against different [Oxone] (37.5–125 mM) at fixed [**1**] (12.5 μM) in 0.1 M HNO_3. Lower: Plot of initial rate of O_2 evolution against different [**1**] (6.25–25.0 μM) at fixed [Oxone] (125 mM) in 0.1 M HNO_3.

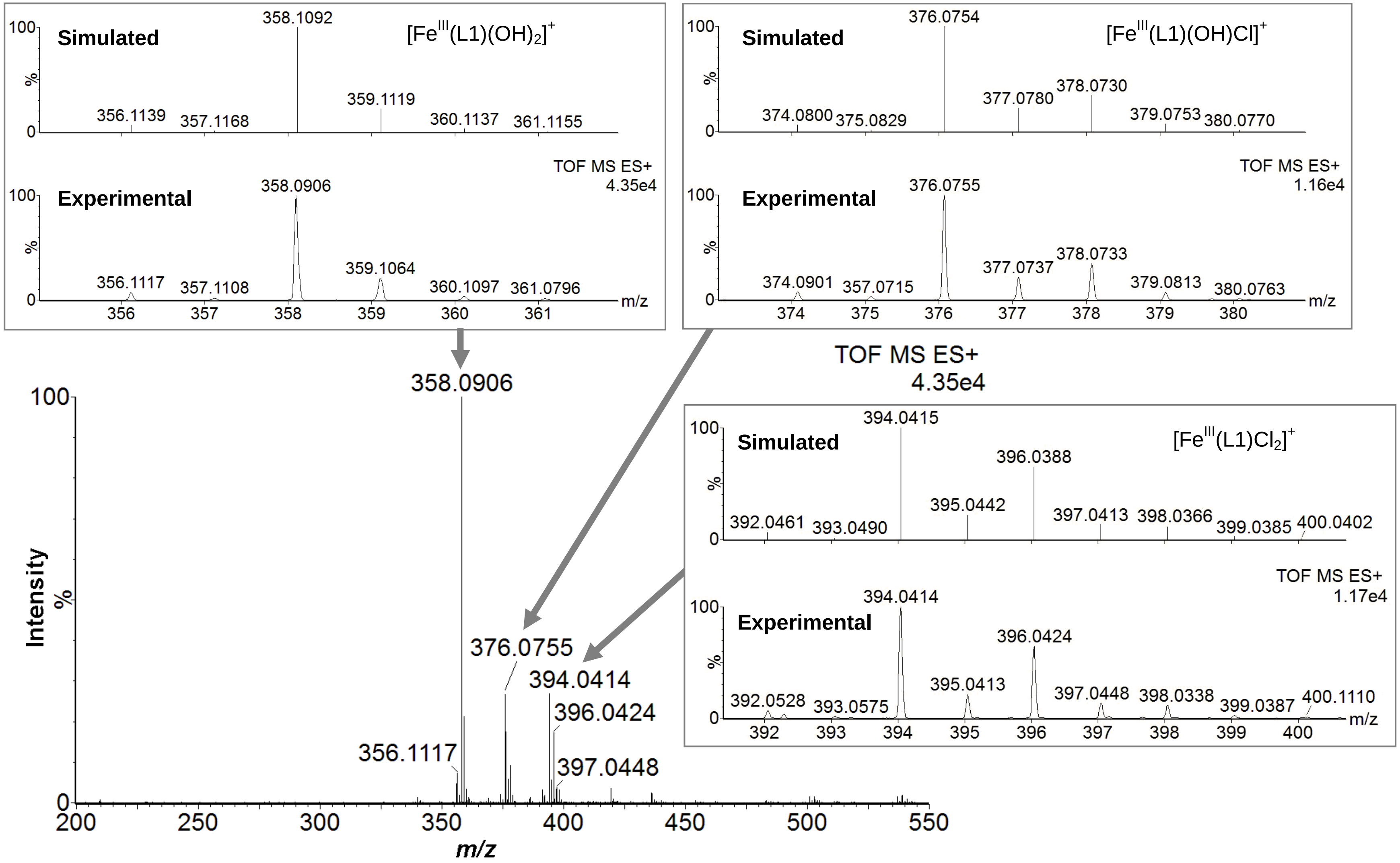

Fig. S9 ESI mass spectrum of **1**·$FeCl_4$ in H_2O.

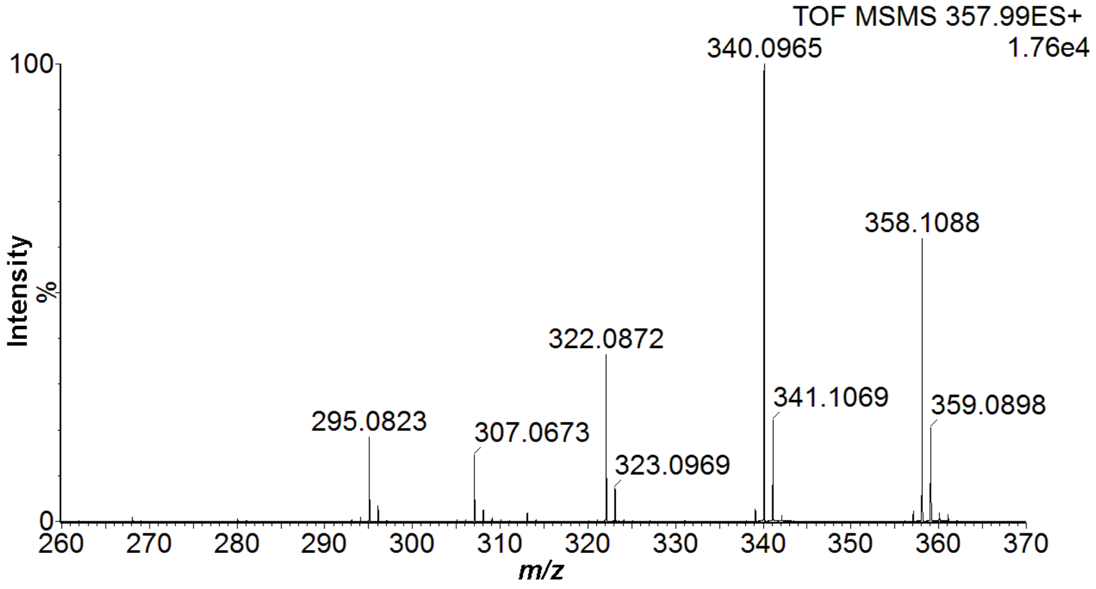

Fig. S10 Collision-induced dissociation of $[Fe^{III}(L1)(OH)_2]^+$ generated in the solution of **1** in H_2O recorded at the collision energy of 15 eV.

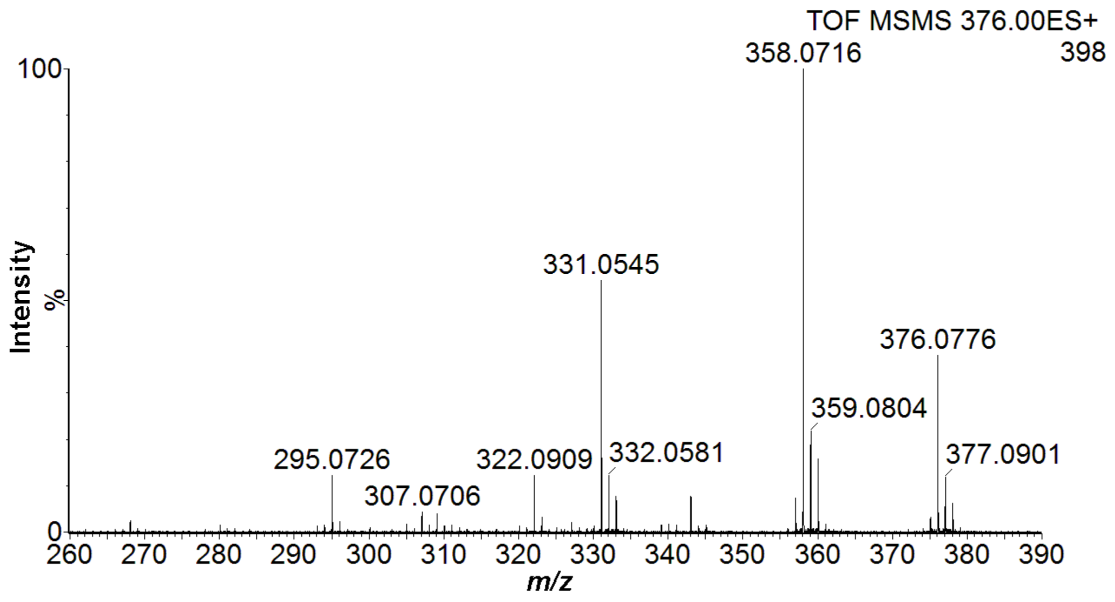

Fig. S11 Collision-induced dissociation of $[Fe^{III}(L1)(OH)Cl]^+$ generated in the solution of **1** in H_2O recorded at the collision energy of 20 eV.

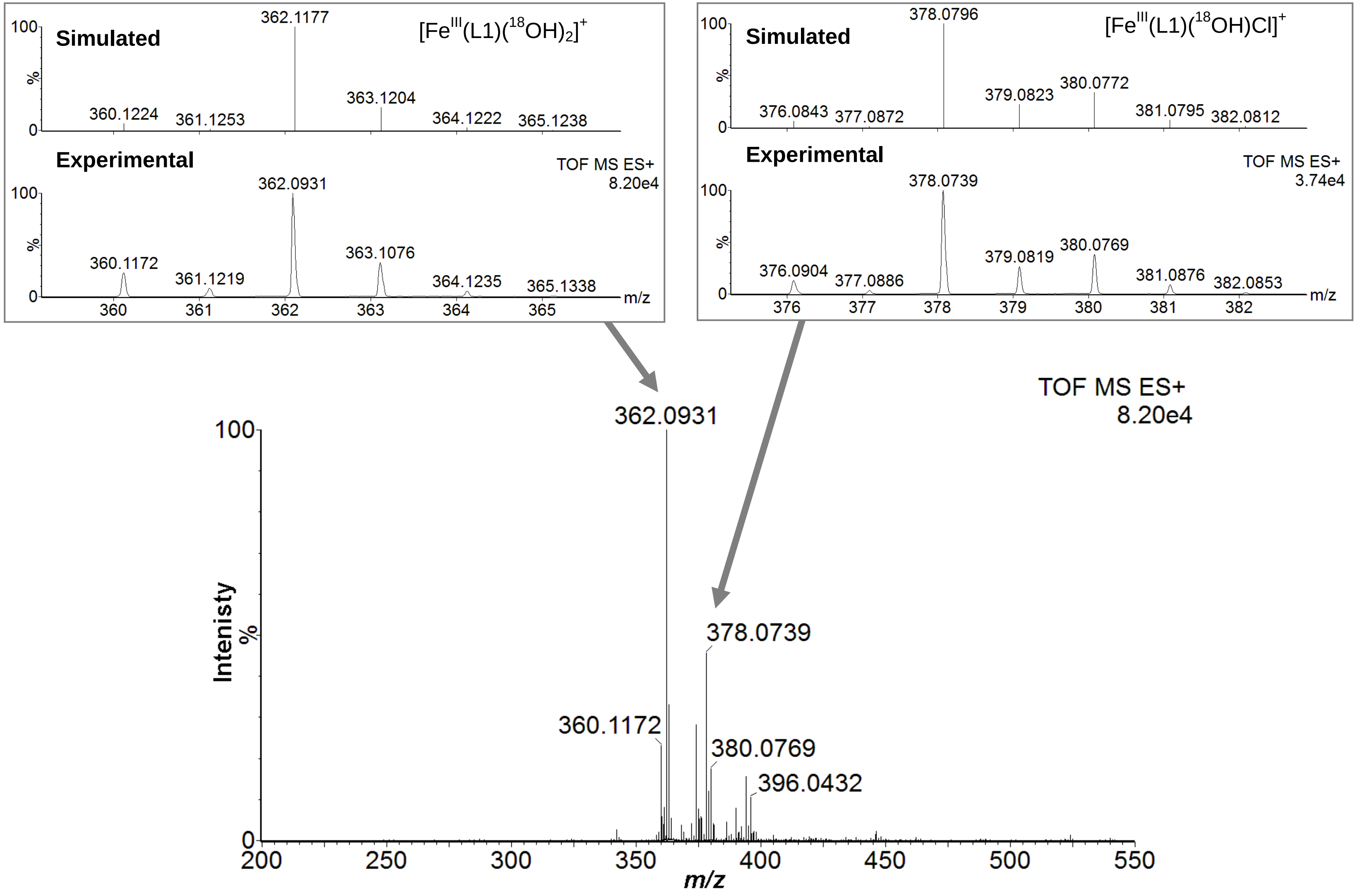

Fig. S12 ESI mass spectrum of **1** in $H_2{}^{18}O$.

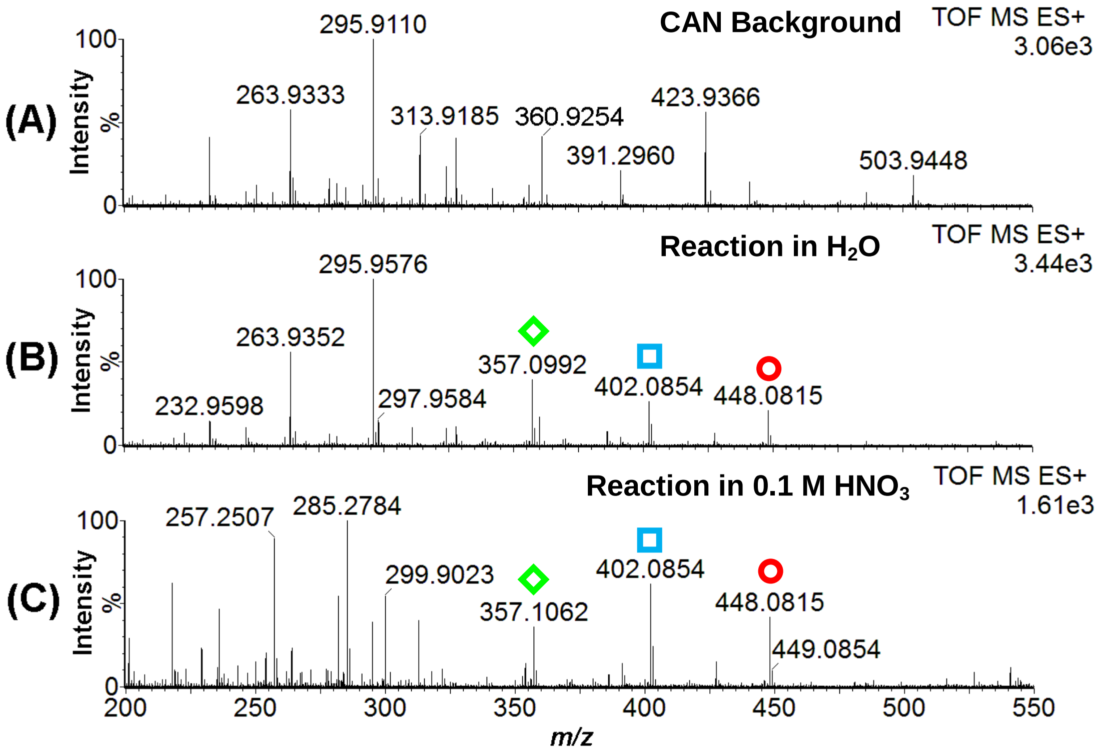

Fig. S13 ESI-MS analysis of the reaction mixture of **1** with CAN in H_2O and in 0.1 M HNO_3: (A) CAN in H_2O only, (B) Reaction of **1** with CAN (200 equiv) in H_2O and (C) Reaction of **1** with CAN (200 equiv) in 0.1 M HNO_3.

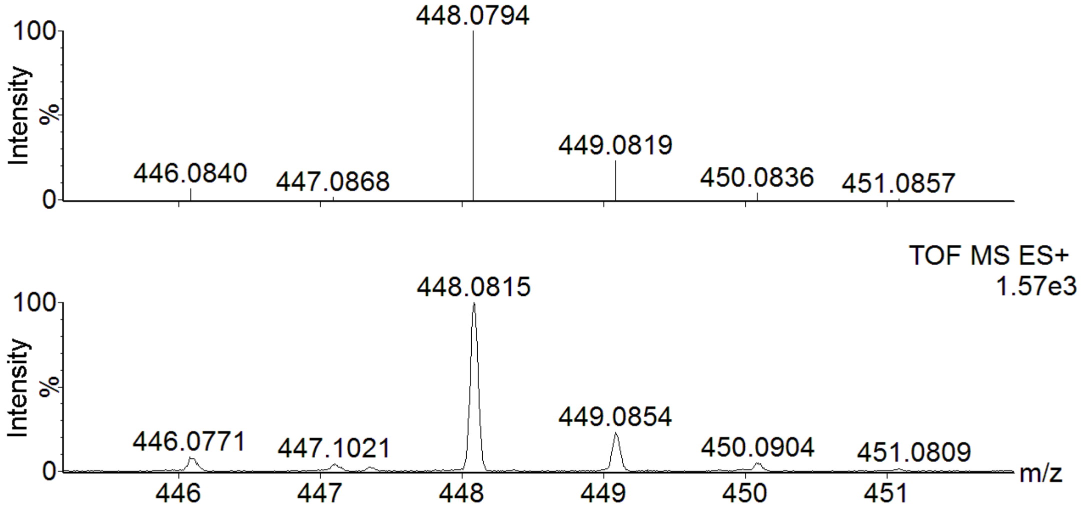

Fig. S14 Upper: Simulated isotopic pattern for $[Fe^{III}(L1)(NO_3)_2]^+$. Lower: Experimental isotopic pattern for the cluster peak at *m*/*z* 448.0815 observed by ESI-MS analysis of the reaction mixture of **1** with CAN (200 equiv) in 0.1 M HNO_3.

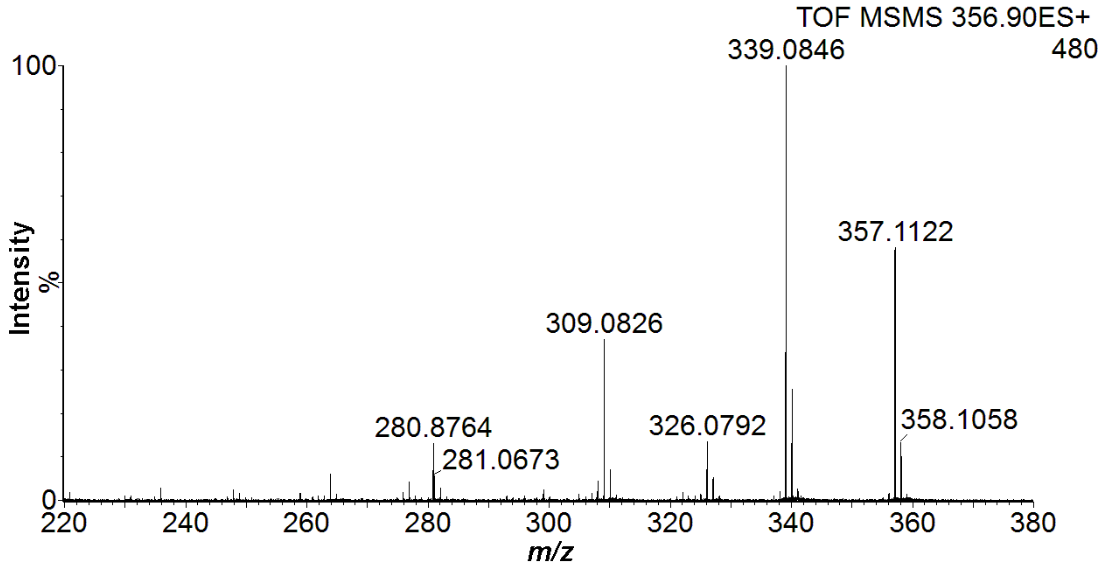

Fig. S15 Collision-induced dissociation of the ion with *m/z* ~357.1 (for the reaction of **1** with CAN (200 equiv) in 0.1 M HNO_3) recorded at the collision energy of 15 eV.

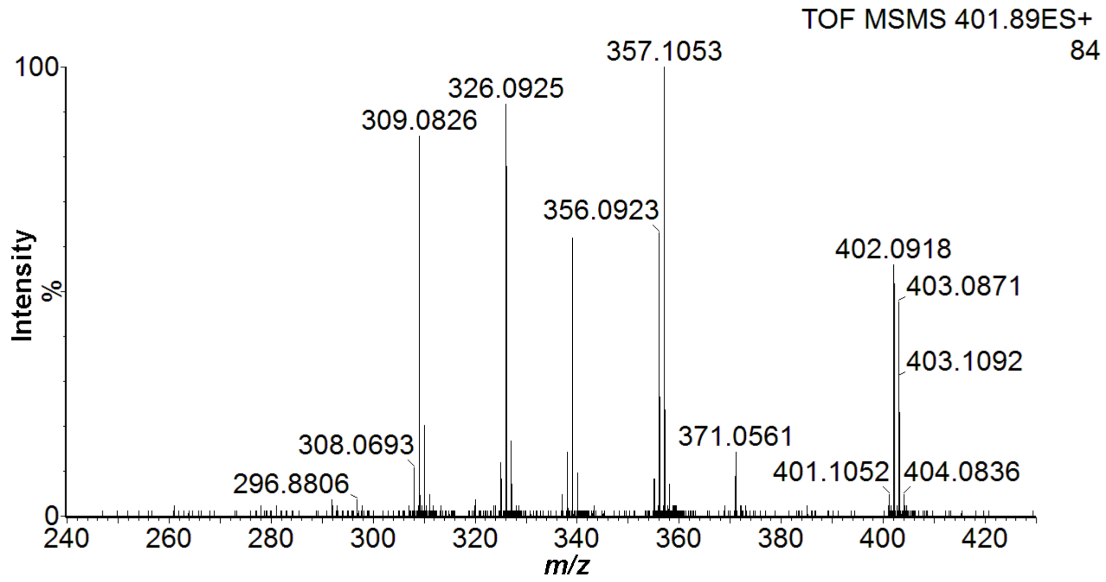

Fig. S16 Collision-induced dissociation of the ion at *m/z* ~402.1 (for the reaction of **1** with CAN (200 equiv) in 0.1 M HNO_3) recorded at the collision energy of 12 eV.

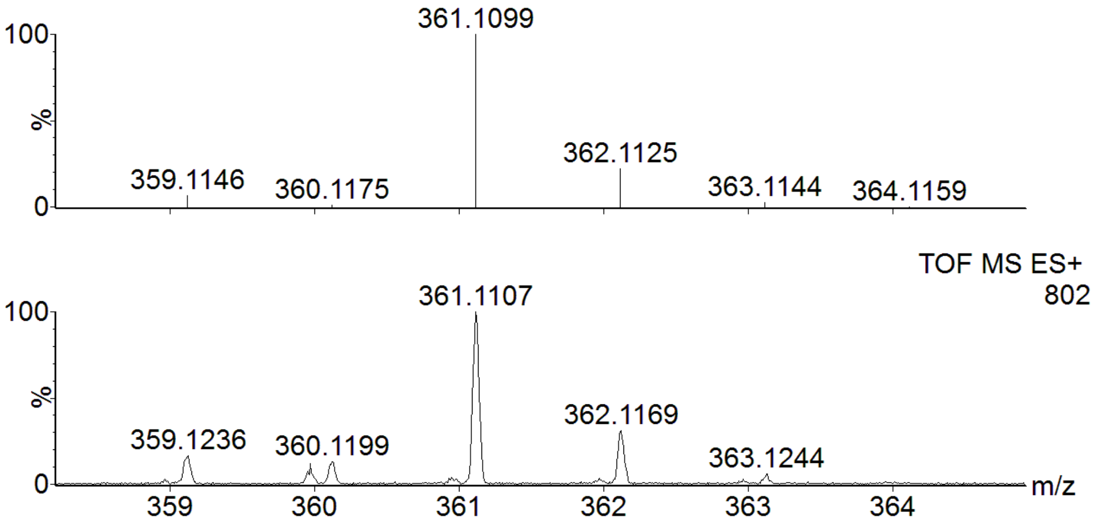

Fig. S17 Upper: Simulated isotopic pattern for $[Fe^{IV}(L1)(^{18}O)(^{18}OH)]^{+}$. Lower: Experimental isotopic pattern for the cluster peak at *m/z* 361.1107 observed by ESI-MS analysis of the reaction mixture of **1** with CAN (200 equiv) in $H_2{}^{18}O$.

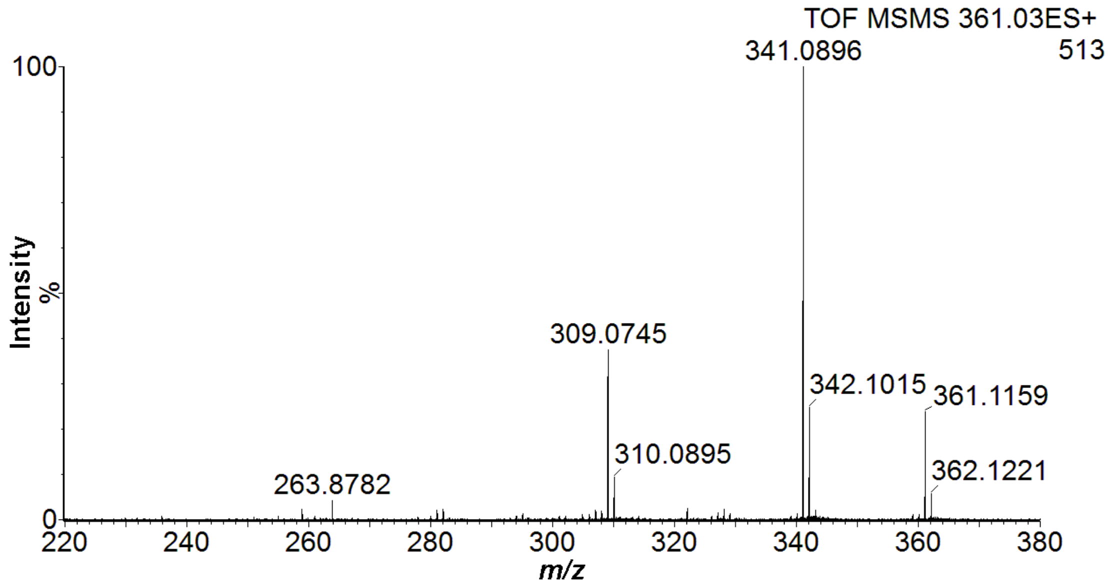

Fig. S18 Collision-induced dissociation of the ion with *m*/*z* ~361.1 (for the reaction of **1** with CAN in $H_2{}^{18}O$) recorded at the collision energy of 15 eV.

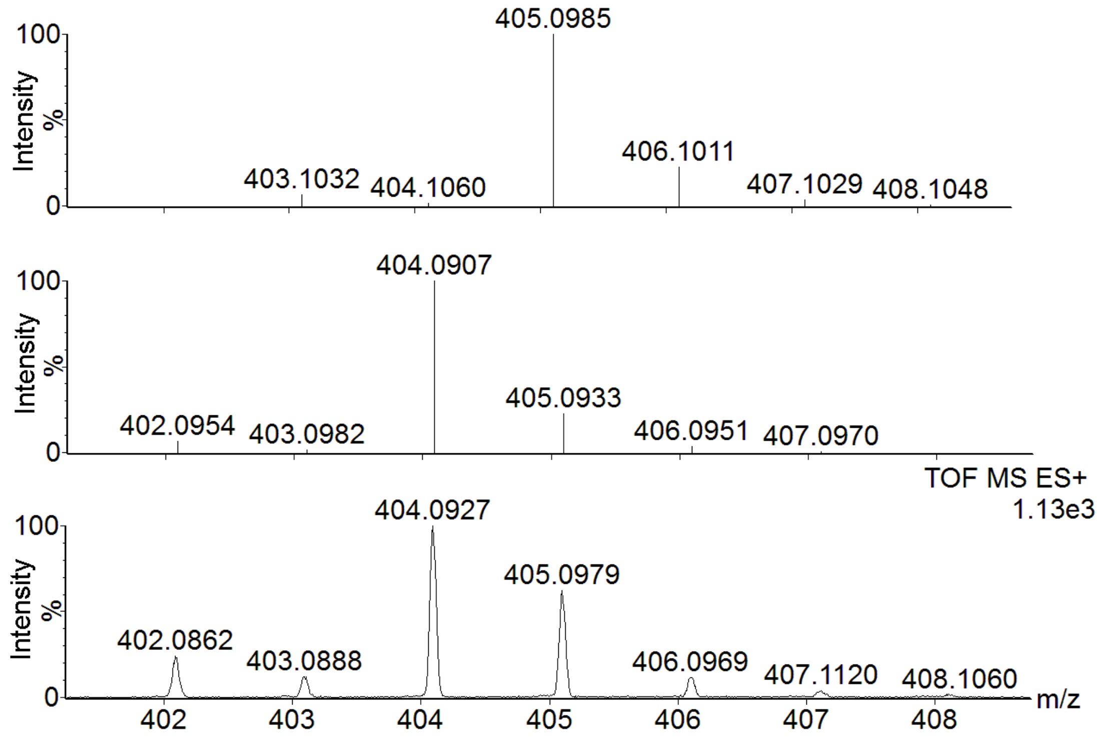

Fig. S19 ESI-MS measurement of species at *m/z* 404.0927 in reaction of **1** with CAN (200 equiv) in $H_2{}^{18}O$. Upper: Simulated isotopic pattern for $[Fe^{III}(L1)(^{18}OH)(N^{16}O_3)]^+$. Middle: Simulated isotopic pattern for $[Fe^{IV}(L1)(^{18}O)(N^{16}O_3)]^+$. Bottom: Experimental isotopic pattern.

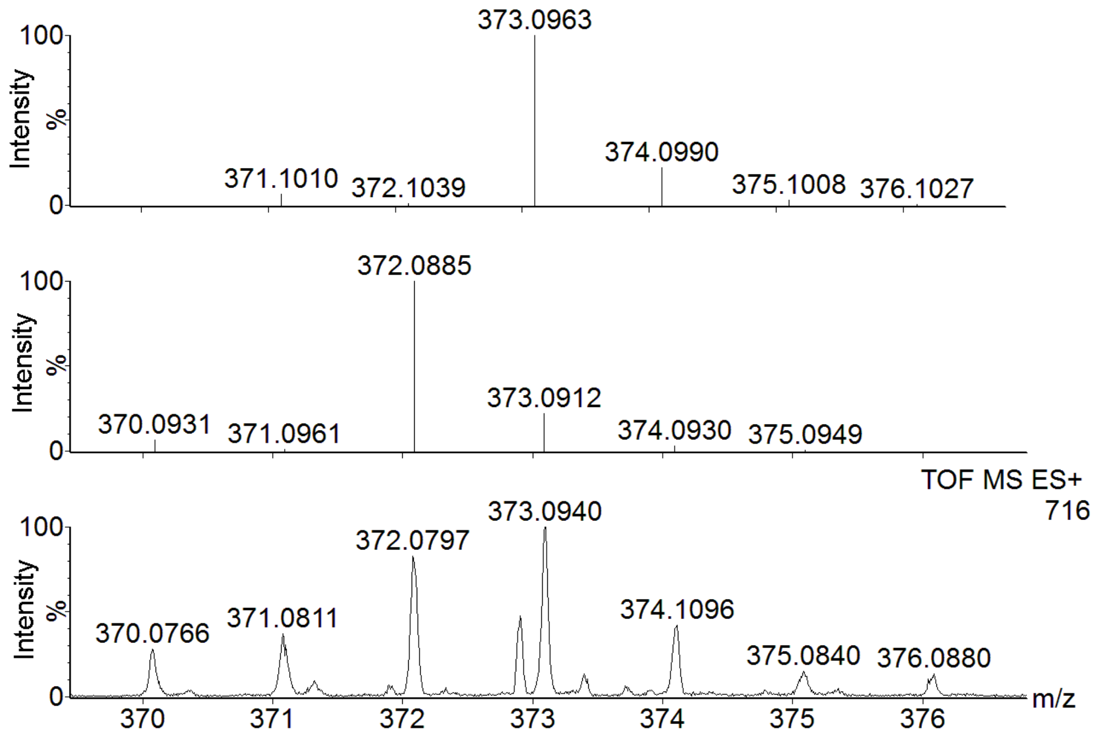

Fig. S20 Electrospray ionization mass measurement of species at *m/z* 373.0940 in reaction of **1** with $NaIO_4$ (800 equiv) in 0.1 M HNO_3. Upper: Simulated isotopic pattern for $[Fe^{III}(L1)(OO^{\bullet})(OH)]^+$. Middle: Simulated isotopic pattern for $[Fe^{IV}(L1)(OO^{\bullet})(O)]^+$. Bottom: Experimental isotopic pattern.

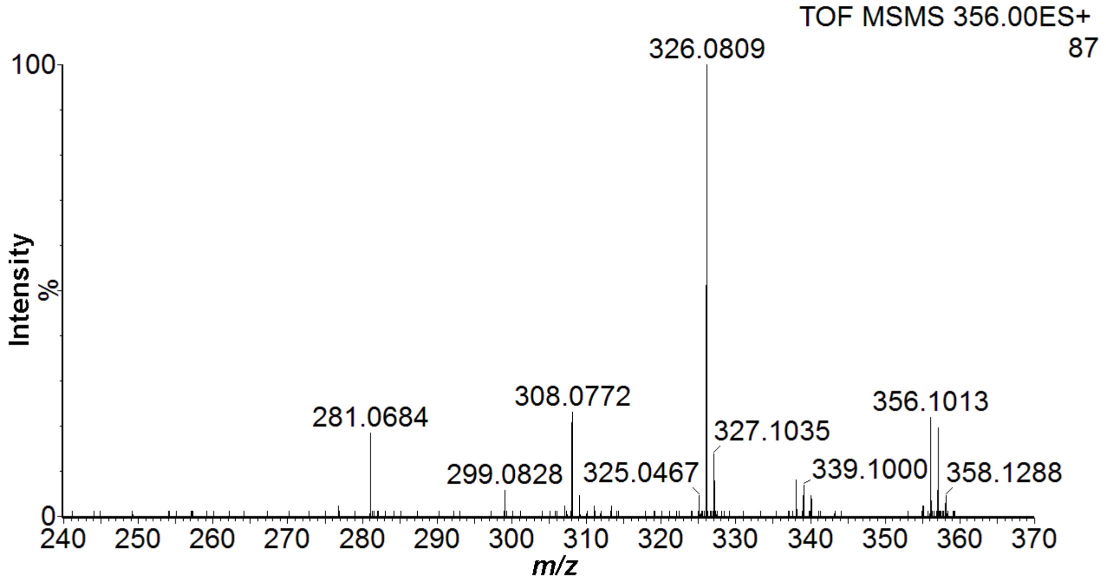

Fig. S21 Collision-induced dissociation of the ion with *m/z* ~356.1 (for the reaction of **1** with $NaIO_4$ in 0.1 M HNO_3) recorded at the collision energy of 12 eV.

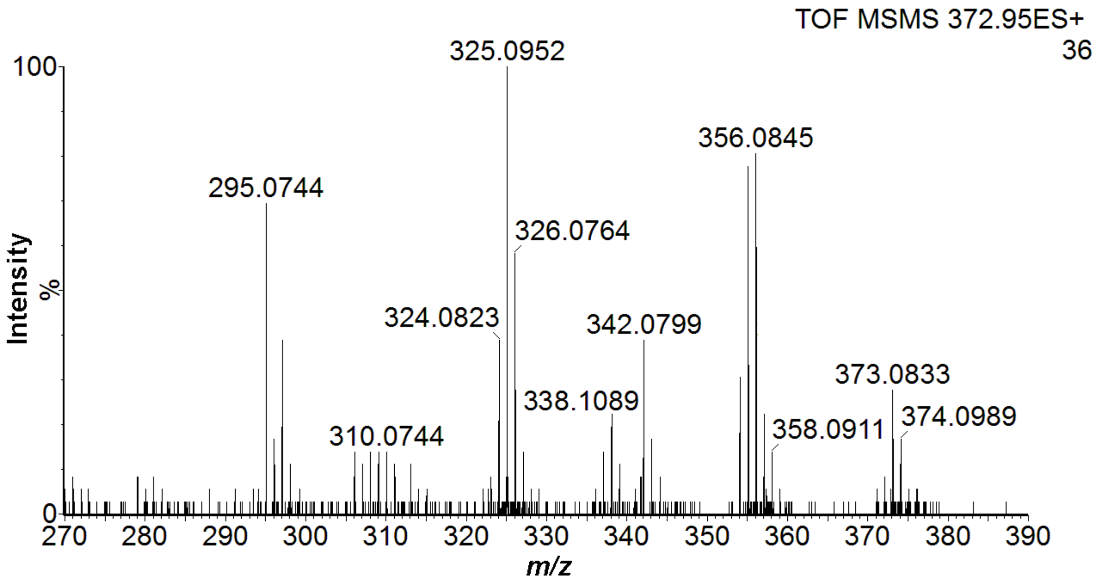

Fig. S22 Collision-induced dissociation of the ion with *m/z* ~373.1 (for the reaction of **1** with $NaIO_4$ in 0.1 M HNO_3) recorded at the collision energy of 15 eV.

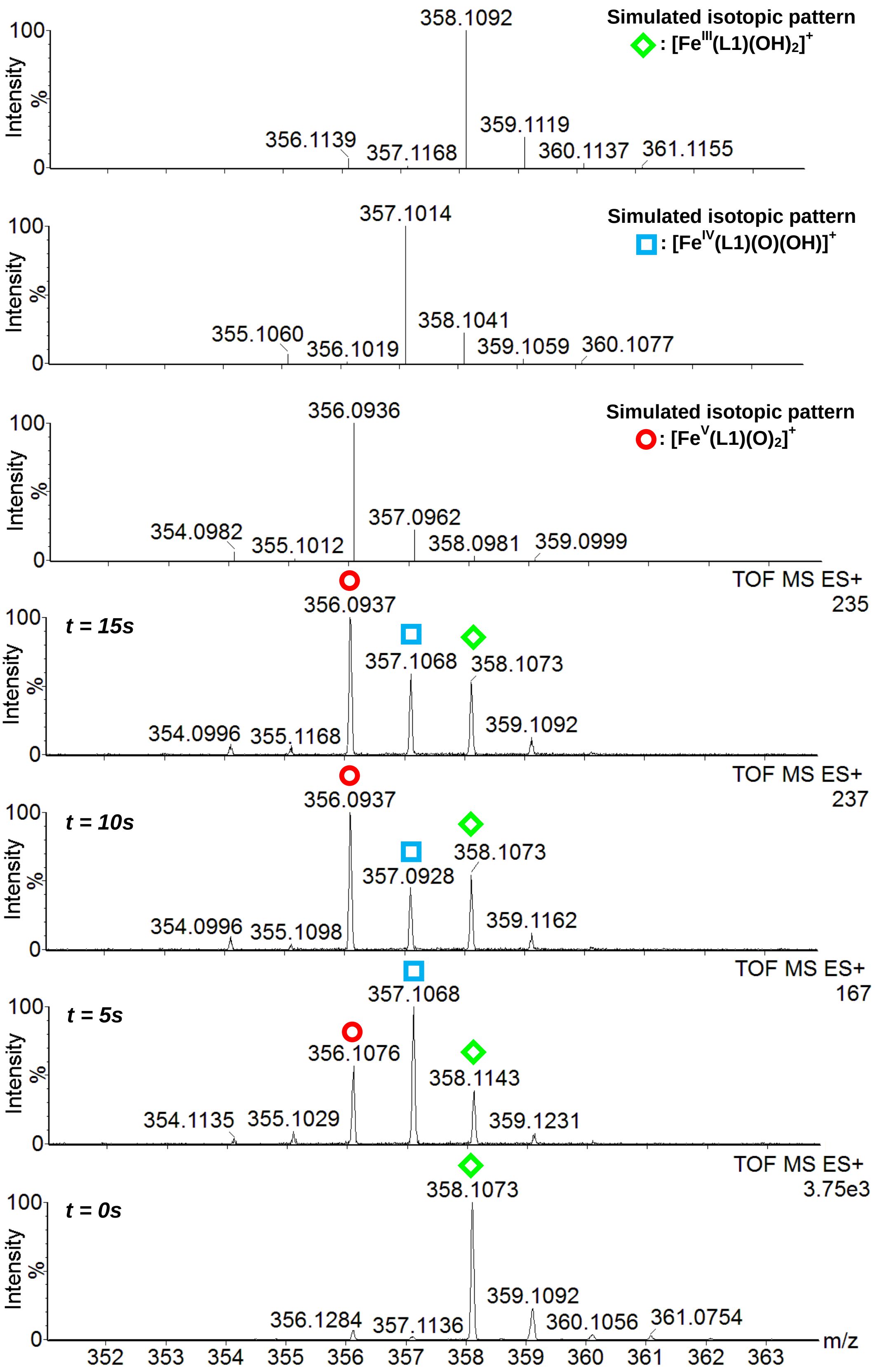

Fig. S23 ESI-MS measurements of the reaction mixture of **1** with $NaIO_4$ (800 equiv) in H_2O at different reaction time (*t*): 0 sec (without $NaIO_4$), 5 sec, 10 sec and 15 sec.

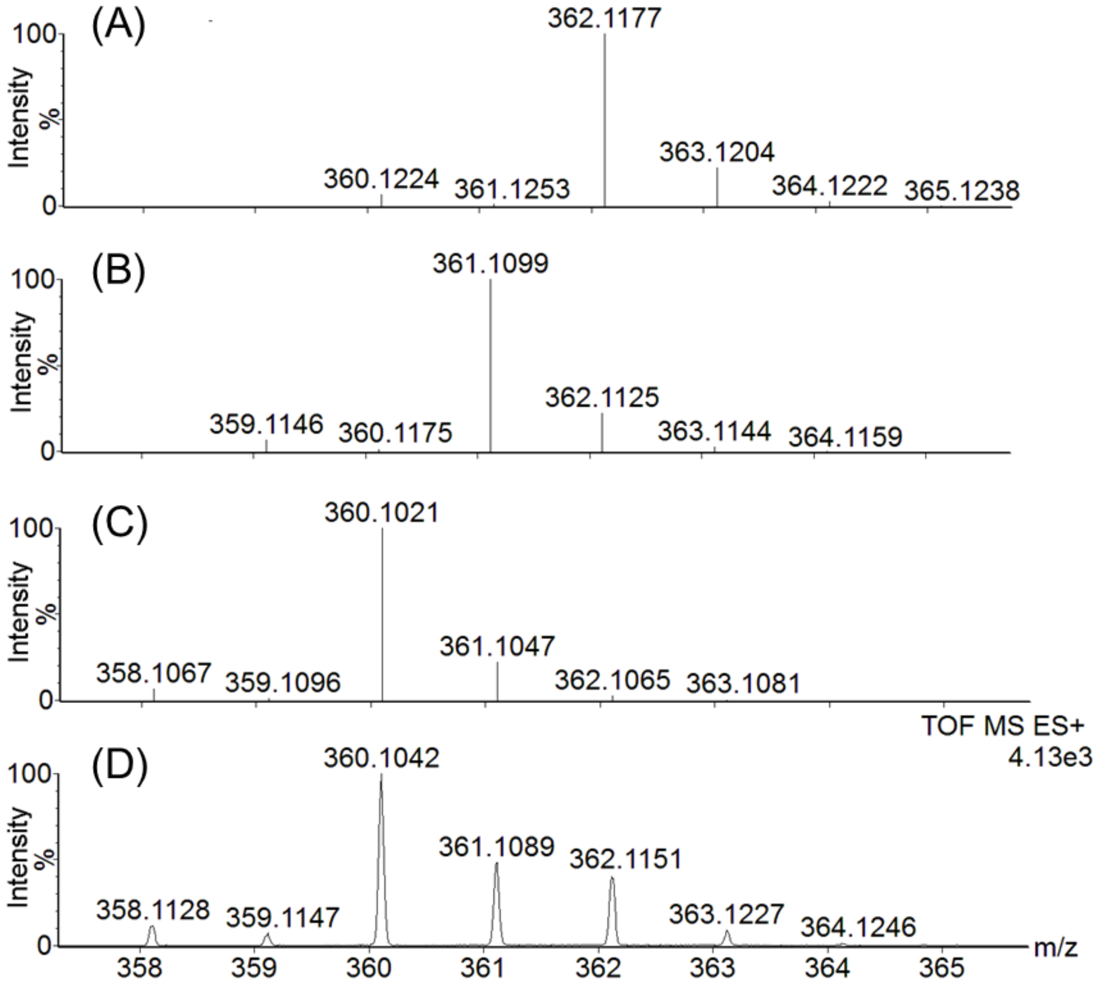

Fig. S24 ESI-MS measurement of the species at *m/z* 360.1042 in reaction of **1** with $NaIO_4$ (800 equiv) in $H_2{}^{18}O$. (A) Simulated isotopic pattern for $[Fe^{III}(L1)(^{18}OH)_2]^+$. (B) Simulated isotopic pattern for $[Fe^{IV}(L1)(^{18}O)(^{18}OH)]^+$. (C) Simulated isotopic pattern for $[Fe^{V}(L1)(^{18}O)_2]^+$. (D) Experimental isotopic pattern.

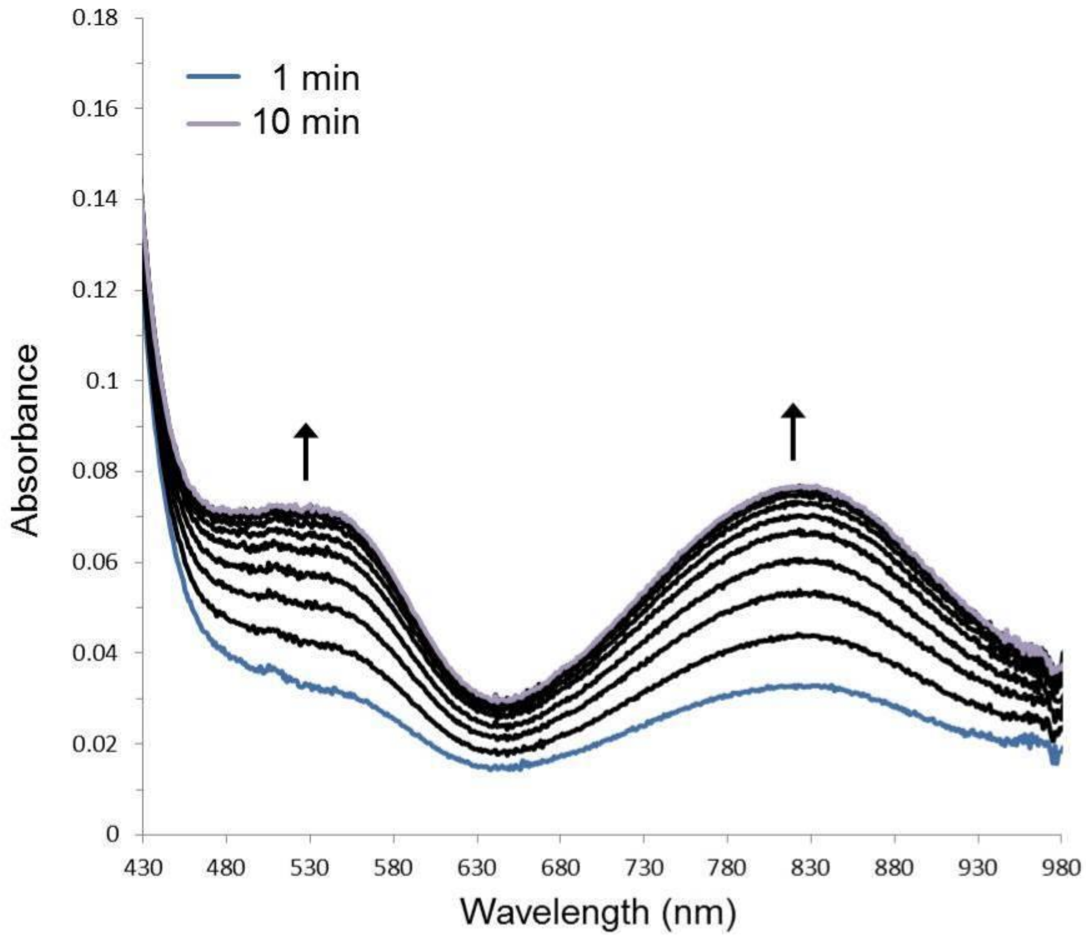

Fig. S25 UV-vis absorption spectral changes for the reaction of **1** (1.5 mM) with $NaIO_4$ (7.5 mM) in 0.1 M HNO_3 at room temperature at reaction times of 1–10 min.

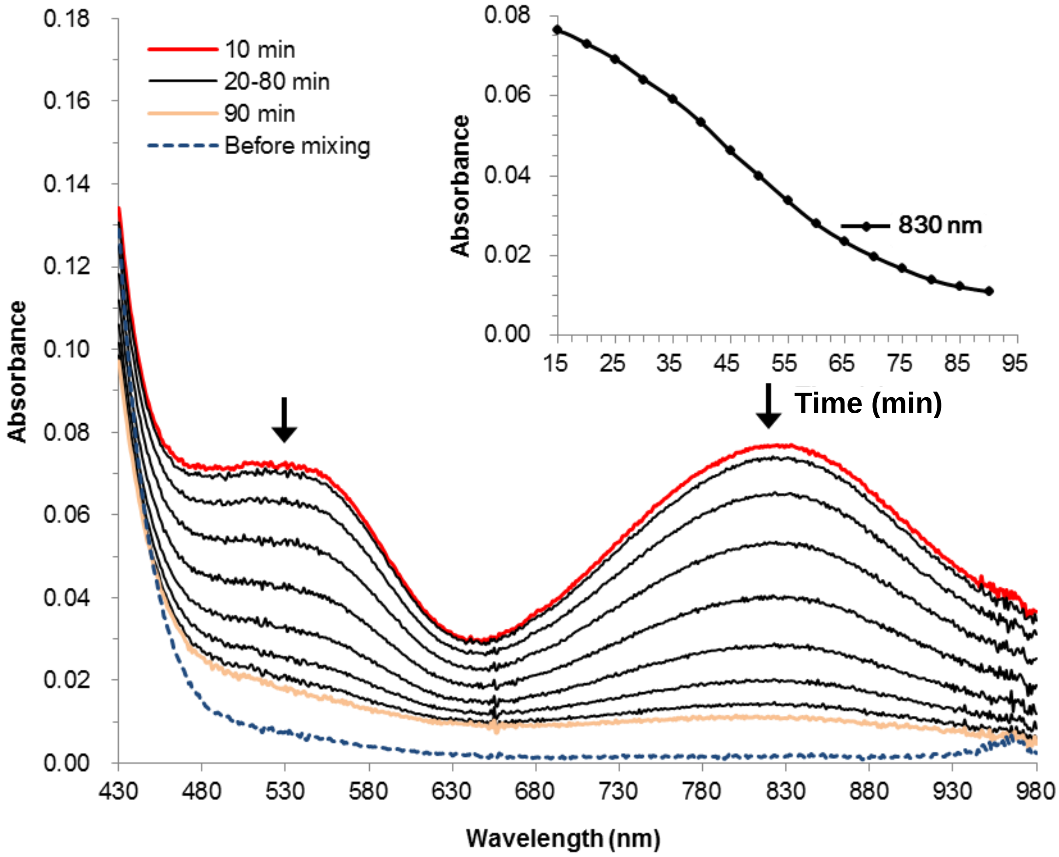

Fig. S26 UV-vis absorption spectral changes for the reaction of **1** (1.5 mM) with $NaIO_4$ (7.5 mM) in 0.1 M HNO_3 at room temperature at reaction times of 10–90 min. Inset: Time course of the decay of absorption band monitored at 830 nm.

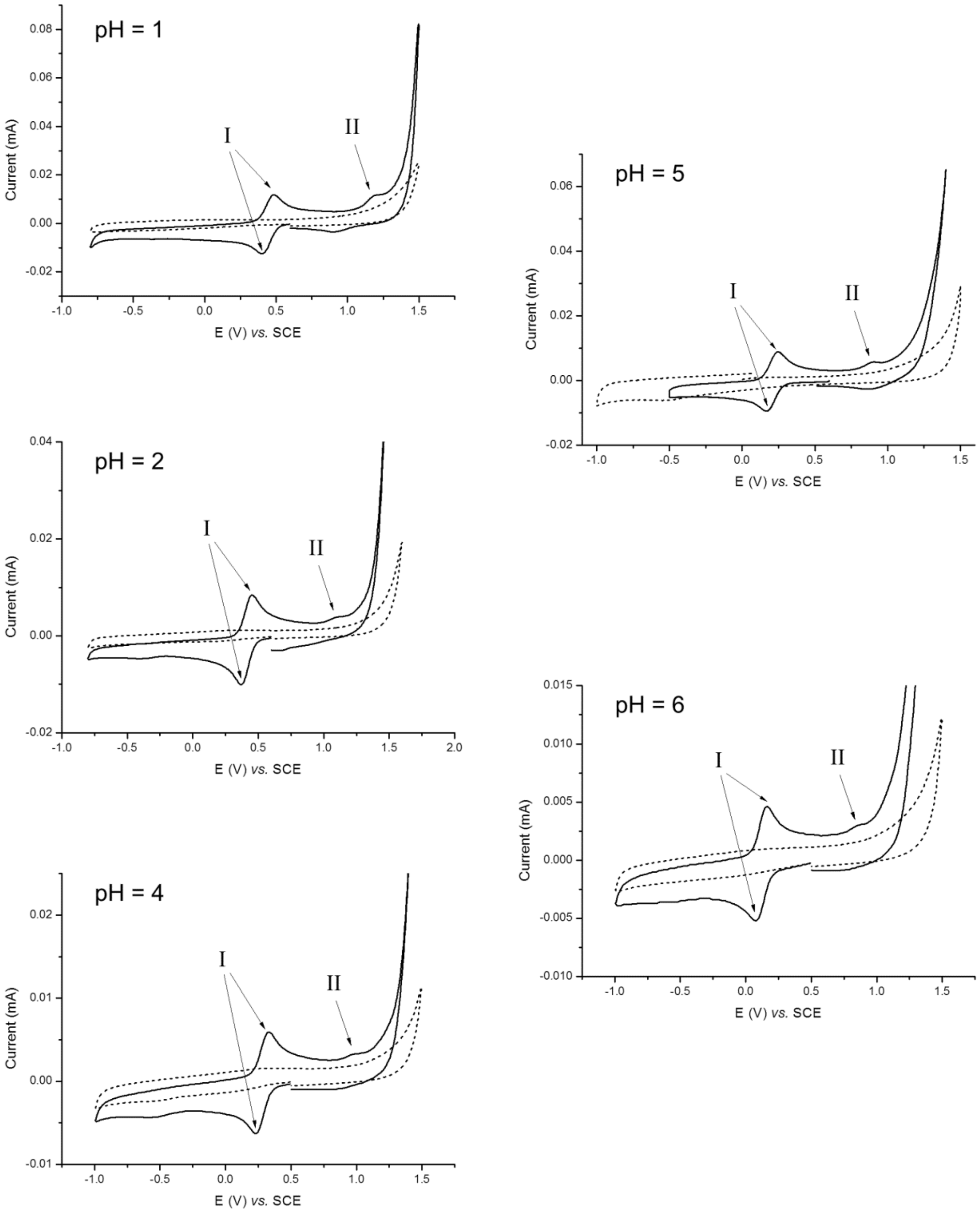

Fig. S27 Solid lines: cyclic voltammograms of **1**·ClO_4 in 0.1 M HNO_3 at pH 1, in 0.1 M CF_3SO_3H/CF_3SO_3Li at pH 2, and in 0.1 M acetate buffer at pH 4–6. Dotted lines: cyclic voltammograms of the acid/buffer background.

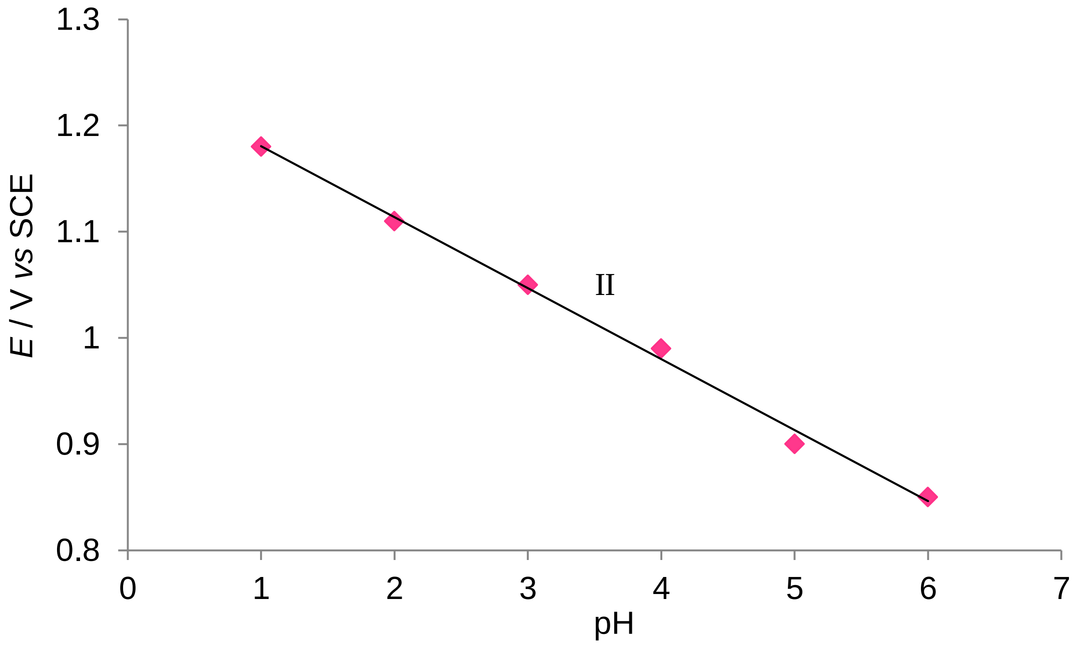

Fig. S28 Redox potentials (the irreversible wave II) of **1**·ClO_4 in solutions at various pH.

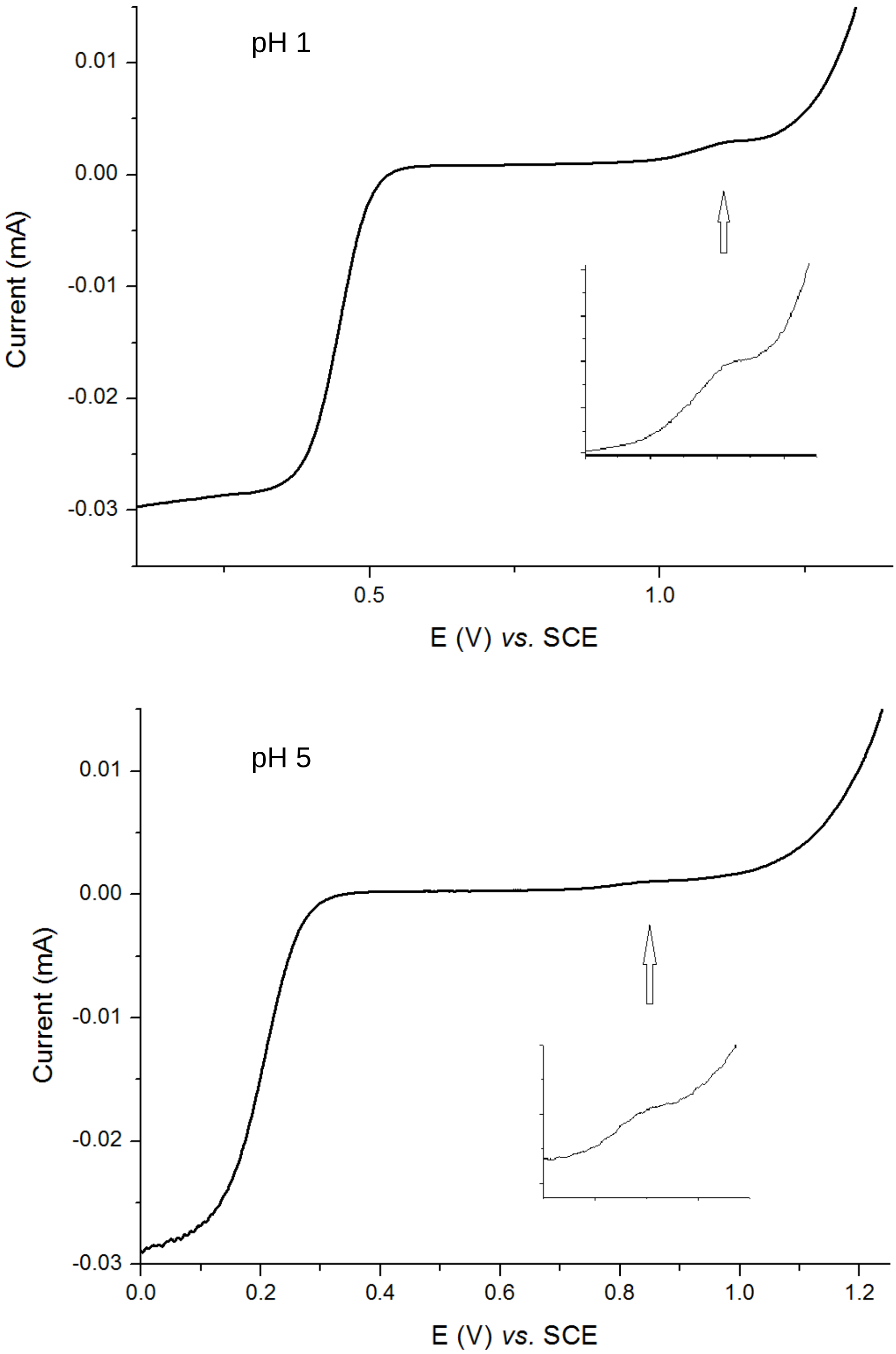

Fig. S29 Linear scan voltammograms of **1**·ClO_4 in 0.1 M HNO_3 at pH 1 (rotation rate: 100 rpm; inset: magnified region at approximately 1.1 V) and at pH 5 (rotation rate: 400 rpm; inset; magnified region at approximately 0.9 V). Working electrode: rotating glassy carbon disk; scan rate: 5 mV/s.

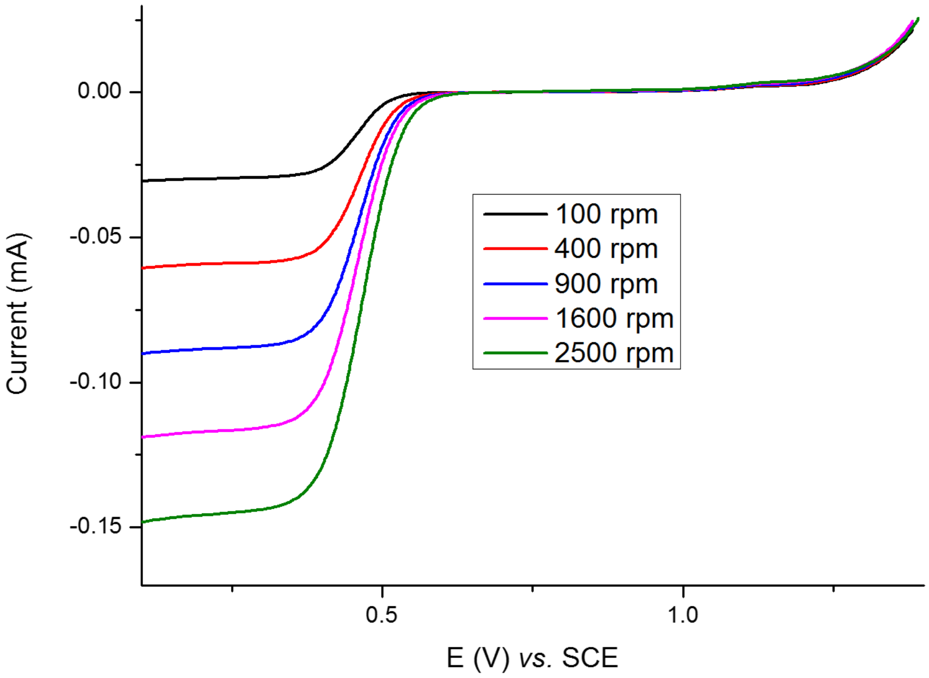

Fig. S30 Linear scan voltammogram of **1**·ClO_4 in 0.1 M HNO_3 (pH 1) at various rotation rates. Working electrode: rotating glassy carbon disk; rotation rate: 100 to 2500 rpm (as denoted in the voltammogram); scan rate: 5 mV/s.

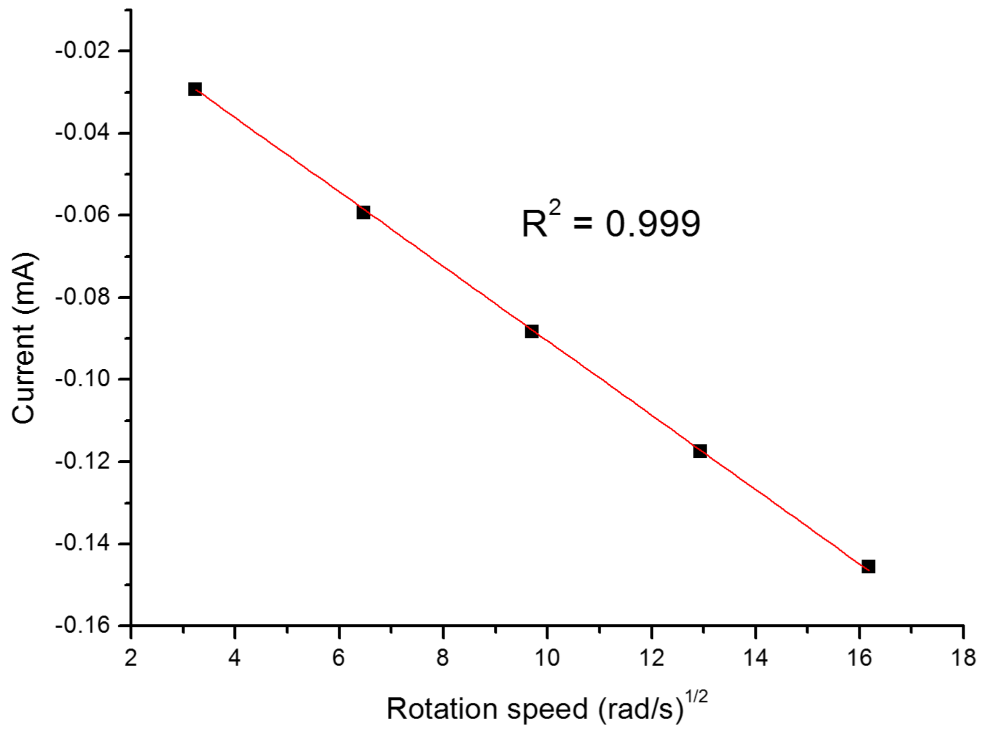

Fig. S31 Levich plot for the limiting current of the redox process corresponding to the reversible couple I.

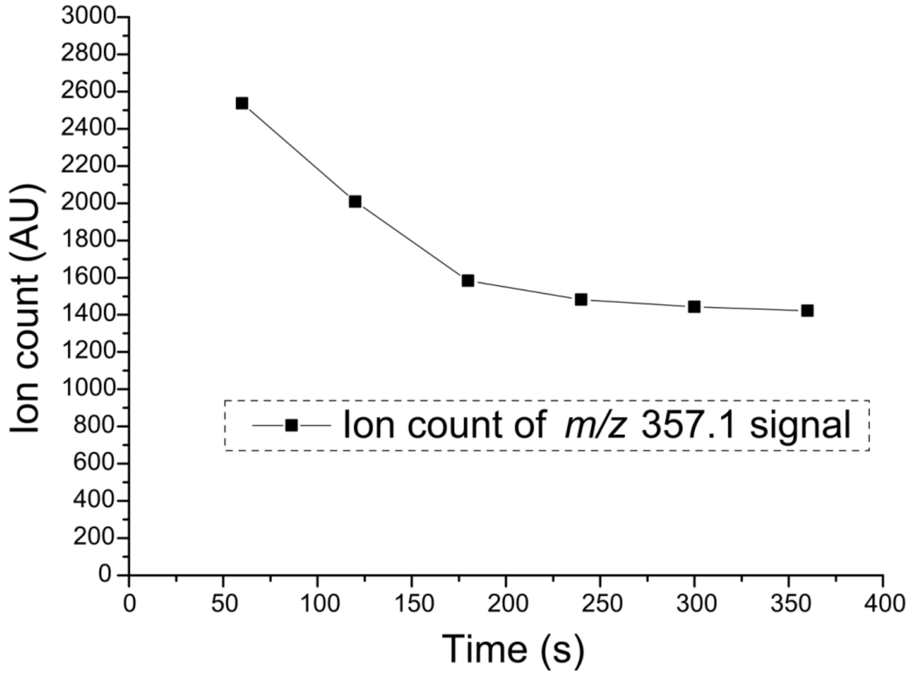

Fig. S32 Ion counts of the ESI-MS signal at *m/z* 357.1 assigned to $[Fe^{IV}(L1)(O)(OH)]^+$ at different reaction times for the reaction of **1** (1.5 mM) with CAN (7.5 mM) in 0.1 M HNO_3 at room temperature.

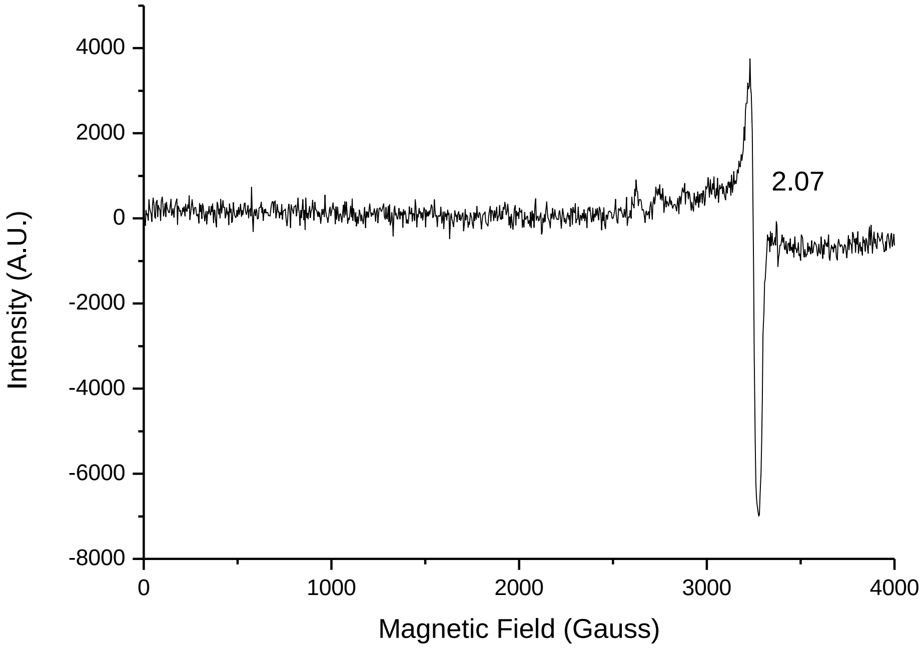

Fig. S33 X-band EPR spectrum of $NaIO_4$ (10 mM) in 0.1 M HNO_3 measured at 7 K.

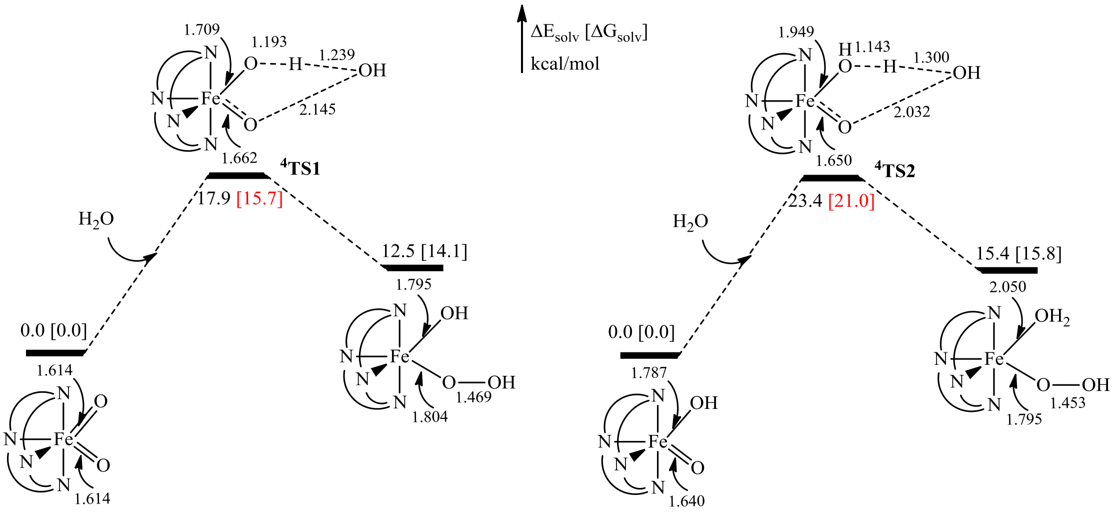

Fig. S34 Comparison of potential energy surfaces of the first step of water oxidation based on $[Fe^{V}(L1)(O)_2]^{+}$ and $[Fe^{V}(L1)(O)(OH)]^{2+}$ (free energy in kcal/mol and bond distance in Å).

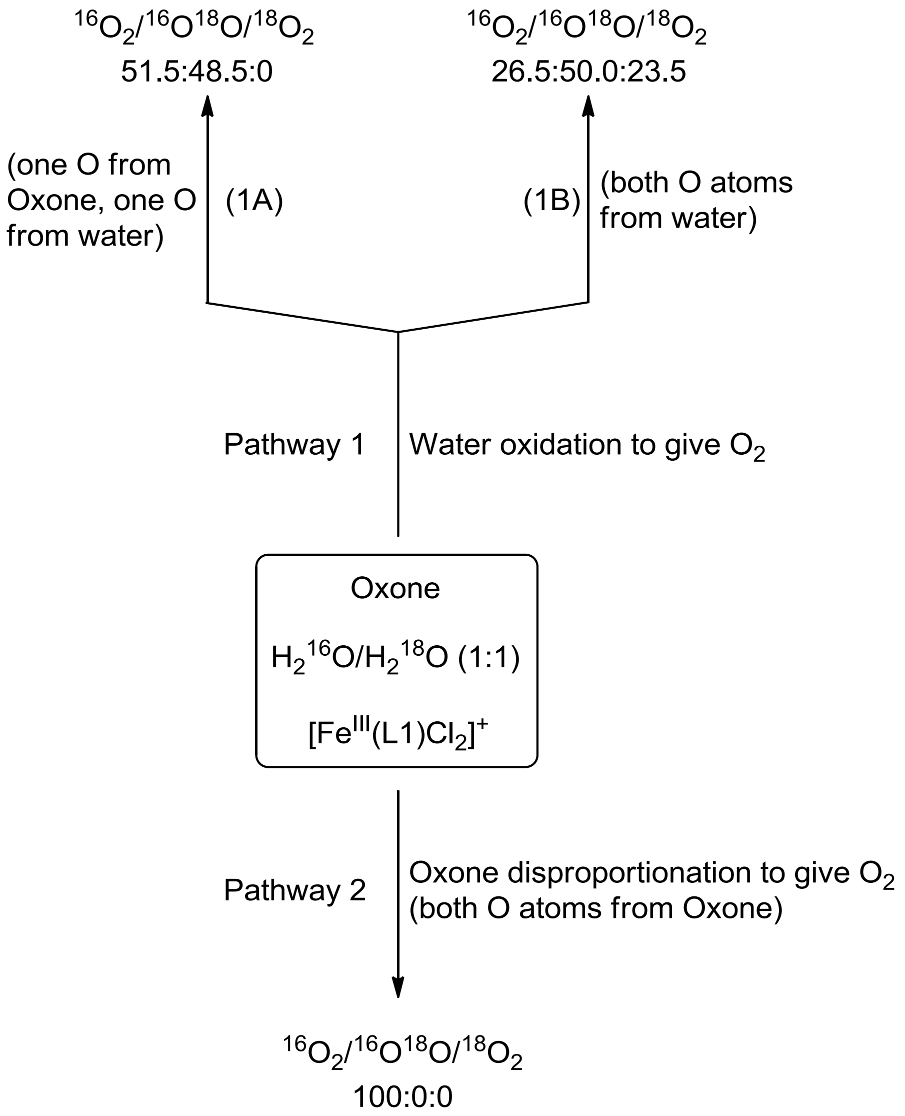

Scheme S1 Possible pathways for O_2 evolution in the reaction mixture of **1** with Oxone in $H_2{}^{16}O/H_2{}^{18}O$ (1:1, $H_2{}^{18}O$ used had 97 atom% ^{18}O). The calculated $^{16}O_2/^{16}O^{18}O/^{18}O_2$ ratios for these pathways are shown.

Cartesian coordinates for optimized structures of possible intermediates/species involved in mechanistic studies

[Ru^{V}(L2)(O)(OH)]$^{2+}$ *S* = 1/2

Center Number	Atomic Number	Atomic Type	Coordinates (Angstroms) X	Y	Z
1	44	0	-0.002973	-0.565634	0.028095
2	8	0	0.523250	-1.563448	-1.501912
3	7	0	2.155528	-0.705869	0.528297
4	7	0	0.838971	1.257758	-1.164969
5	7	0	-2.156075	-0.749704	-0.496169
6	6	0	2.547062	-2.142318	0.555288
7	6	0	2.877105	-0.017892	-0.578283
8	6	0	2.295215	1.349013	-0.811354
9	6	0	0.136220	2.493665	-0.743320
10	6	0	-2.262303	1.339856	0.798443
11	6	0	-2.861788	-0.023695	0.602211
12	6	0	-2.567182	-2.178791	-0.458550
13	6	0	0.721939	1.103380	-2.637601
14	8	0	-0.318741	-1.465460	1.452540
15	7	0	-0.806414	1.242733	1.160873
16	6	0	2.490857	-0.122926	1.844571
17	6	0	-2.523986	-0.193696	-1.818500
18	6	0	-0.101421	2.483642	0.746628
19	6	0	-0.703484	1.094024	2.638341
20	1	0	-3.654287	-2.254934	-0.585415
21	1	0	-2.093935	-2.730759	-1.275777
22	1	0	-2.278027	-2.622408	0.496248
23	1	0	-3.602174	-0.307851	-1.989253
24	1	0	-2.270544	0.867904	-1.878871
25	1	0	-1.991733	-0.731017	-2.608762
26	1	0	3.564264	-0.236692	2.043476
27	1	0	2.243436	0.941502	1.875041
28	1	0	1.931165	-0.652401	2.619947
29	1	0	3.623009	-2.219826	0.753696
30	1	0	1.999217	-2.648631	1.353440

31	1	0	2.318411	-2.604579	-0.405499
32	1	0	1.089047	2.010615	-3.134621
33	1	0	1.304983	0.241496	-2.965313
34	1	0	-0.322267	0.947971	-2.916539
35	1	0	0.338661	0.962864	2.930264
36	1	0	-1.100468	1.994311	3.123316
37	1	0	-1.269820	0.219223	2.963130
38	1	0	0.840059	2.527006	1.305969
39	1	0	-0.687803	3.361841	1.052148
40	1	0	0.718259	3.380309	-1.033244
41	1	0	-0.805611	2.545177	-1.303041
42	1	0	-2.788078	1.882732	1.594618
43	1	0	-2.357296	1.944887	-0.107960
44	1	0	-2.762388	-0.635640	1.506382
45	1	0	-3.932922	0.050385	0.367584
46	1	0	2.401709	1.978477	0.077186
47	1	0	2.825638	1.862339	-1.624118
48	1	0	3.946061	0.050284	-0.333122
49	1	0	2.779514	-0.649262	-1.467936
50	1	0	-0.202671	-2.036866	-1.950505

$[Ru^{IV}(L2)(O)(OH_2)]^{2+}$ *S* = 1

Center Number	Atomic Number	Atomic Type	Coordinates (Angstroms) X	Y	Z
1	44	0	-0.062029	-0.453865	0.113315
2	8	0	0.112991	-2.303248	-1.154062
3	7	0	2.152178	-0.608529	0.568387
4	7	0	0.838123	1.051757	-1.429060
5	7	0	-2.228569	-0.602549	-0.304010
6	6	0	2.498274	-2.018775	0.882942
7	6	0	2.838201	-0.198709	-0.685555
8	6	0	2.313153	1.125327	-1.171828
9	6	0	0.206656	2.359588	-1.105190
10	6	0	-2.139072	1.596725	0.757586
11	6	0	-2.840546	0.270987	0.735814

12	6	0	-2.742086	-1.983791	-0.115994
13	6	0	0.627454	0.726426	-2.857027
14	8	0	-0.389418	-1.453884	1.535133
15	7	0	-0.662878	1.414508	1.027096
16	6	0	2.633240	0.194036	1.714520
17	6	0	-2.600006	-0.168198	-1.668394
18	6	0	0.085566	2.543573	0.391665
19	6	0	-0.465961	1.440999	2.501795
20	1	0	-3.838172	-1.976758	-0.167705
21	1	0	-2.366465	-2.624202	-0.916694
22	1	0	-2.424899	-2.362106	0.858014
23	1	0	-3.688385	-0.222231	-1.802641
24	1	0	-2.278993	0.860043	-1.854335
25	1	0	-2.124929	-0.833850	-2.394094
26	1	0	3.718590	0.074476	1.833377
27	1	0	2.418088	1.254830	1.571830
28	1	0	2.142070	-0.161065	2.623665
29	1	0	3.567272	-2.099169	1.117925
30	1	0	1.910558	-2.350881	1.741946
31	1	0	2.291625	-2.662852	0.026218
32	1	0	1.025122	1.519078	-3.505338
33	1	0	1.144579	-0.206613	-3.102405
34	1	0	-0.437180	0.603227	-3.066650
35	1	0	0.586998	1.301766	2.740832
36	1	0	-0.798449	2.407530	2.899450
37	1	0	-1.040280	0.632815	2.959351
38	1	0	1.068776	2.592777	0.871204
39	1	0	-0.422386	3.489761	0.625667
40	1	0	0.783221	3.189314	-1.540828
41	1	0	-0.775721	2.380961	-1.592390
42	1	0	-2.556779	2.254701	1.529362
43	1	0	-2.255108	2.118376	-0.196820
44	1	0	-2.734839	-0.251453	1.694025
45	1	0	-3.914743	0.398118	0.539813
46	1	0	2.507993	1.907391	-0.431836
47	1	0	2.832636	1.434361	-2.088456
48	1	0	3.926009	-0.156371	-0.527500
49	1	0	2.659418	-0.983702	-1.433501

50	1	0	0.849733	-2.513381	-1.752141
51	1	0	-0.015903	-3.103207	-0.612655

$[Ru^{III}(L2)(OH)(OH_2)]^{2+}$ $S = 1/2$

Center Number	Atomic Number	Atomic Type	Coordinates (Angstroms) X	Y	Z
1	44	0	-0.033405	-0.427380	0.031759
2	8	0	0.186900	-2.255715	-1.215032
3	7	0	2.173242	-0.607392	0.480979
4	7	0	0.807331	1.195446	-1.298506
5	7	0	-2.208065	-0.607009	-0.380165
6	6	0	2.587008	-2.023242	0.655753
7	6	0	2.864429	-0.035411	-0.701729
8	6	0	2.283029	1.307256	-1.043284
9	6	0	0.143998	2.468377	-0.905976
10	6	0	-2.191602	1.503307	0.846400
11	6	0	-2.862531	0.169750	0.704819
12	6	0	-2.697699	-2.009764	-0.323155
13	6	0	0.607829	0.939727	-2.743846
14	8	0	-0.249762	-1.743879	1.452753
15	7	0	-0.717888	1.328578	1.114072
16	6	0	2.584591	0.088726	1.719936
17	6	0	-2.586920	-0.076024	-1.709450
18	6	0	-0.000200	2.534835	0.595650
19	6	0	-0.541019	1.251439	2.587747
20	1	0	-3.792673	-2.020065	-0.395368
21	1	0	-2.288027	-2.571337	-1.164548
22	1	0	-2.391381	-2.478219	0.613672
23	1	0	-3.672803	-0.151387	-1.856230
24	1	0	-2.295154	0.972364	-1.809548
25	1	0	-2.091035	-0.667644	-2.483896
26	1	0	3.671919	0.015162	1.857837
27	1	0	2.311947	1.145662	1.686591
28	1	0	2.090904	-0.390864	2.568761
29	1	0	3.637619	-2.068269	0.969275

30	1	0	1.957494	-2.489417	1.416662
31	1	0	2.500822	-2.566340	-0.287140
32	1	0	0.987559	1.777878	-3.343351
33	1	0	1.147581	0.033759	-3.036034
34	1	0	-0.452672	0.805538	-2.963123
35	1	0	0.496130	1.030540	2.835393
36	1	0	-0.828232	2.204674	3.049145
37	1	0	-1.177715	0.462858	2.996541
38	1	0	0.975533	2.574297	1.092044
39	1	0	-0.540620	3.442853	0.898633
40	1	0	0.712959	3.333320	-1.277308
41	1	0	-0.831000	2.501270	-1.406386
42	1	0	-2.635227	2.088180	1.662032
43	1	0	-2.313098	2.095259	-0.064950
44	1	0	-2.780366	-0.419014	1.628057
45	1	0	-3.936257	0.288411	0.498214
46	1	0	2.445759	2.014421	-0.224667
47	1	0	2.774591	1.735938	-1.926391
48	1	0	3.946593	0.040204	-0.518092
49	1	0	2.732223	-0.739344	-1.534384
50	1	0	0.928516	-2.473735	-1.803135
51	1	0	0.112632	-3.003611	-0.591791
52	1	0	-0.959651	-1.603091	2.102458

$[Ru^{II}(L2)(OH_2)(OH_2)]^{2+}$ $S = 0$

Center Number	Atomic Number	Atomic Type	Coordinates (Angstroms) X	Y	Z
1	44	0	0.000047	-0.404533	0.000085
2	8	0	-0.493753	-1.969360	1.578420
3	7	0	-2.208698	-0.565748	-0.444359
4	7	0	-0.706836	1.227142	1.243316
5	7	0	2.208601	-0.565997	0.444407
6	6	0	-2.662637	-1.972131	-0.535465
7	6	0	-2.824735	0.041508	0.766413
8	6	0	-2.193829	1.375489	1.037881

9	6	0	-0.038809	2.468907	0.754512
10	6	0	2.194161	1.374937	-1.038093
11	6	0	2.824752	0.040823	-0.766472
12	6	0	2.662113	-1.972482	0.535848
13	6	0	-0.475637	1.075141	2.696959
14	8	0	0.493111	-1.969262	-1.578468
15	7	0	0.707108	1.226935	-1.243390
16	6	0	-2.707323	0.108833	-1.660444
17	6	0	2.707330	0.108790	1.660327
18	6	0	0.039392	2.468895	-0.754667
19	6	0	0.475720	1.074895	-2.696995
20	1	0	3.751486	-2.018733	0.672070
21	1	0	2.194488	-2.459000	1.396800
22	1	0	2.413899	-2.509895	-0.380078
23	1	0	3.804331	0.057713	1.715002
24	1	0	2.407130	1.158347	1.680083
25	1	0	2.301243	-0.391048	2.544894
26	1	0	-3.804346	0.058154	-1.714963
27	1	0	-2.406698	1.158263	-1.680655
28	1	0	-2.301581	-0.391472	-2.544916
29	1	0	-3.751978	-2.018047	-0.672049
30	1	0	-2.194879	-2.459095	-1.396090
31	1	0	-2.414943	-2.509307	0.380733
32	1	0	-0.791380	1.976331	3.240099
33	1	0	-1.063500	0.230263	3.069118
34	1	0	0.583525	0.901746	2.893305
35	1	0	-0.583557	0.902022	-2.893231
36	1	0	0.791846	1.975875	-3.240263
37	1	0	1.063190	0.229728	-3.069130
38	1	0	-0.957468	2.507658	-1.207547
39	1	0	0.579429	3.357690	-1.113320
40	1	0	-0.578622	3.357865	1.113097
41	1	0	0.958061	2.507482	1.207392
42	1	0	2.636389	1.848881	-1.923826
43	1	0	2.365736	2.060389	-0.203202
44	1	0	2.654681	-0.650603	-1.600154
45	1	0	3.913329	0.142679	-0.637769
46	1	0	-2.365159	2.060853	0.202874

47	1	0	-2.636009	1.849667	1.923513
48	1	0	-3.913279	0.143649	0.637667
49	1	0	-2.654910	-0.649849	1.600200
50	1	0	-0.019425	-1.846087	2.416416
51	1	0	-0.434538	-2.923390	1.404935
52	1	0	0.017610	-1.844430	-2.415550
53	1	0	0.434760	-2.923796	-1.407447

$[Fe^{V}(L1)(O)(OH)]^{2+}$ $S = 3/2$

Center Number	Atomic Number	Atomic Type	Coordinates (Angstroms) X	Y	Z
1	7	0	0.728307	-1.080886	0.449049
2	6	0	1.848697	-0.632096	1.033770
3	6	0	2.203606	-1.087851	2.297673
4	6	0	1.357800	-2.002226	2.929930
5	6	0	0.186485	-2.439978	2.307344
6	6	0	-0.108612	-1.944130	1.043155
7	6	0	2.634767	0.294484	0.151740
8	7	0	1.746952	1.065075	-0.791865
9	6	0	2.482079	1.372982	-2.049633
10	6	0	-1.270174	-2.323098	0.170464
11	7	0	-1.651907	-1.213278	-0.775578
12	26	0	0.071019	-0.112104	-1.196901
13	8	0	0.868906	-1.309935	-2.252548
14	6	0	-2.641870	-0.267739	-0.153105
15	6	0	-1.954913	0.806122	0.628719
16	6	0	-2.492085	1.556493	1.667658
17	6	0	-1.736354	2.608328	2.187035
18	6	0	-0.479726	2.905430	1.658021
19	6	0	0.003416	2.118841	0.619340
20	7	0	-0.734465	1.099182	0.155127
21	6	0	1.252555	2.342798	-0.171755
22	6	0	-2.224318	-1.781863	-2.027079
23	8	0	-0.541110	0.792357	-2.417497
24	1	0	-0.475870	-3.148713	2.798790

25	1	0	1.610606	-2.372276	3.921119
26	1	0	3.114077	-0.742282	2.781576
27	1	0	3.258134	0.994678	0.721929
28	1	0	3.317889	-0.298750	-0.470533
29	1	0	1.016644	3.024825	-0.999217
30	1	0	2.057601	2.808503	0.410510
31	1	0	0.105986	3.738652	2.038843
32	1	0	-2.134373	3.207923	3.002521
33	1	0	-3.482819	1.332982	2.056030
34	1	0	-3.374654	-0.832876	0.436522
35	1	0	-3.188576	0.205946	-0.979085
36	1	0	-0.985088	-3.183151	-0.449892
37	1	0	-2.150910	-2.631132	0.747867
38	1	0	1.810034	1.892892	-2.735201
39	1	0	3.351449	2.004161	-1.825804
40	1	0	2.806461	0.440157	-2.514003
41	1	0	-3.136966	-2.345218	-1.794712
42	1	0	-2.454442	-0.965728	-2.714772
43	1	0	-1.487764	-2.438403	-2.493416
44	1	0	1.334722	-2.001239	-1.744630

$[Fe^{IV}(L1)(O)(OH_2)]^{2+}$ $S = 2$

Center Number	Atomic Number	Atomic Type	Coordinates (Angstroms) X	Y	Z
1	6	0	-0.026054	0.267209	0.096637
2	6	0	0.002244	0.086467	1.476516
3	7	0	1.173970	0.005288	2.112071
4	6	0	2.354708	0.120781	1.485962
5	6	0	2.394705	0.325965	0.113296
6	6	0	1.185645	0.379723	-0.583113
7	6	0	-1.217868	0.073117	2.361490
8	7	0	-0.959884	-0.487307	3.724043
9	6	0	-1.560735	-1.833488	3.861139
10	26	0	1.219949	-0.565783	4.085158
11	8	0	1.628766	-2.098089	3.728042

12	6	0	3.543147	-0.023880	2.397762
13	7	0	3.173931	0.373627	3.785615
14	6	0	4.149469	-0.159287	4.761697
15	6	0	3.027674	1.858711	3.919331
16	6	0	1.719735	2.278418	4.536519
17	6	0	1.430224	3.576521	4.948261
18	6	0	0.129586	3.875830	5.349191
19	6	0	-0.866044	2.896348	5.306826
20	6	0	-0.514997	1.613984	4.909621
21	7	0	0.763098	1.334510	4.574021
22	6	0	-1.445170	0.435861	4.790445
23	8	0	1.056639	-1.112284	6.127959
24	1	0	-1.893727	3.131834	5.573659
25	1	0	-0.118120	4.885160	5.670403
26	1	0	2.200666	4.344214	4.936830
27	1	0	3.080250	2.305781	2.916649
28	1	0	3.881739	2.266795	4.475362
29	1	0	3.850683	-1.077156	2.436523
30	1	0	4.404988	0.558485	2.041918
31	1	0	3.345523	0.429098	-0.404637
32	1	0	1.189128	0.518983	-1.661813
33	1	0	-0.973327	0.325722	-0.434859
34	1	0	-1.567698	1.109316	2.470154
35	1	0	-2.039953	-0.477939	1.887761
36	1	0	-1.467504	-0.134572	5.728245
37	1	0	-2.478645	0.756195	4.594419
38	1	0	4.149733	-1.251717	4.713284
39	1	0	5.161332	0.210585	4.549847
40	1	0	3.866359	0.159990	5.769194
41	1	0	-2.654204	-1.785793	3.775007
42	1	0	-1.159694	-2.483693	3.080021
43	1	0	-1.296056	-2.254135	4.834577
44	1	0	1.144255	-0.609059	6.954531
45	1	0	1.337794	-2.025507	6.313380

$[Fe^{III}(L1)(OH)(OH_2)]^{2+}$ $S = 5/2$

Center Number	Atomic Number	Atomic Type	Coordinates (Angstroms) X	Y	Z
1	7	0	0.090483	0.036104	1.372460
2	6	0	0.776012	-1.072671	1.703457
3	6	0	1.965481	-0.994537	2.418690
4	6	0	2.452872	0.264705	2.766597
5	6	0	1.762075	1.408916	2.369297
6	6	0	0.578830	1.256830	1.655638
7	6	0	0.139837	-2.355796	1.249514
8	7	0	-0.502772	-2.218641	-0.088188
9	6	0	-1.573107	-3.231571	-0.234997
10	6	0	-0.262534	2.395234	1.152286
11	7	0	-0.868903	2.097775	-0.176297
12	26	0	-1.323649	-0.116766	-0.237195
13	8	0	-2.819507	-0.213999	1.320804
14	6	0	0.080962	2.370222	-1.292769
15	6	0	1.034181	1.232507	-1.519141
16	6	0	2.347031	1.371723	-1.955306
17	6	0	3.107085	0.220080	-2.156739
18	6	0	2.551353	-1.033988	-1.905839
19	6	0	1.232282	-1.100398	-1.471208
20	7	0	0.505017	0.016445	-1.312129
21	6	0	0.483668	-2.372339	-1.195546
22	6	0	-2.093783	2.909688	-0.359778
23	8	0	-2.549766	-0.247989	-1.521744
24	1	0	2.143442	2.401224	2.599865
25	1	0	3.383033	0.354875	3.323414
26	1	0	2.507342	-1.898424	2.688238
27	1	0	0.862550	-3.184233	1.247087
28	1	0	-0.650994	-2.636083	1.958582
29	1	0	-0.085610	-2.642764	-2.094990
30	1	0	1.165750	-3.209558	-0.989416
31	1	0	3.134060	-1.942238	-2.042302
32	1	0	4.137547	0.300640	-2.495699
33	1	0	2.768809	2.358846	-2.130742

34	1	0	0.611480	3.318157	-1.123557
35	1	0	-0.522944	2.503821	-2.200322
36	1	0	-1.091402	2.567232	1.852407
37	1	0	0.310629	3.332569	1.113440
38	1	0	-2.058027	-3.107285	-1.205666
39	1	0	-1.168635	-4.250271	-0.162300
40	1	0	-2.316751	-3.092135	0.554204
41	1	0	-1.866476	3.983960	-0.329735
42	1	0	-2.547941	2.666673	-1.322837
43	1	0	-2.805895	2.679373	0.437188
44	1	0	-2.741239	-0.186022	2.288156
45	1	0	-3.267309	-0.319805	-2.165465
46	1	0	-3.764605	-0.298614	1.111941

$[Fe^{II}(L1)(OH_2)(OH_2)]^{2+}$ $S = 2$

Center Number	Atomic Number	Atomic Type	Coordinates (Angstroms) X	Y	Z
1	7	0	0.478951	0.117202	-1.364921
2	6	0	1.029656	1.343215	-1.402314
3	6	0	2.376565	1.522150	-1.697892
4	6	0	3.163717	0.395516	-1.931826
5	6	0	2.597034	-0.874303	-1.830229
6	6	0	1.243143	-0.976636	-1.530658
7	6	0	0.067890	2.459925	-1.107848
8	7	0	-0.888201	2.107741	-0.016347
9	6	0	-2.084388	2.970734	-0.123164
10	6	0	0.502806	-2.274545	-1.369848
11	7	0	-0.485801	-2.225034	-0.252024
12	26	0	-1.306994	-0.111358	-0.222425
13	8	0	-2.610328	-0.110391	-1.963757
14	6	0	0.175041	-2.439513	1.069643
15	6	0	0.818826	-1.186144	1.592458
16	7	0	0.104782	-0.067815	1.375135
17	6	0	0.600900	1.133324	1.720179
18	6	0	1.816374	1.249752	2.385236

19	6	0	2.535059	0.089365	2.669652
20	6	0	2.041500	-1.146309	2.253550
21	6	0	-0.270453	2.293986	1.330141
22	6	0	-1.504428	-3.276687	-0.460489
23	8	0	-3.023713	-0.369710	1.087807
24	1	0	2.200201	2.228408	2.665571
25	1	0	3.488445	0.150357	3.189578
26	1	0	2.603249	-2.061214	2.429792
27	1	0	0.895893	-3.268132	1.009669
28	1	0	-0.609995	-2.759872	1.768474
29	1	0	-0.066119	-2.480729	-2.287039
30	1	0	1.194142	-3.119388	-1.235135
31	1	0	3.200137	-1.768938	-1.970135
32	1	0	4.220167	0.505644	-2.165858
33	1	0	2.805445	2.521395	-1.733191
34	1	0	0.596333	3.394795	-0.869941
35	1	0	-0.534575	2.662408	-2.004127
36	1	0	-1.097215	2.381122	2.048677
37	1	0	0.281058	3.244719	1.372435
38	1	0	-2.008903	-3.113093	-1.416904
39	1	0	-1.055644	-4.279721	-0.469772
40	1	0	-2.243607	-3.234288	0.344215
41	1	0	-1.828479	4.033821	-0.013942
42	1	0	-2.554176	2.825080	-1.099801
43	1	0	-2.799559	2.703501	0.659844
44	1	0	-2.904345	-0.395970	2.050940
45	1	0	-3.975825	-0.487353	0.940055
46	1	0	-2.232799	-0.041836	-2.855576
47	1	0	-3.571616	-0.155414	-2.090621

----

^{4}**RC1** *S* = 3/2

Center Number	Atomic Number	Atomic Type	Coordinates (Angstroms) X	Y	Z
1	6	0	0.722240	1.181480	2.901030
2	6	0	0.940480	0.858580	1.562470

3	7	0	0.468040	-0.281060	1.057560
4	6	0	-0.241010	-1.142760	1.794130
5	6	0	-0.500830	-0.886900	3.134810
6	6	0	-0.004060	0.293770	3.688750
7	6	0	1.753300	1.688990	0.611660
8	7	0	1.412770	1.433240	-0.812650
9	6	0	2.611400	1.545720	-1.668020
10	26	0	0.561810	-0.632380	-1.062620
11	8	0	1.818860	-1.623320	-0.776450
12	6	0	-0.660840	-2.375830	1.045960
13	7	0	-1.004080	-2.071700	-0.365060
14	6	0	-0.902320	-3.288160	-1.194070
15	6	0	-2.346670	-1.454150	-0.491690
16	6	0	-2.316370	0.045530	-0.366030
17	7	0	-1.171160	0.607520	-0.748860
18	6	0	-1.000800	1.931880	-0.776630
19	6	0	-2.030390	2.785120	-0.393950
20	6	0	-3.236580	2.219640	0.020230
21	6	0	-3.391300	0.834960	0.036580
22	6	0	0.348040	2.347560	-1.290340
23	8	0	0.232180	-0.520320	-2.638000
24	8	0	3.827980	-0.504470	0.809010
25	1	0	-1.078970	-1.588910	3.731760
26	1	0	-0.192920	0.524620	4.734910
27	1	0	1.112040	2.109130	3.314450
28	1	0	1.659930	2.761860	0.837650
29	1	0	2.803360	1.403650	0.755290
30	1	0	0.336840	2.279130	-2.386210
31	1	0	0.585550	3.388440	-1.022580
32	1	0	-1.893340	3.864070	-0.414590
33	1	0	-4.057210	2.860870	0.334450
34	1	0	-4.326480	0.378810	0.354480
35	1	0	-3.048450	-1.905230	0.226210
36	1	0	-2.720140	-1.698030	-1.495080
37	1	0	0.187660	-3.071670	1.019470
38	1	0	-1.496810	-2.890190	1.544340
39	1	0	2.343690	1.263900	-2.689770
40	1	0	3.004360	2.572760	-1.659180

41	1	0	3.375620	0.861770	-1.289930
42	1	0	-1.620050	-4.053840	-0.866000
43	1	0	-1.095470	-3.024850	-2.236760
44	1	0	0.114940	-3.679910	-1.119160
45	1	0	4.684360	-0.940000	0.909470
46	1	0	3.277450	-1.118080	0.282230

----

[4]**TS1** *S* = 3/2

Center Number	Atomic Number	Atomic Type	Coordinates (Angstroms) X	Y	Z
1	6	0	-2.181830	-1.448026	-2.184553
2	6	0	-0.942898	-1.254108	-1.579850
3	7	0	-0.501484	-0.020439	-1.298743
4	6	0	-1.246953	1.061145	-1.564893
5	6	0	-2.493900	0.940680	-2.169770
6	6	0	-2.960664	-0.333559	-2.484906
7	6	0	0.010172	-2.367965	-1.242163
8	7	0	0.833809	-2.067712	-0.055407
9	6	0	2.142050	-2.745722	-0.125289
10	26	0	1.197953	0.195059	-0.038903
11	8	0	2.276604	0.367626	-1.352773
12	6	0	-0.607235	2.371524	-1.201034
13	7	0	0.209284	2.273927	0.026504
14	6	0	1.257881	3.308549	0.034099
15	6	0	-0.605096	2.339221	1.256829
16	6	0	-1.185061	1.003783	1.635544
17	7	0	-0.420626	-0.053676	1.332991
18	6	0	-0.815864	-1.302222	1.620755
19	6	0	-2.020238	-1.536146	2.277152
20	6	0	-2.817424	-0.445984	2.618455
21	6	0	-2.402505	0.842383	2.290566
22	6	0	0.148265	-2.381430	1.213031
23	8	0	2.361747	0.253819	1.146701
24	8	0	4.195874	0.037069	0.054848
25	1	0	-3.089486	1.825962	-2.381793

26	1	0	-3.935540	-0.458234	-2.951304
27	1	0	-2.530923	-2.453789	-2.408348
28	1	0	-0.524702	-3.324853	-1.129707
29	1	0	0.700113	-2.488067	-2.088248
30	1	0	0.926482	-2.453768	1.984742
31	1	0	-0.351688	-3.362394	1.167420
32	1	0	-2.330799	-2.553121	2.506848
33	1	0	-3.767528	-0.601353	3.125017
34	1	0	-3.015581	1.708340	2.531017
35	1	0	-1.394361	3.103709	1.174713
36	1	0	0.062447	2.661241	2.067607
37	1	0	0.074276	2.654032	-2.014742
38	1	0	-1.359255	3.172636	-1.117847
39	1	0	2.761846	-2.408192	0.709240
40	1	0	2.032559	-3.840042	-0.093640
41	1	0	2.642705	-2.455411	-1.052318
42	1	0	0.830989	4.322704	0.039950
43	1	0	1.882718	3.174922	0.921114
44	1	0	1.884783	3.183389	-0.852671
45	1	0	4.475737	0.849240	0.508222
46	1	0	3.326568	0.290154	-0.791333

----

[4]INT1 *S* = 3/2

Center Number	Atomic Number	Atomic Type	Coordinates (Angstroms) X	Y	Z
1	6	0	-2.424624	-1.367348	-1.964534
2	6	0	-1.113992	-1.210679	-1.524706
3	7	0	-0.604114	0.009210	-1.288098
4	6	0	-1.359308	1.110637	-1.438429
5	6	0	-2.675450	1.023494	-1.879321
6	6	0	-3.211085	-0.233432	-2.149667
7	6	0	-0.155415	-2.351646	-1.325197
8	7	0	0.800362	-2.095221	-0.230921
9	6	0	2.076754	-2.791248	-0.463963
10	26	0	1.137084	0.158061	-0.202167

11	8	0	2.109660	0.468037	-1.679160
12	6	0	-0.659777	2.403551	-1.129656
13	7	0	0.270019	2.274017	0.009791
14	6	0	1.334153	3.285678	-0.071838
15	6	0	-0.409625	2.320032	1.317536
16	6	0	-0.958049	0.980823	1.724354
17	7	0	-0.233154	-0.079967	1.327263
18	6	0	-0.632367	-1.329797	1.627394
19	6	0	-1.771960	-1.557924	2.390753
20	6	0	-2.515504	-0.466279	2.832195
21	6	0	-2.109070	0.820417	2.488685
22	6	0	0.255498	-2.421670	1.101250
23	8	0	2.500076	0.191998	0.978982
24	8	0	3.829893	0.017349	0.380862
25	1	0	-3.271414	1.925919	-1.997885
26	1	0	-4.240320	-0.329487	-2.488155
27	1	0	-2.821251	-2.363041	-2.151498
28	1	0	-0.695433	-3.300816	-1.176932
29	1	0	0.435517	-2.461929	-2.244419
30	1	0	1.114720	-2.523949	1.777599
31	1	0	-0.269325	-3.390794	1.097686
32	1	0	-2.076230	-2.576274	2.623147
33	1	0	-3.415270	-0.618105	3.424137
34	1	0	-2.681426	1.691888	2.799591
35	1	0	-1.197720	3.090536	1.338695
36	1	0	0.343070	2.620131	2.059431
37	1	0	-0.052229	2.679347	-2.001743
38	1	0	-1.385329	3.217414	-0.967953
39	1	0	2.783007	-2.511504	0.321874
40	1	0	1.950764	-3.884554	-0.473679
41	1	0	2.488288	-2.464326	-1.422653
42	1	0	0.935844	4.309496	-0.000999
43	1	0	2.045709	3.120417	0.742005
44	1	0	1.861617	3.165743	-1.021799
45	1	0	4.370780	0.483463	1.042092
46	1	0	3.042554	0.547842	-1.403034

----

[6]**RC1** *S* = 5/2

Center Number	Atomic Number	Atomic Type	Coordinates (Angstroms) X	Y	Z
1	6	0	0.718693	1.177106	2.910991
2	6	0	0.956563	0.845639	1.578504
3	7	0	0.492434	-0.305599	1.069231
4	6	0	-0.253077	-1.144022	1.810072
5	6	0	-0.525111	-0.870058	3.144444
6	6	0	-0.020646	0.304080	3.700839
7	6	0	1.770647	1.704730	0.654202
8	7	0	1.434089	1.498608	-0.771099
9	6	0	2.629072	1.645583	-1.624686
10	26	0	0.545044	-0.606728	-0.996106
11	8	0	1.873286	-1.670294	-1.019482
12	6	0	-0.722613	-2.378293	1.093692
13	7	0	-1.052874	-2.107897	-0.319540
14	6	0	-0.947713	-3.338884	-1.123773
15	6	0	-2.371736	-1.469728	-0.493053
16	6	0	-2.313907	0.032070	-0.417381
17	7	0	-1.161366	0.593847	-0.810636
18	6	0	-0.994899	1.926981	-0.792832
19	6	0	-2.030988	2.769932	-0.406973
20	6	0	-3.244783	2.204672	-0.022010
21	6	0	-3.389683	0.820517	-0.017660
22	6	0	0.359820	2.396141	-1.238332
23	8	0	0.479983	-0.736117	-2.688070
24	8	0	3.843743	-0.557181	0.609115
25	1	0	-1.127242	-1.558983	3.732617
26	1	0	-0.220827	0.545417	4.742541
27	1	0	1.104317	2.110535	3.315040
28	1	0	1.684522	2.766753	0.933970
29	1	0	2.819874	1.408965	0.785898
30	1	0	0.387237	2.382202	-2.336742
31	1	0	0.541523	3.436673	-0.924241
32	1	0	-1.884287	3.847666	-0.399165
33	1	0	-4.069617	2.841306	0.290617

34	1	0	-4.320380	0.354273	0.298394
35	1	0	-3.109578	-1.867218	0.222262
36	1	0	-2.733612	-1.741568	-1.493934
37	1	0	0.096427	-3.110233	1.092116
38	1	0	-1.569304	-2.844152	1.623327
39	1	0	2.369424	1.361861	-2.648102
40	1	0	3.003941	2.680148	-1.613545
41	1	0	3.409111	0.975604	-1.255289
42	1	0	-1.673595	-4.099285	-0.798762
43	1	0	-1.119713	-3.093707	-2.174597
44	1	0	0.066059	-3.735729	-1.031641
45	1	0	4.707045	-0.952868	0.787709
46	1	0	3.411370	-1.151542	-0.034673

----

^{6}**TS1** *S* = 5/2

Center Number	Atomic Number	Atomic Type	Coordinates (Angstroms) X	Y	Z
1	6	0	-2.166489	-1.451336	-2.209882
2	6	0	-0.930538	-1.248825	-1.602364
3	7	0	-0.499274	-0.011381	-1.319688
4	6	0	-1.255187	1.064657	-1.579698
5	6	0	-2.500095	0.934306	-2.187225
6	6	0	-2.954121	-0.342670	-2.509239
7	6	0	0.027738	-2.356851	-1.260199
8	7	0	0.836427	-2.056555	-0.063036
9	6	0	2.147225	-2.731317	-0.120538
10	26	0	1.177437	0.204650	-0.036736
11	8	0	2.295395	0.394396	-1.319118
12	6	0	-0.632233	2.380846	-1.206005
13	7	0	0.190277	2.284929	0.016816
14	6	0	1.239628	3.318965	0.020837
15	6	0	-0.614530	2.347783	1.252837
16	6	0	-1.186917	1.011423	1.637578
17	7	0	-0.429584	-0.047222	1.317311
18	6	0	-0.817029	-1.296320	1.615417

19	6	0	-2.006574	-1.529397	2.298688
20	6	0	-2.795693	-0.439338	2.657814
21	6	0	-2.388597	0.848975	2.320332
22	6	0	0.140086	-2.377399	1.197634
23	8	0	2.306820	0.283628	1.264623
24	8	0	4.190818	-0.088308	0.079551
25	1	0	-3.104029	1.814802	-2.395479
26	1	0	-3.926745	-0.474328	-2.978396
27	1	0	-2.506875	-2.459512	-2.436176
28	1	0	-0.501639	-3.318045	-1.159054
29	1	0	0.727947	-2.467910	-2.099094
30	1	0	0.912721	-2.463429	1.973533
31	1	0	-0.367330	-3.353930	1.139681
32	1	0	-2.311201	-2.546234	2.536506
33	1	0	-3.733677	-0.594611	3.186533
34	1	0	-2.995447	1.714999	2.575655
35	1	0	-1.406508	3.110426	1.178954
36	1	0	0.059567	2.669423	2.058308
37	1	0	0.041202	2.680556	-2.020338
38	1	0	-1.396143	3.169836	-1.115151
39	1	0	2.755305	-2.396766	0.723727
40	1	0	2.039986	-3.826146	-0.097144
41	1	0	2.659674	-2.434496	-1.039021
42	1	0	0.813638	4.333504	0.036043
43	1	0	1.872163	3.178213	0.901267
44	1	0	1.859054	3.197791	-0.871527
45	1	0	4.567231	0.719852	0.470115
46	1	0	3.299333	0.233265	-0.771525

----

[6]**INT1** *S* = 5/2

Center Number	Atomic Number	Atomic Type	Coordinates (Angstroms) X	Y	Z
1	6	0	-2.297981	-1.484240	-2.051165
2	6	0	-1.005737	-1.273117	-1.578813
3	7	0	-0.546166	-0.036349	-1.358191

4	6	0	-1.324382	1.036570	-1.539751
5	6	0	-2.627586	0.905733	-2.010724
6	6	0	-3.110790	-0.374535	-2.274990
7	6	0	-0.004662	-2.363454	-1.304890
8	7	0	0.853612	-2.054837	-0.138043
9	6	0	2.138885	-2.770340	-0.238220
10	26	0	1.291883	0.198001	-0.176375
11	8	0	2.432189	0.387387	-1.596962
12	6	0	-0.654722	2.346867	-1.223121
13	7	0	0.254401	2.242505	-0.058537
14	6	0	1.294221	3.285933	-0.120470
15	6	0	-0.476384	2.321488	1.228957
16	6	0	-1.102936	1.005950	1.604307
17	7	0	-0.338588	-0.057473	1.334684
18	6	0	-0.777764	-1.299366	1.564893
19	6	0	-2.020529	-1.527177	2.149254
20	6	0	-2.810945	-0.425887	2.474203
21	6	0	-2.357529	0.861255	2.190358
22	6	0	0.186168	-2.376066	1.146289
23	8	0	2.382030	0.319477	1.393885
24	8	0	3.761216	0.516464	1.032792
25	1	0	-3.253850	1.783226	-2.157955
26	1	0	-4.127520	-0.508583	-2.638364
27	1	0	-2.663957	-2.493087	-2.230490
28	1	0	-0.499368	-3.340808	-1.186011
29	1	0	0.659881	-2.443801	-2.175749
30	1	0	0.977274	-2.445451	1.904442
31	1	0	-0.306108	-3.360641	1.098067
32	1	0	-2.368220	-2.541469	2.334949
33	1	0	-3.789400	-0.571715	2.927153
34	1	0	-2.971338	1.732947	2.408370
35	1	0	-1.223237	3.131322	1.208186
36	1	0	0.263760	2.582660	1.996724
37	1	0	-0.035732	2.631622	-2.084645
38	1	0	-1.394631	3.150351	-1.078012
39	1	0	2.778289	-2.468142	0.596069
40	1	0	2.001704	-3.862059	-0.215402
41	1	0	2.631961	-2.485291	-1.171238

42	1	0	0.862751	4.296705	-0.062225
43	1	0	1.991014	3.141362	0.710078
44	1	0	1.847542	3.180229	-1.057266
45	1	0	4.173608	0.559092	1.913006
46	1	0	3.374836	0.518001	-1.411132

MECP1 *S* = 3/2 or *S* = 5/2

Center Number	Atomic Number	Atomic Type	Coordinates (Angstroms) X	Y	Z
1	6	0	-2.439645	-1.370900	-1.962980
2	6	0	-1.128381	-1.209762	-1.524625
3	7	0	-0.621979	0.009973	-1.296800
4	6	0	-1.377052	1.109842	-1.439158
5	6	0	-2.694274	1.022839	-1.876033
6	6	0	-3.224750	-0.235977	-2.149884
7	6	0	-0.162128	-2.344237	-1.320903
8	7	0	0.800256	-2.080007	-0.230994
9	6	0	2.071746	-2.785635	-0.470492
10	26	0	1.181323	0.167540	-0.208529
11	8	0	2.157935	0.446179	-1.711649
12	6	0	-0.670017	2.399236	-1.128450
13	7	0	0.267291	2.262155	0.006126
14	6	0	1.330030	3.277901	-0.076965
15	6	0	-0.411267	2.315192	1.317373
16	6	0	-0.964930	0.979413	1.730884
17	7	0	-0.232525	-0.078570	1.349304
18	6	0	-0.632548	-1.328938	1.635797
19	6	0	-1.779691	-1.562899	2.386155
20	6	0	-2.526444	-0.471399	2.823635
21	6	0	-2.125220	0.817524	2.481590
22	6	0	0.259870	-2.414043	1.103150
23	8	0	2.559105	0.209496	1.014354
24	8	0	3.877953	0.095251	0.412103
25	1	0	-3.295490	1.922513	-1.991940
26	1	0	-4.253140	-0.332778	-2.490989

27	1	0	-2.839289	-2.365899	-2.147273
28	1	0	-0.694941	-3.295627	-1.162978
29	1	0	0.424525	-2.457750	-2.242261
30	1	0	1.120775	-2.515496	1.776983
31	1	0	-0.259585	-3.385948	1.095705
32	1	0	-2.086517	-2.581582	2.613943
33	1	0	-3.429089	-0.625725	3.410674
34	1	0	-2.706790	1.685511	2.785576
35	1	0	-1.194308	3.090655	1.334715
36	1	0	0.344125	2.614180	2.056637
37	1	0	-0.067324	2.676984	-2.003329
38	1	0	-1.390042	3.215846	-0.957744
39	1	0	2.782511	-2.514285	0.314379
40	1	0	1.936272	-3.877711	-0.483636
41	1	0	2.482332	-2.457398	-1.428986
42	1	0	0.929620	4.299660	0.006809
43	1	0	2.051025	3.105658	0.727062
44	1	0	1.848118	3.167722	-1.033186
45	1	0	4.431243	0.443864	1.133019
46	1	0	3.097239	0.542944	-1.469274

----

^{5}**INT2** *S* = 2

Center Number	Atomic Number	Atomic Type	Coordinates (Angstroms) X	Y	Z
1	6	0	-2.293523	-1.483525	-2.070557
2	6	0	-0.998075	-1.271645	-1.608160
3	7	0	-0.536180	-0.031824	-1.399916
4	6	0	-1.318934	1.041248	-1.573354
5	6	0	-2.624589	0.906531	-2.035518
6	6	0	-3.107721	-0.374857	-2.294998
7	6	0	0.000474	-2.362377	-1.327291
8	7	0	0.847218	-2.059286	-0.149060
9	6	0	2.134650	-2.773368	-0.243022
10	26	0	1.235938	0.196748	-0.192649
11	8	0	2.467561	0.398375	-1.508326

12	6	0	-0.654154	2.352580	-1.251532
13	7	0	0.237834	2.253652	-0.071765
14	6	0	1.277406	3.298987	-0.124561
15	6	0	-0.509459	2.332200	1.203991
16	6	0	-1.110240	1.008892	1.594807
17	7	0	-0.353222	-0.055171	1.297570
18	6	0	-0.778207	-1.298332	1.558348
19	6	0	-1.993332	-1.523837	2.198019
20	6	0	-2.773807	-0.423596	2.547762
21	6	0	-2.337022	0.862276	2.235689
22	6	0	0.168973	-2.384656	1.126217
23	8	0	2.428715	0.339304	1.275015
24	8	0	3.697276	0.520458	1.199106
25	1	0	-3.253436	1.782678	-2.179004
26	1	0	-4.126372	-0.510697	-2.652153
27	1	0	-2.661031	-2.493089	-2.241980
28	1	0	-0.495930	-3.339829	-1.218442
29	1	0	0.674825	-2.440058	-2.190761
30	1	0	0.953579	-2.482796	1.888516
31	1	0	-0.342346	-3.358801	1.066864
32	1	0	-2.326808	-2.537980	2.408165
33	1	0	-3.731022	-0.569322	3.043903
34	1	0	-2.942196	1.734088	2.475581
35	1	0	-1.276531	3.122221	1.167039
36	1	0	0.212032	2.625444	1.978502
37	1	0	-0.021556	2.634967	-2.103839
38	1	0	-1.396766	3.155921	-1.121631
39	1	0	2.766695	-2.483341	0.601279
40	1	0	1.997688	-3.865080	-0.234391
41	1	0	2.637680	-2.477567	-1.167177
42	1	0	0.843167	4.309148	-0.083463
43	1	0	1.960873	3.166632	0.719105
44	1	0	1.847315	3.185707	-1.050324
45	1	0	3.396932	0.524151	-1.255648

----

[7]**INT2** *S* = 3

Center Number	Atomic Number	Atomic Type	Coordinates (Angstroms) X	Y	Z
1	6	0	-2.293523	-1.483525	-2.070557
2	6	0	-0.998075	-1.271645	-1.608160
3	7	0	-0.536180	-0.031824	-1.399916
4	6	0	-1.318934	1.041248	-1.573354
5	6	0	-2.624589	0.906531	-2.035518
6	6	0	-3.107721	-0.374857	-2.294998
7	6	0	0.000474	-2.362377	-1.327291
8	7	0	0.847218	-2.059286	-0.149060
9	6	0	2.134650	-2.773368	-0.243022
10	26	0	1.235938	0.196748	-0.192649
11	8	0	2.467561	0.398375	-1.508326
12	6	0	-0.654154	2.352580	-1.251532
13	7	0	0.237834	2.253652	-0.071765
14	6	0	1.277406	3.298987	-0.124561
15	6	0	-0.509459	2.332200	1.203991
16	6	0	-1.110240	1.008892	1.594807
17	7	0	-0.353222	-0.055171	1.297570
18	6	0	-0.778207	-1.298332	1.558348
19	6	0	-1.993332	-1.523837	2.198019
20	6	0	-2.773807	-0.423596	2.547762
21	6	0	-2.337022	0.862276	2.235689
22	6	0	0.168973	-2.384656	1.126217
23	8	0	2.428715	0.339304	1.275015
24	8	0	3.697276	0.520458	1.199106
25	1	0	-3.253436	1.782678	-2.179004
26	1	0	-4.126372	-0.510697	-2.652153
27	1	0	-2.661031	-2.493089	-2.241980
28	1	0	-0.495930	-3.339829	-1.218442
29	1	0	0.674825	-2.440058	-2.190761
30	1	0	0.953579	-2.482796	1.888516
31	1	0	-0.342346	-3.358801	1.066864
32	1	0	-2.326808	-2.537980	2.408165
33	1	0	-3.731022	-0.569322	3.043903

34	1	0	-2.942196	1.734088	2.475581
35	1	0	-1.276531	3.122221	1.167039
36	1	0	0.212032	2.625444	1.978502
37	1	0	-0.021556	2.634967	-2.103839
38	1	0	-1.396766	3.155921	-1.121631
39	1	0	2.766695	-2.483341	0.601279
40	1	0	1.997688	-3.865080	-0.234391
41	1	0	2.637680	-2.477567	-1.167177
42	1	0	0.843167	4.309148	-0.083463
43	1	0	1.960873	3.166632	0.719105
44	1	0	1.847315	3.185707	-1.050324
45	1	0	3.396932	0.524151	-1.255648

----

[5]INT3 *S* = 2

Center Number	Atomic Number	Atomic Type	Coordinates (Angstroms) X	Y	Z
1	6	0	1.754268	1.003415	-2.750798
2	6	0	0.753657	1.076962	-1.786007
3	7	0	0.174156	-0.025154	-1.297342
4	6	0	0.565206	-1.241480	-1.699929
5	6	0	1.559491	-1.393967	-2.662934
6	6	0	2.153832	-0.253667	-3.197144
7	6	0	0.204770	2.367207	-1.249964
8	7	0	-0.211668	2.273974	0.168505
9	6	0	-1.099792	3.414436	0.469900
10	26	0	-1.179381	0.129245	0.464904
11	8	0	-1.695076	-0.065838	2.272982
12	6	0	-0.164750	-2.396526	-1.072568
13	7	0	-0.539037	-2.138456	0.332456
14	6	0	-1.702004	-2.959065	0.712723
15	6	0	0.583206	-2.383648	1.257901
16	6	0	1.551542	-1.234813	1.281928
17	7	0	0.980134	-0.025882	1.215902
18	6	0	1.730974	1.080486	1.183914
19	6	0	3.119840	1.018934	1.266275

20	6	0	3.723826	-0.231784	1.372215
21	6	0	2.933629	-1.378917	1.372431
22	6	0	0.954362	2.360063	1.082929
23	8	0	-2.385598	-0.445961	-1.037206
24	8	0	-3.640889	-0.711054	-0.867218
25	1	0	1.866376	-2.388061	-2.981510
26	1	0	2.936484	-0.344779	-3.947304
27	1	0	2.213852	1.910403	-3.138030
28	1	0	0.920460	3.193797	-1.393220
29	1	0	-0.694411	2.624688	-1.826989
30	1	0	0.553483	2.600299	2.077134
31	1	0	1.607823	3.199090	0.792131
32	1	0	3.714847	1.929558	1.237369
33	1	0	4.807003	-0.313066	1.433394
34	1	0	3.381370	-2.369168	1.427582
35	1	0	1.096474	-3.330523	1.021916
36	1	0	0.150760	-2.505451	2.260697
37	1	0	-1.097056	-2.554664	-1.629701
38	1	0	0.422774	-3.325176	-1.161582
39	1	0	-1.456285	3.333797	1.499094
40	1	0	-0.564127	4.368313	0.344181
41	1	0	-1.966928	3.391485	-0.190439
42	1	0	-1.474956	-4.034810	0.656022
43	1	0	-1.998058	-2.700486	1.732856
44	1	0	-2.537854	-2.729288	0.047290
45	1	0	-1.046188	-0.342826	2.934502
46	8	0	-3.098791	1.398729	0.778671
47	1	0	-3.168744	1.099982	1.706363
48	1	0	-3.834389	0.962649	0.313315

[7]INT3 *S* = 3

Center Number	Atomic Number	Atomic Type	Coordinates (Angstroms) X	Y	Z
1	6	0	1.548824	1.105183	-2.740116
2	6	0	0.579175	1.136635	-1.744470

3	7	0	0.066366	0.007170	-1.229753
4	6	0	0.526860	-1.191279	-1.617609
5	6	0	1.503067	-1.295791	-2.606784
6	6	0	2.005546	-0.134001	-3.183695
7	6	0	0.021471	2.404537	-1.162078
8	7	0	-0.168345	2.308374	0.302994
9	6	0	-0.991893	3.444733	0.759374
10	26	0	-1.165826	0.149651	0.562197
11	8	0	-1.908018	0.040461	2.254088
12	6	0	-0.078887	-2.401379	-0.955673
13	7	0	-0.537142	-2.154863	0.429803
14	6	0	-1.640715	-3.068729	0.779989
15	6	0	0.552863	-2.357053	1.405455
16	6	0	1.561080	-1.251743	1.345324
17	7	0	1.026321	-0.025069	1.262664
18	6	0	1.822392	1.042101	1.128051
19	6	0	3.210927	0.922099	1.116879
20	6	0	3.773005	-0.344470	1.243123
21	6	0	2.937773	-1.453766	1.352166
22	6	0	1.117984	2.363013	1.032316
23	8	0	-2.504434	-0.988530	-1.014757
24	8	0	-3.598521	-0.478981	-1.451828
25	1	0	1.867769	-2.274780	-2.911129
26	1	0	2.767638	-0.192206	-3.957899
27	1	0	1.946725	2.031576	-3.148856
28	1	0	0.655730	3.268249	-1.422973
29	1	0	-0.970930	2.593109	-1.594836
30	1	0	0.886895	2.697496	2.052939
31	1	0	1.779510	3.130803	0.597309
32	1	0	3.836582	1.805046	1.001613
33	1	0	4.853788	-0.468856	1.234578
34	1	0	3.344807	-2.460363	1.423532
35	1	0	1.031158	-3.340239	1.260460
36	1	0	0.089572	-2.373133	2.401588
37	1	0	-0.956952	-2.701890	-1.539598
38	1	0	0.630225	-3.245445	-0.989829
39	1	0	-1.243373	3.302101	1.814636
40	1	0	-0.448426	4.395647	0.646561

41	1	0	-1.916278	3.484571	0.184189
42	1	0	-1.302936	-4.116743	0.789582
43	1	0	-2.018248	-2.801540	1.770917
44	1	0	-2.449720	-2.957626	0.056640
45	1	0	-1.436346	-0.180503	3.067714
46	8	0	-2.917165	1.376615	0.191428
47	1	0	-3.334130	1.298429	1.067852
48	1	0	-3.497144	0.864382	-0.436481
